# Supplementary material for: Dissecting the polygenic basis of atherosclerosis via disease-associated cell state signatures
Source: Am J Hum Genet. 2023 Apr 14;110(5):722–40. doi: 10.1016/j.ajhg.2023.03.013 (PMC10183377; doi:10.1016/j.ajhg.2023.03.013)
Supplement: Document S1. Supplemental methods and Figures S1–S30 [file mmc1.pdf]

The American Journal of Human Genetics, Volume *110*

Supplemental information

Dissecting the polygenic basis of atherosclerosis via disease-associated cell state signatures

Tiit Örd, Tapio Lönnberg, Valtteri Nurminen, Aarthi Ravindran, Henri Niskanen, Miika Kiema, Kadri Õunap, Maleeha Maria, Pierre R. Moreau, Pashupati P. Mishra, Senthil Palani, Jenni Virta, Heidi Liljenbäck, Einari Aavik, Anne Roivainen, Seppo Ylä-Herttuala, Johanna P. Laakkonen, Terho Lehtimäki, and Minna U. Kaikkonen

Supplemental Figures

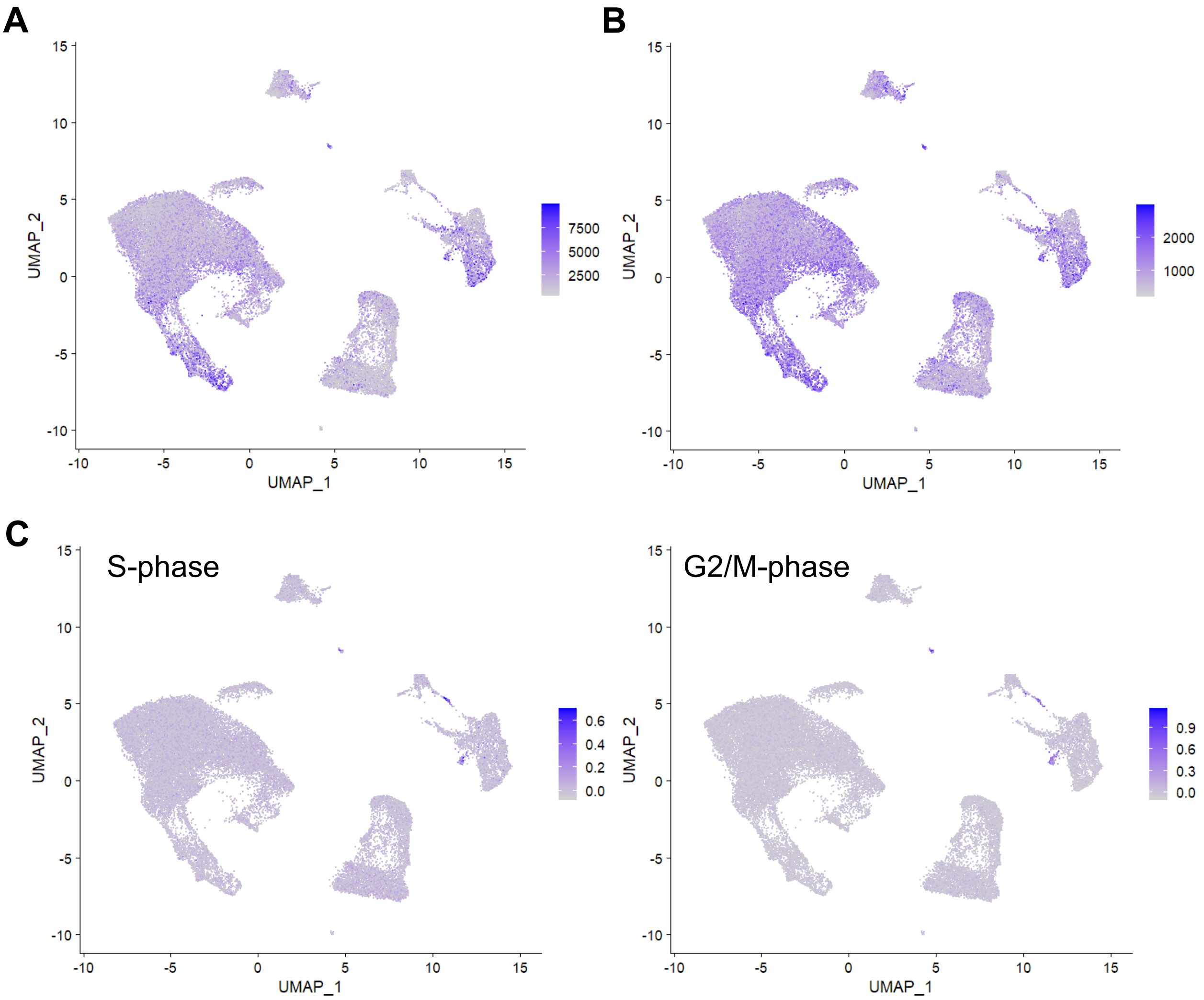

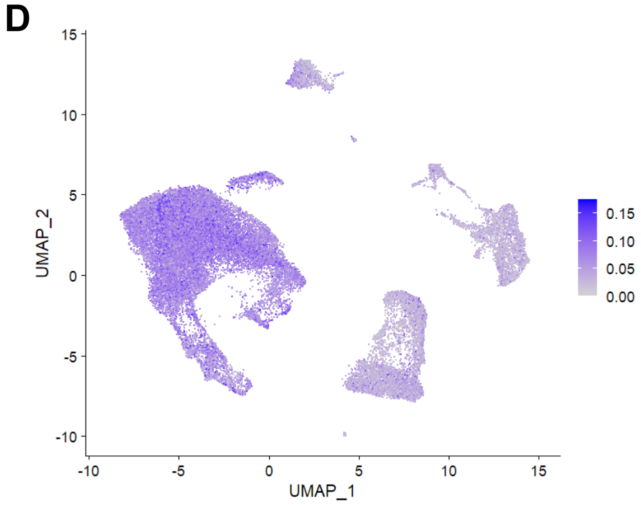

Figure S1. Quality Assessment of the scRNA-Seq data. (A) nCount, unique molecular identifiers (UMIs); **(B)** nFeature, number of genes; **(C)** assignment of cell cycle scores for S and G2/M phases **(D)** percentage of mitochondrial gene UMIs.

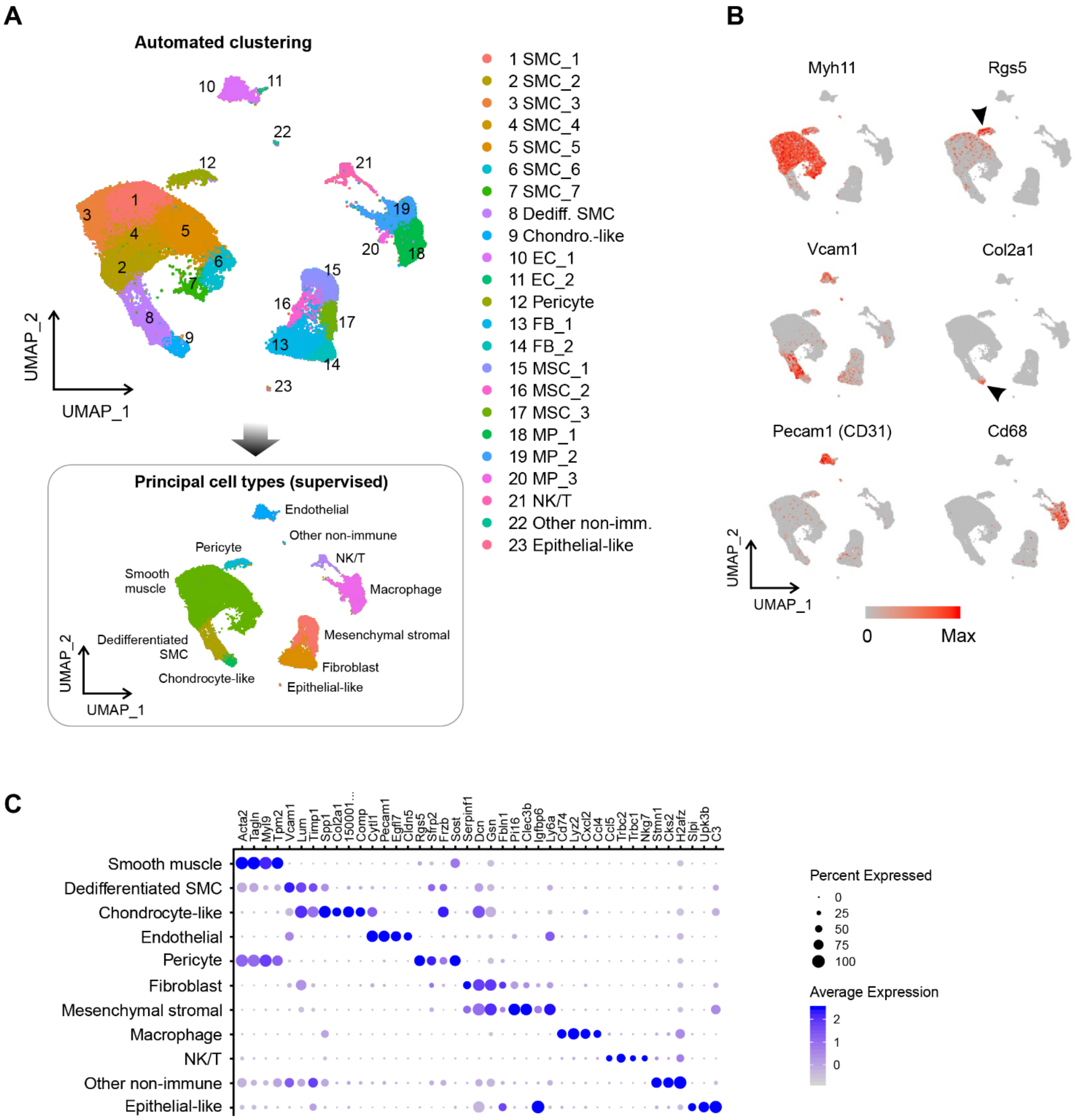

Figure S2. Clustering and identification of cell types in scRNA-Seq of mouse atherosclerotic lesion. (A) UMAP projection of the scRNA-Seq profiles represented as 23 clusters identified using automated clustering and the eleven manually annotated populations. (**B**) UMAP plots showing the expression of selected markers used to annotate the cell types. (**C**) Dot plot demonstrating the top four marker genes for each lesional cell type. Dot size corresponds to the proportion of cells within the cluster that expressed the gene, and dot color intensity corresponds to the average expression level.

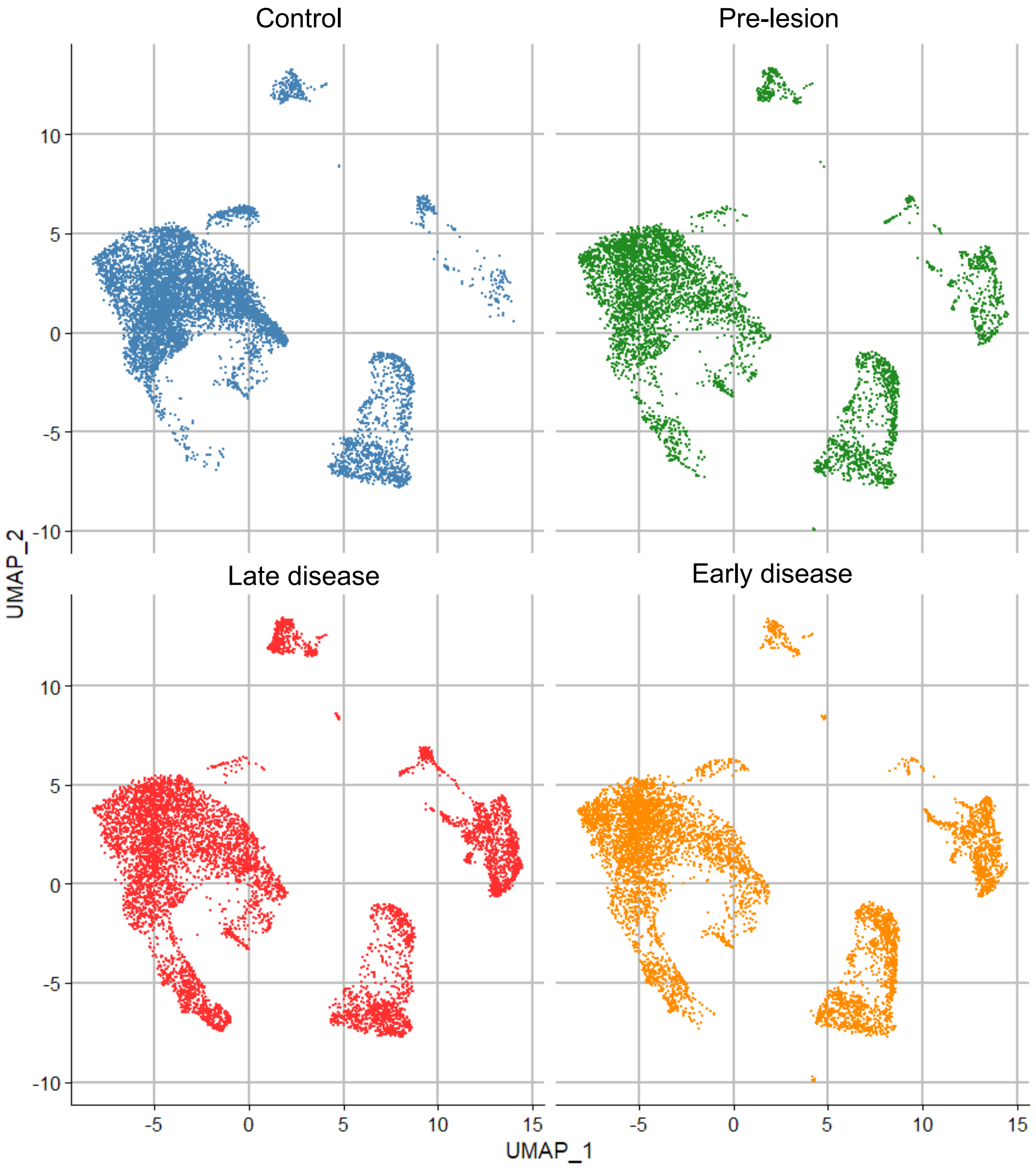

Figure S3. Changes in the cell numbers during progression of atherosclerosis. UMAP projection of the scRNA-Seq profiles separately for each disease condition.

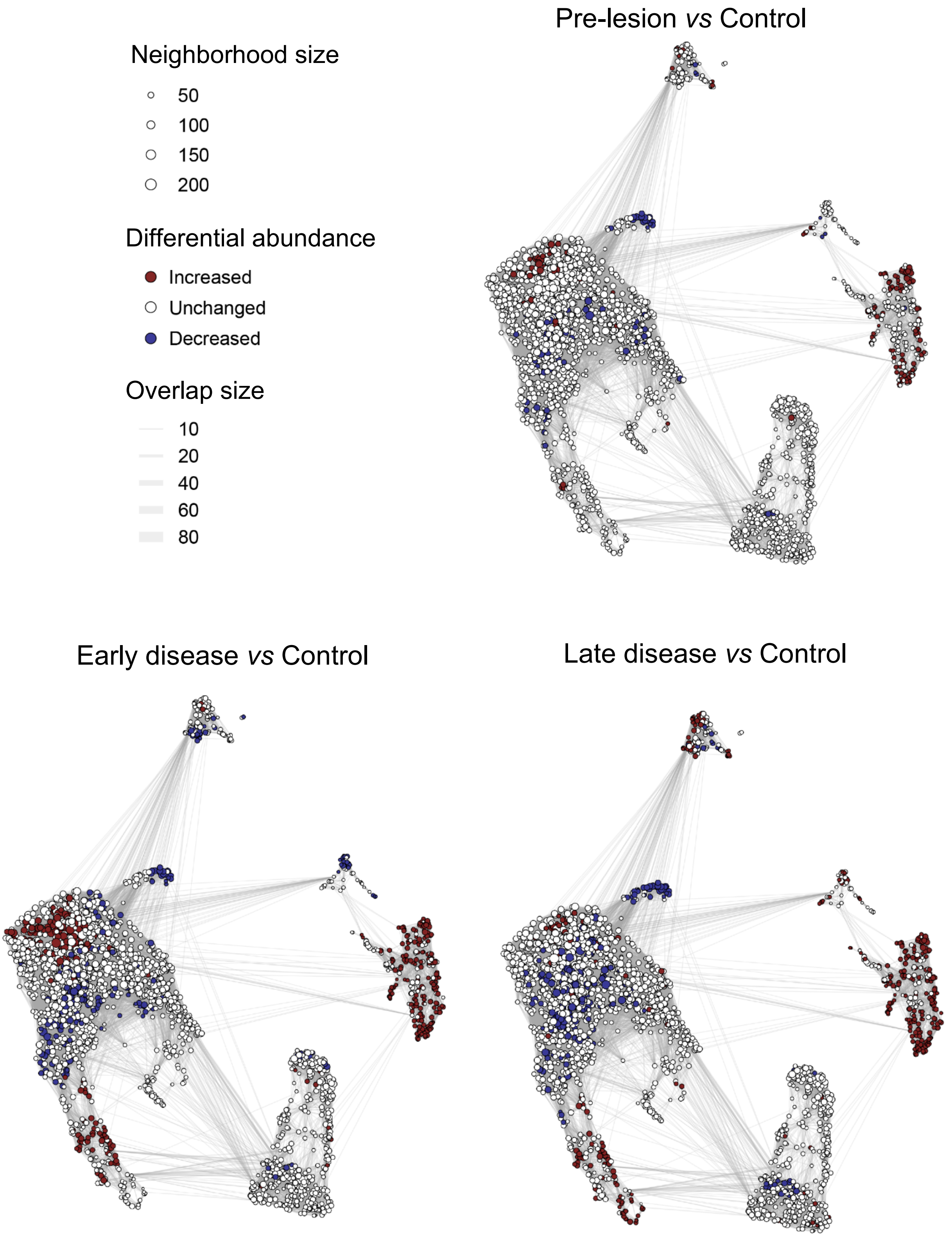

Figure S4. Differentially abundant cellular neighborhoods based on *k*-nearest neighbor graph analysis (Milo[1]). Each stage of disease was compared to control, and significantly increased or decreased neighborhoods (SpatialFDR < 0.1) are indicated by color. The method allows partially overlapping neighborhoods, and the thickness of the lines connecting neighborhoods indicates the number of overlapping cells. The overlap is taken into account when calculating significance in the SpatialFDR procedure.

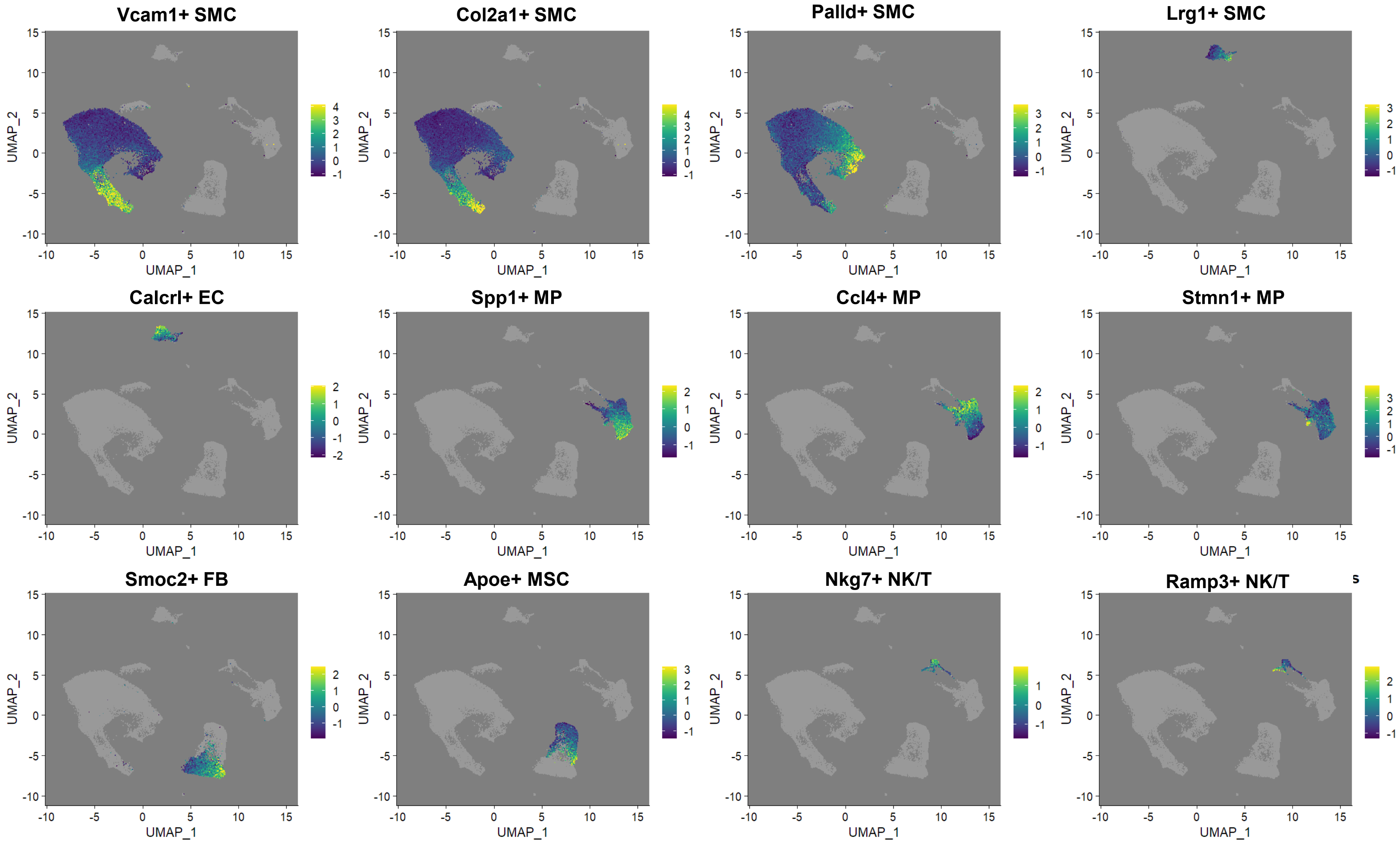

Figure S5. Cell state gene program activity as standard deviation (SD). Cell state marker genes were used as gene sets in the AddModuleScore function of Seurat (expression bin-based averaging). The module scores are presented normalized to the score SD within the cell type.

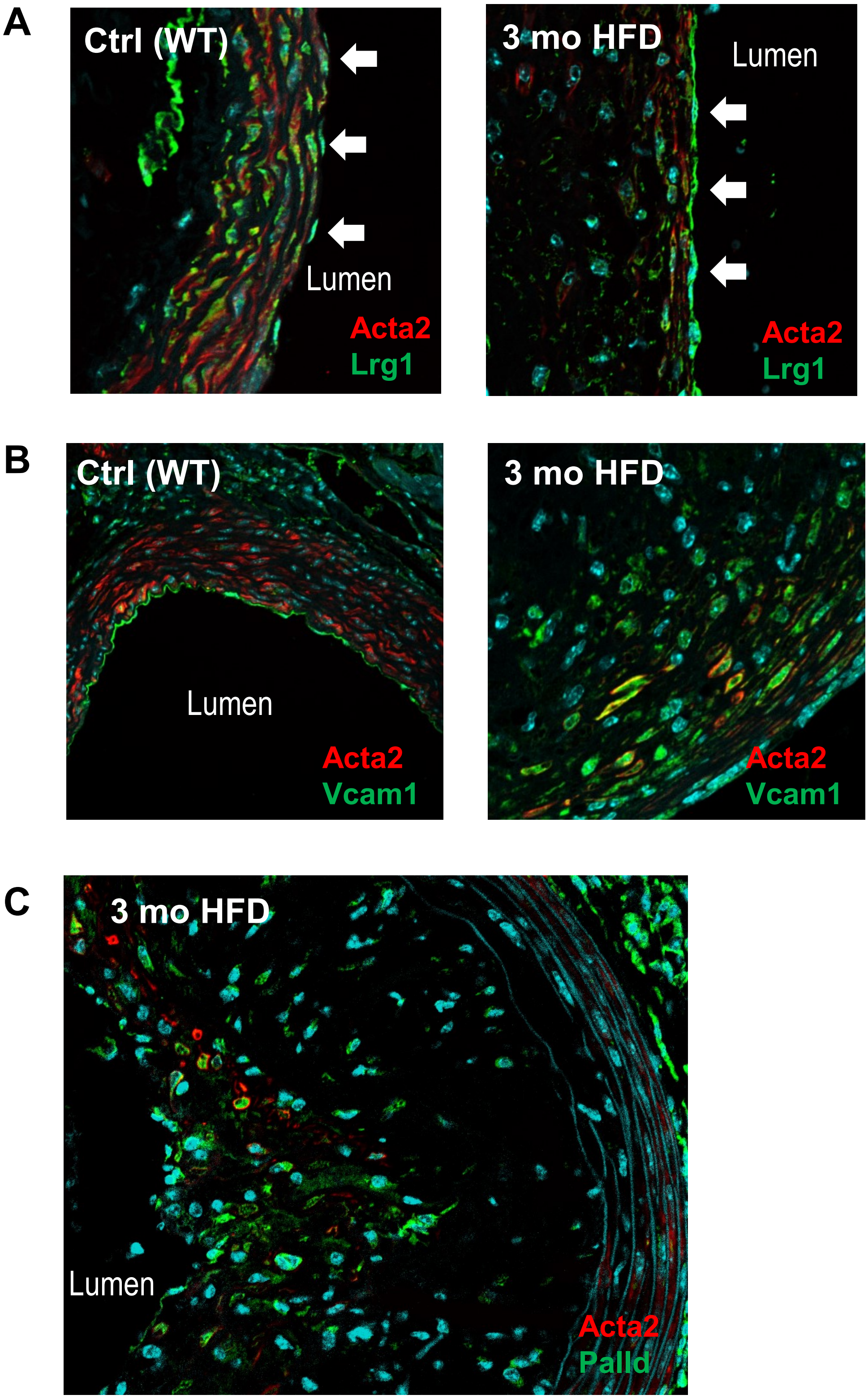

Figure S6. Immunohistological validation of identified cell state marker genes. Representative images of (**A**) Lrg1, (**B**) Vcam1 and (**C**) Palld (all in green) staining in wild type chow diet-fed and *Ldlr*$^{-/-}$/*Apob*$^{100/100}$ 3-month high fat diet mice. The smooth muscle cells are stained with Acta2 antibody (red) and DAPI staining is shown in cyan.

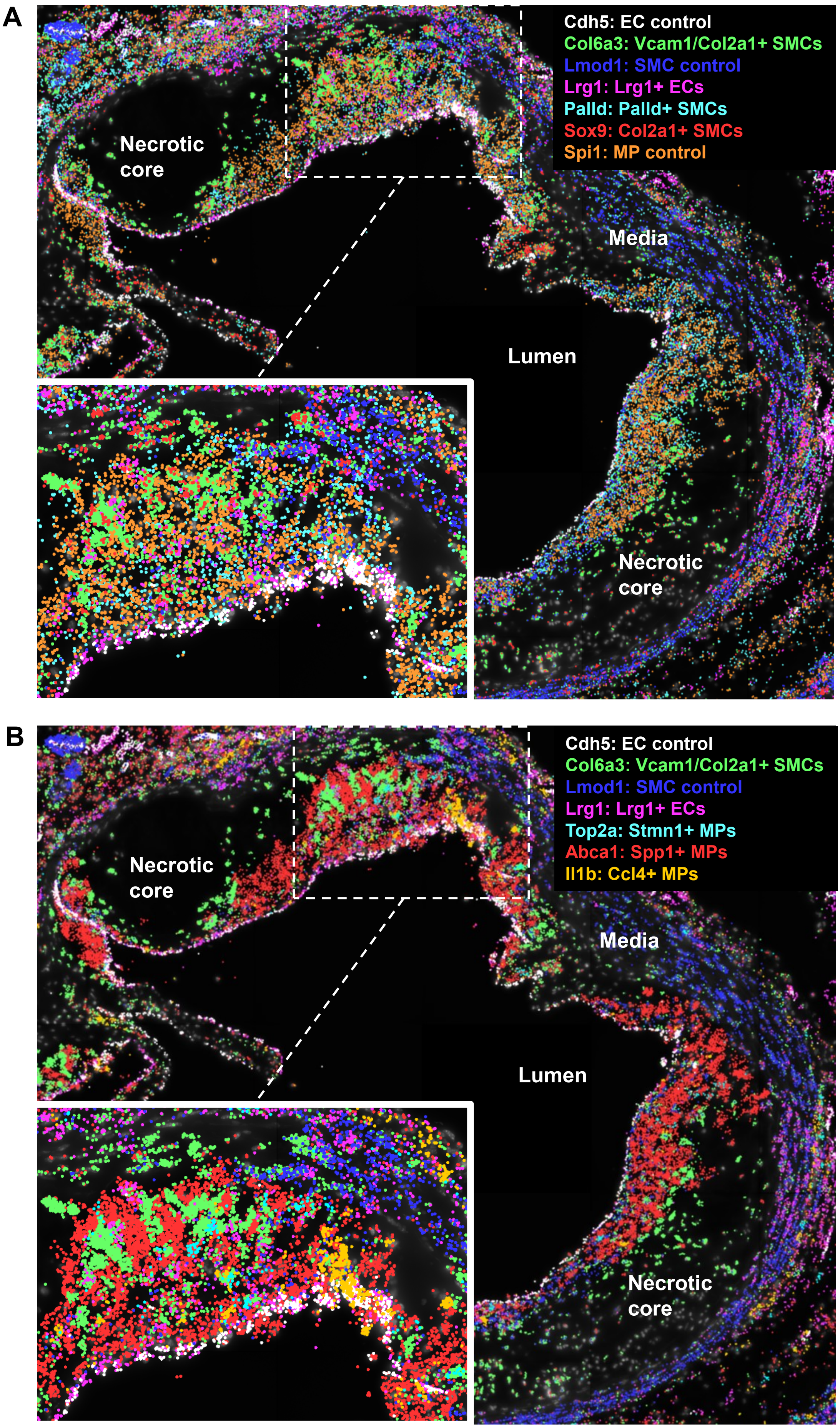
A
Cdh5: EC control
Col6a3: Vcam1/Col2a1+ SMCs
Lmod1: SMC control
Lrg1: Lrg1+ ECs
Palld: Palld+ SMCs
Sox9: Col2a1+ SMCs
Spi1: MP control
Necrotic core
Media
Lumen
Necrotic core
B
Cdh5: EC control
Col6a3: Vcam1/Col2a1+ SMCs
Lmod1: SMC control
Lrg1: Lrg1+ ECs
Top2a: Stmn1+ MPs
Abca1: Spp1+ MPs
Il1b: Ccl4+ MPs
Necrotic core
Media
Lumen
Necrotic core

Figure S7. Molecular Cartography based identification of selected cell states. Distribution of seven selected genes representing the Lrg1+ ECs and (**A**) the three SMC cell states and (**B**) the three MP cell states along with established cell type markers (*Cdh5*, *Lmod1* and *Spi1*) in the aortic root of $Ldlr^{-/-}/Apob^{100/100}$ mice (3-month high fat diet). Each dot represents a single RNA molecule and each pixel equals 138 nm. Insert on the left corner represents magnification of the part of image marked with a dashed line.

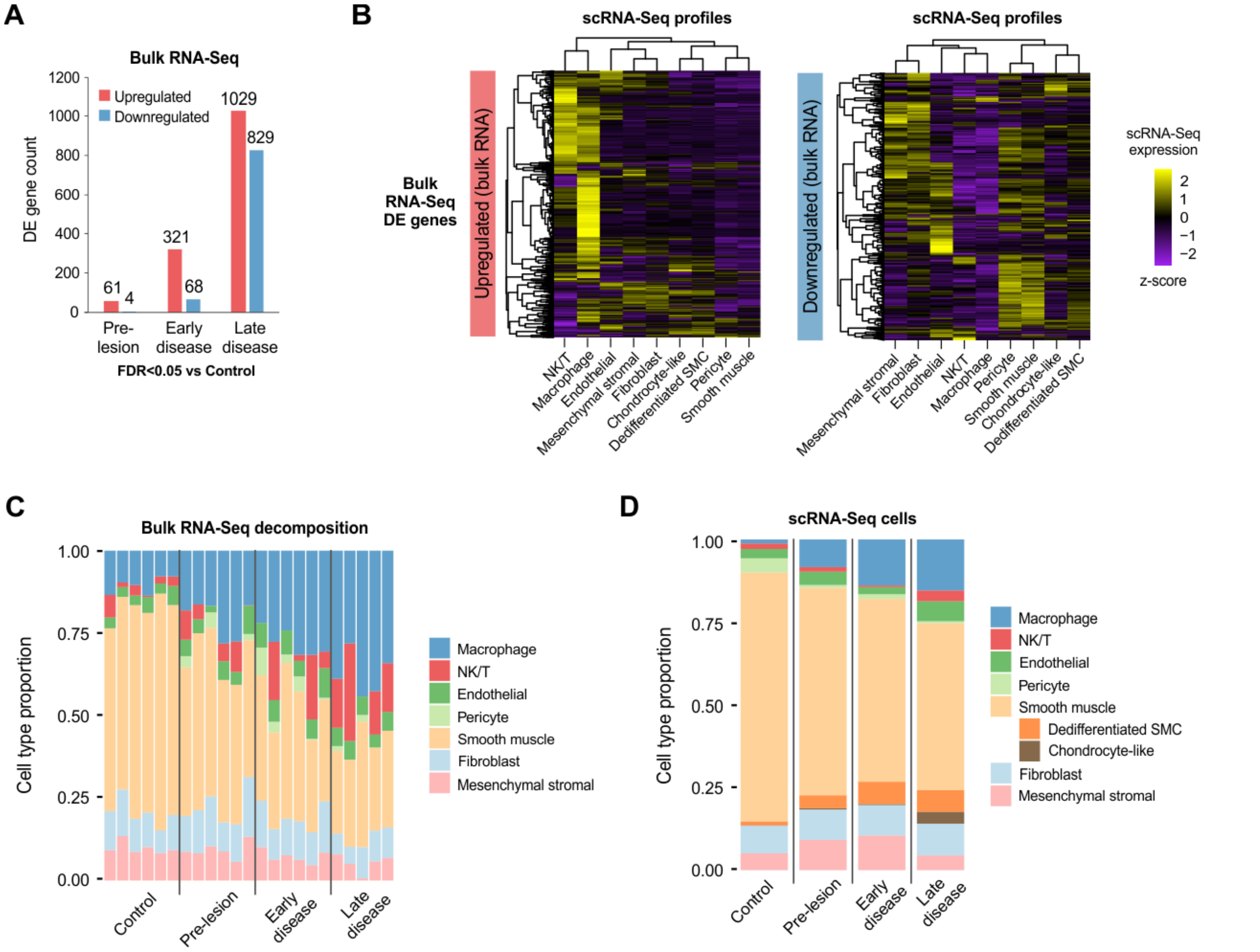

Figure S8. Analysis of cell type proportions from bulk transcriptomics data. (**A**) Number of differentially expressed genes identified from bulk RNA-Seq analysis during different stages of disease progression (see Figure 1A). (**B**) Cell types expressing the genes detected as differentially expressed in bulk RNA-Seq comparison of disease stages. Expression levels of differentially expressed genes from bulk RNA-seq analysis (panel A) are plotted from the cell types identified in scRNA-Seq. (**C**) Computational prediction of cell type composition from the bulk RNA-Seq using the scRNA-Seq data as reference. (**D**) Cell type proportions detected in the scRNA-seq data.

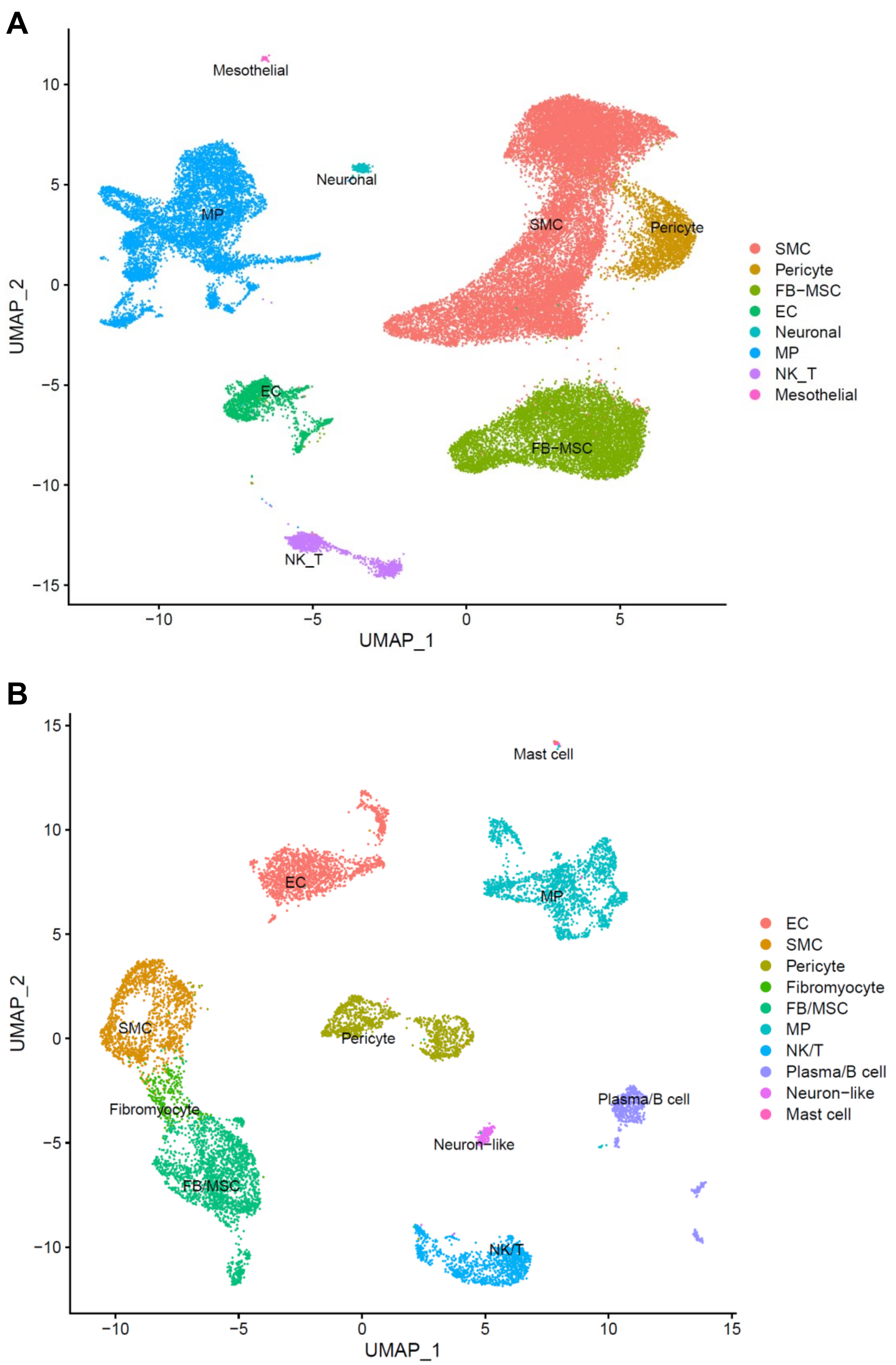

Figure S9. Reprocessing of public scRNA-Seq datasets for cell type identification (A) Reprocessing of mouse scRNA-Seq dataset from Pan *et al*[2] using *Ldlr*$^{-/-}$ and *Apoe*$^{-/-}$ mouse models. **(B)** Reprocessing of scRNA-Seq dataset from four human atherosclerotic coronary arteries[3].

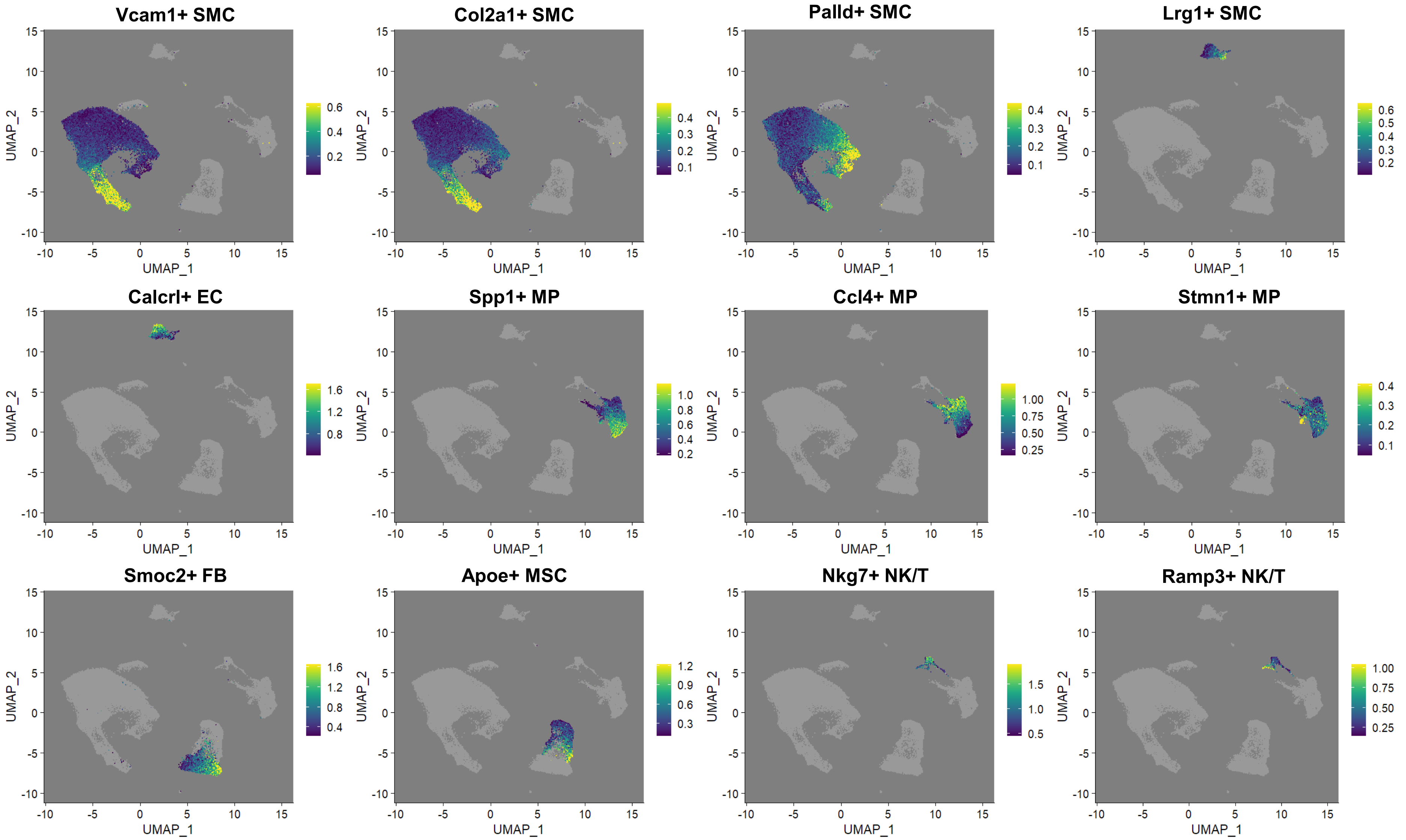

Figure S10. Atherosclerotic cell state marker gene set enrichment scoring. Module scores shown in the target cell type highlights subpopulations of cells with high expression of disease associated markers.

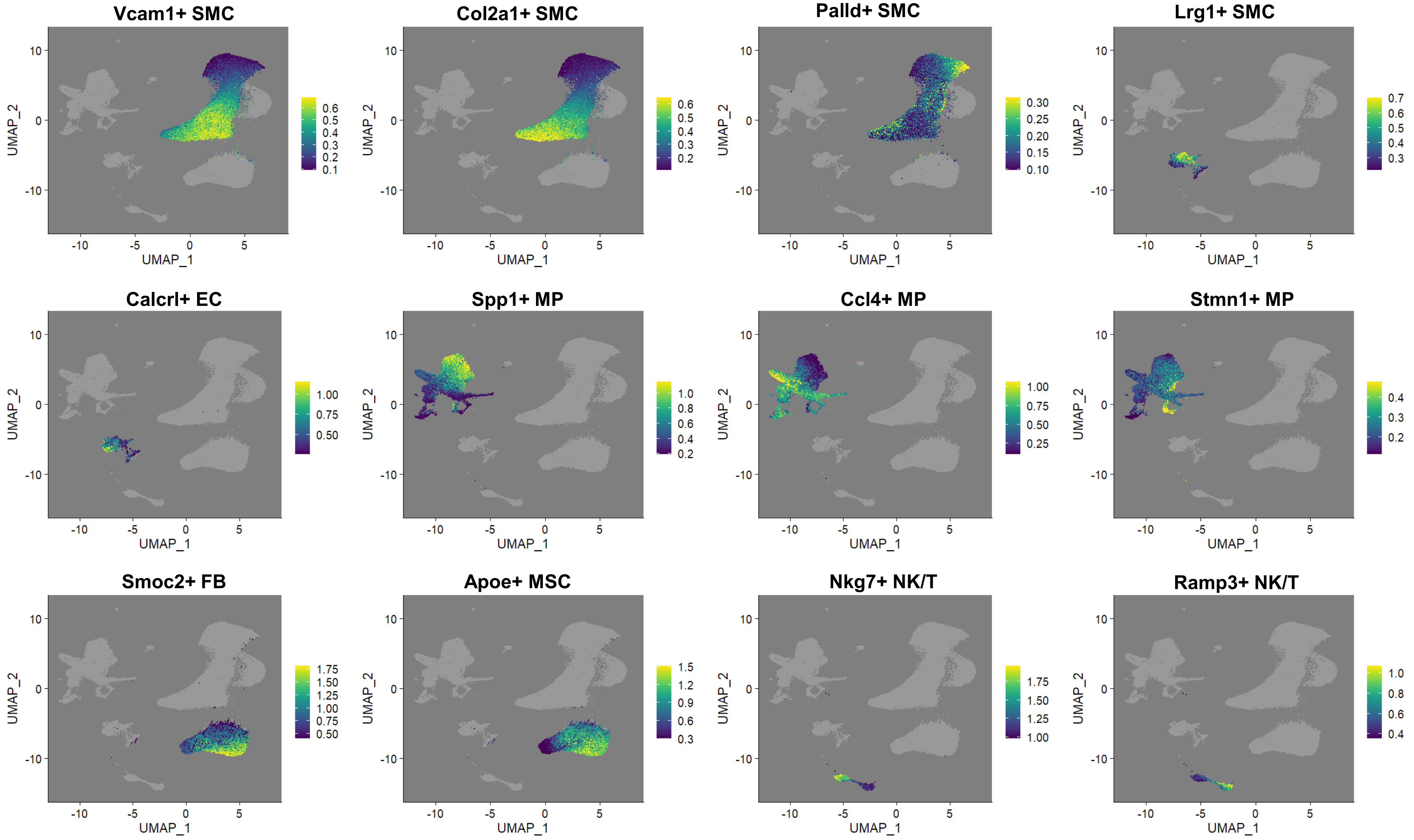

Figure S11. Atherosclerotic cell state marker gene set enrichment scoring in the Pan *et al*[2] *Ldlr*$^{-/-}$ and *Apoe*$^{-/-}$ mouse models scRNA-Seq dataset. Module summary scores (Seurat AddModuleScore; expression bin-based average log fold change) are visualized on a UMAP of the dataset in the cell type of interest (for cluster identifies, see Figure S9A).

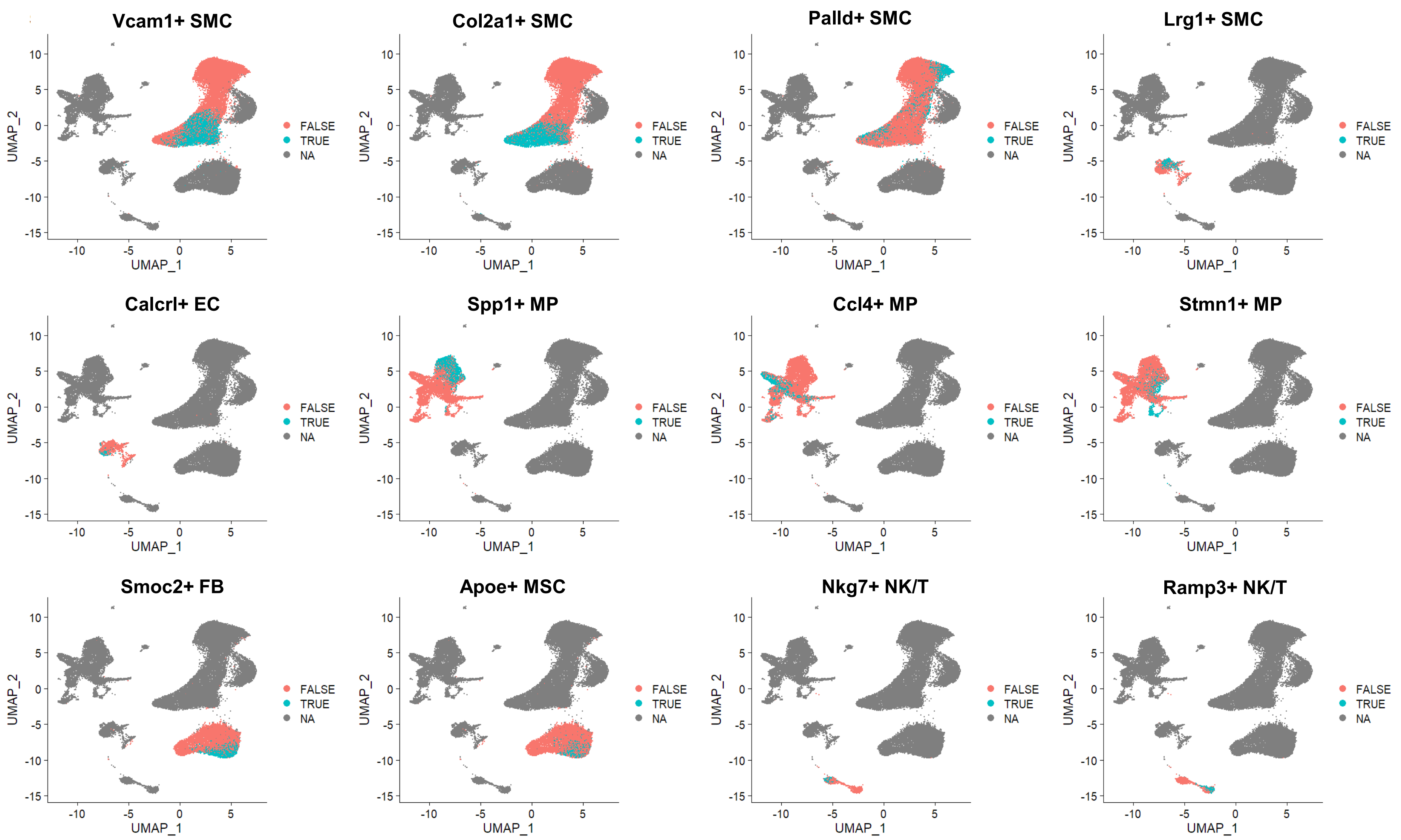

Figure S12. Atherosclerotic cell state marker gene set enrichment scoring in the Pan *et al*[2] dataset. Cells were classified as either positive or negative for the gene program using a cutoff of >1 standard deviation. This binary classification of the cells used to generate Figure S13.

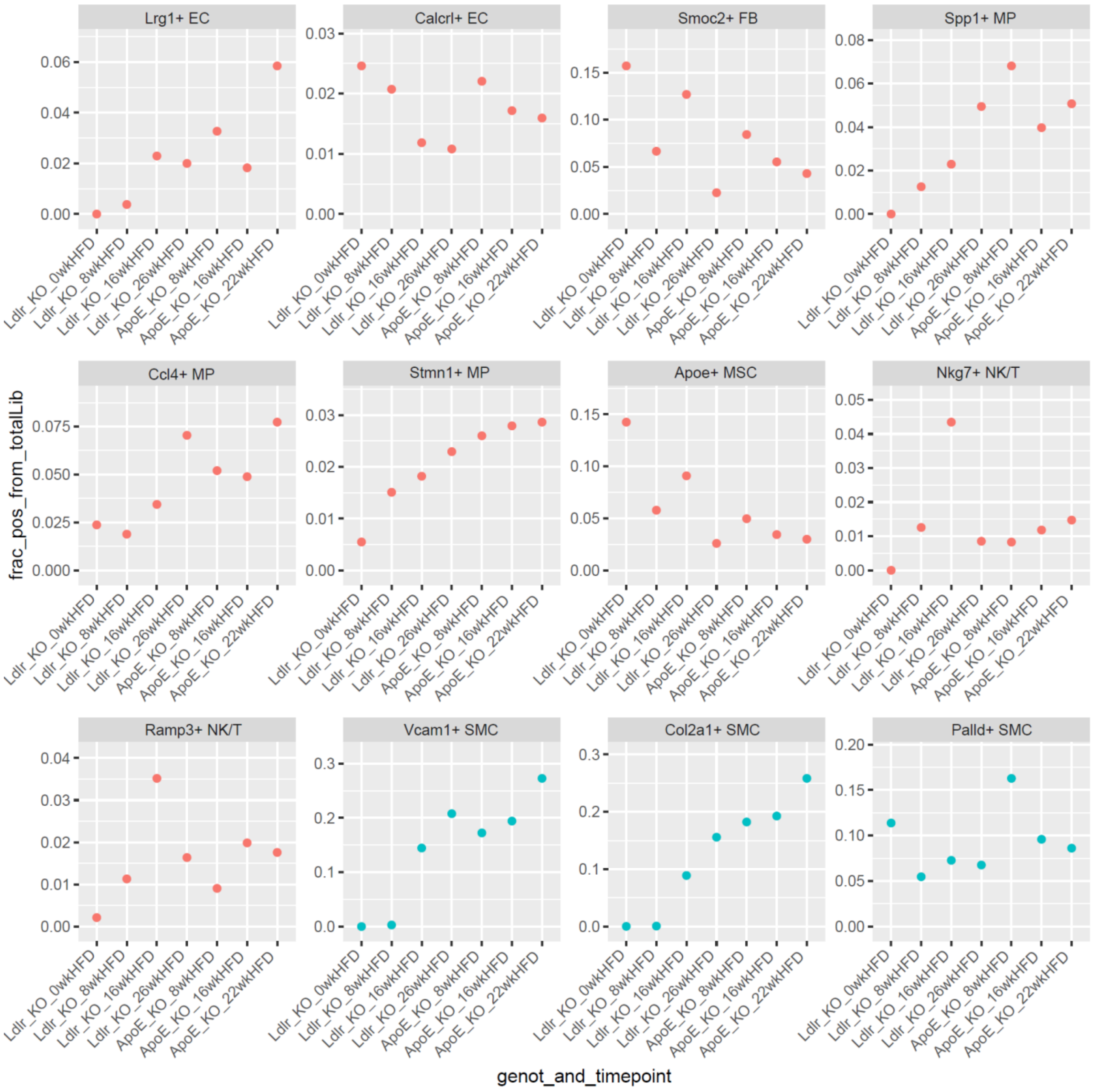

Figure S13. Relative changes in the cell state proportions in different models and timepoints of atherosclerosis in the Pan *et al*[2] dataset. Fraction of cell state-positive cells (as defined in Figure S12) from the total library are presented. SMC-related cell states are shown from *Myh11* (*ZsGreen*) lineage-positive libraries (blue dots), while all others are shown from *Myh11* (*ZsGreen*) lineage-negative libraries (red dots).

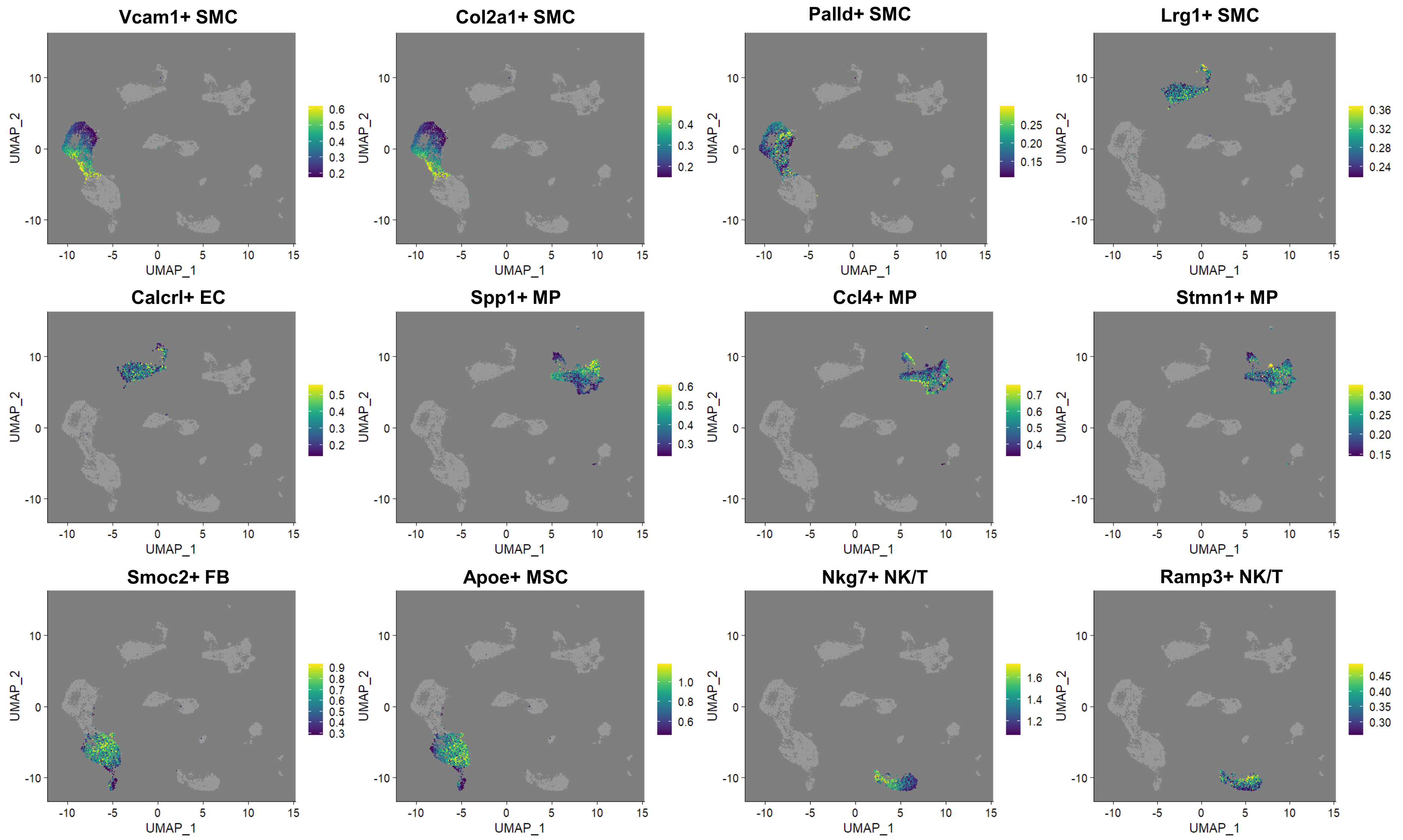

Figure S14. Atherosclerotic cell state marker gene set enrichment scoring in the Wirka *et al*[3] human coronary artery scRNA-Seq dataset. Module summary scores (Seurat AddModuleScore; expression bin-based average log fold change) are visualized on a UMAP in the target cell cluster (for cluster identities, see Figure S9B).

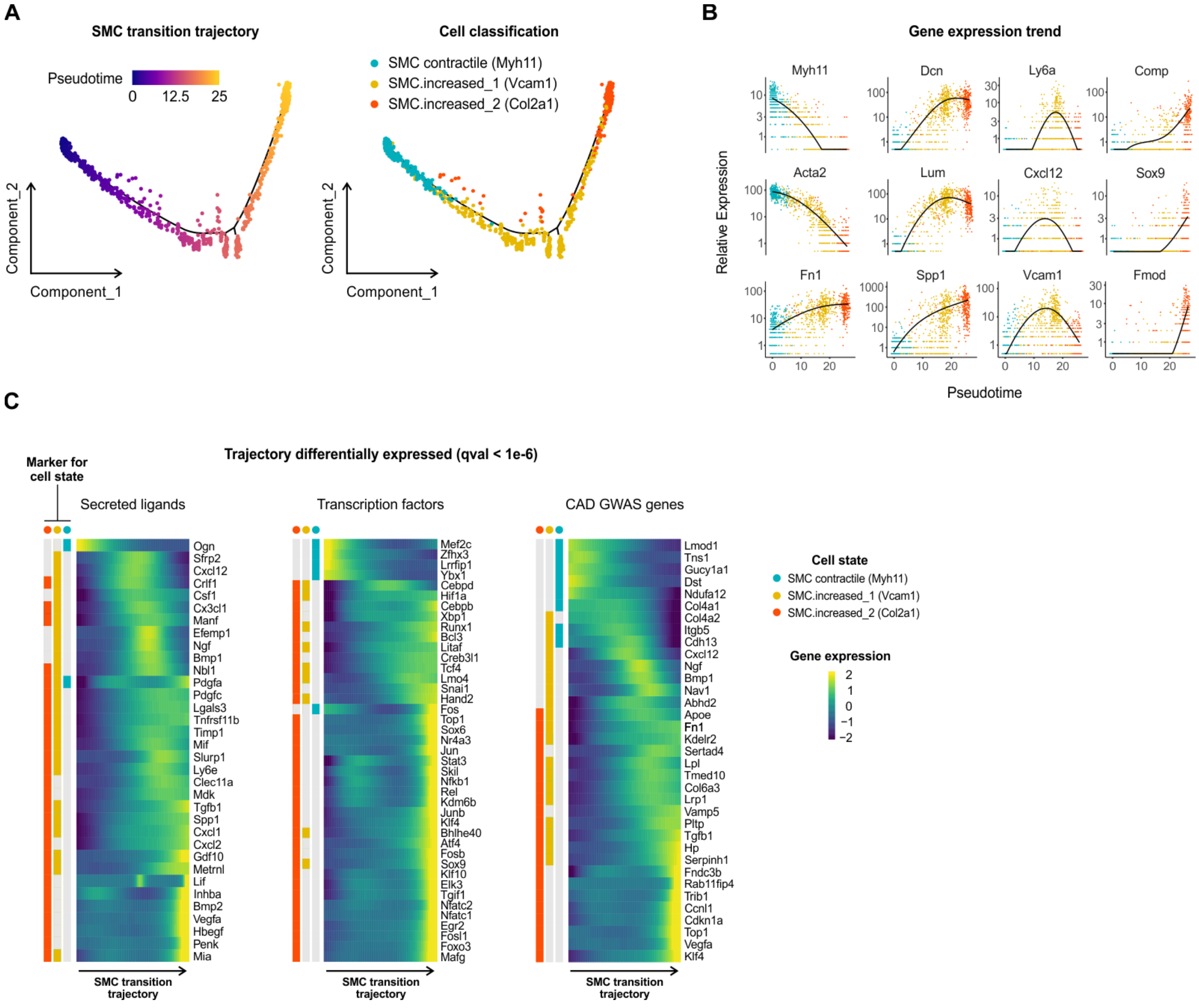

Figure S15. Pseudotime analysis of SMC cell states from atherosclerotic aorta. (**A**) scRNA-Seq trajectory analysis of SMC states colored by pseudotime and cell state assignment. (**B**) Gene expression changes of selected marker genes along the pseudotime trajectory. Cells are colored by cell state as in panel A. (**C**) Expression changes of secreted ligands, transcription factors and CAD GWAS genes that display differential expression along the pseudotime trajectory.

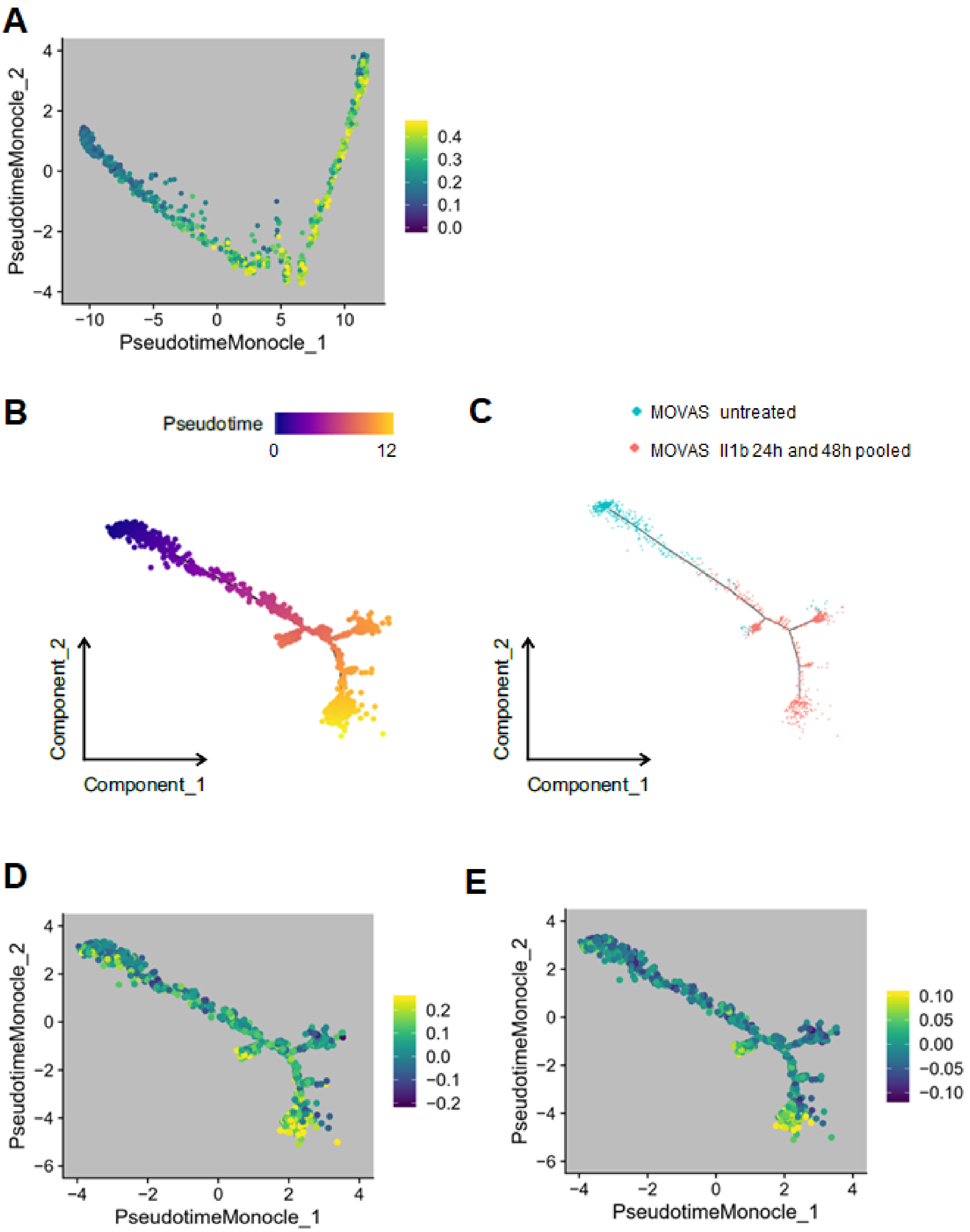

Figure S16. Projection of IL-1β responsive genes on *in vivo* SMC disease response pseudotime trajectory. (A) Differentially expressed genes identified using scRNA-seq of *in vitro* SMCs (MOVAS cells) upon 24 and 48 h IL-1β treatment plotted on the *in vivo* SMC trajectory (see Figure S15A) as a gene set activity score. scRNA-Seq trajectory analysis of *in vitro* SMCs under basal and IL-1β treatment conditions colored by (**B**) pseudotime and (**C**) treatment. (**D**) Vcam1+ SMC and (**E**) Col2a1+ SMC marker gene sets plotted as activity scores on the *in vitro* SMC IL-1β response trajectory (described in panels B-C).

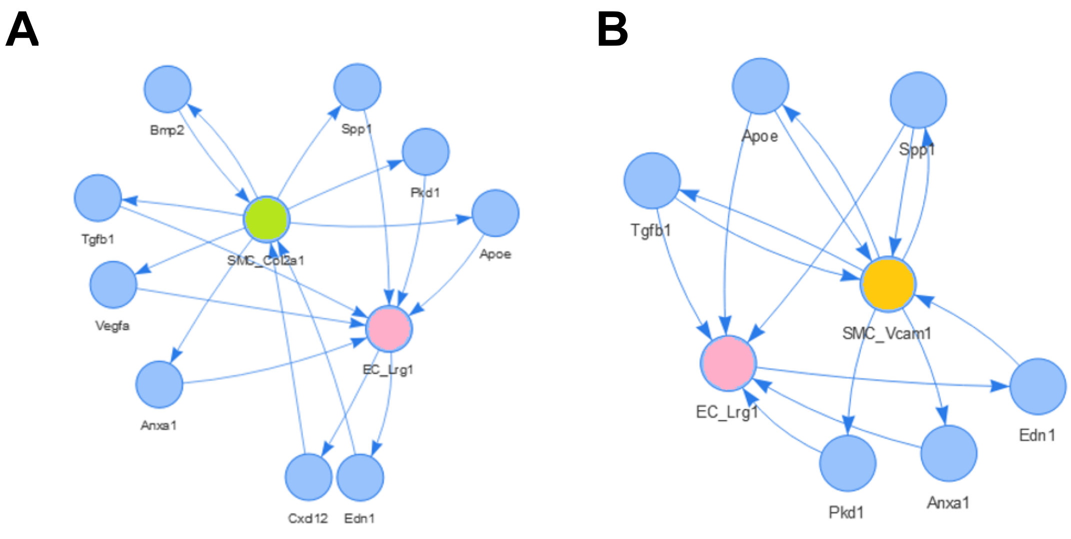

Figure S17. Prediction of cell-cell signaling networks. Ligands predicted to mediate paracrine and autocrine signaling between Lrg1+ ECs and either (**A**) Col2a1+ SMCs or (**B**) Vcam1+ SMCs.

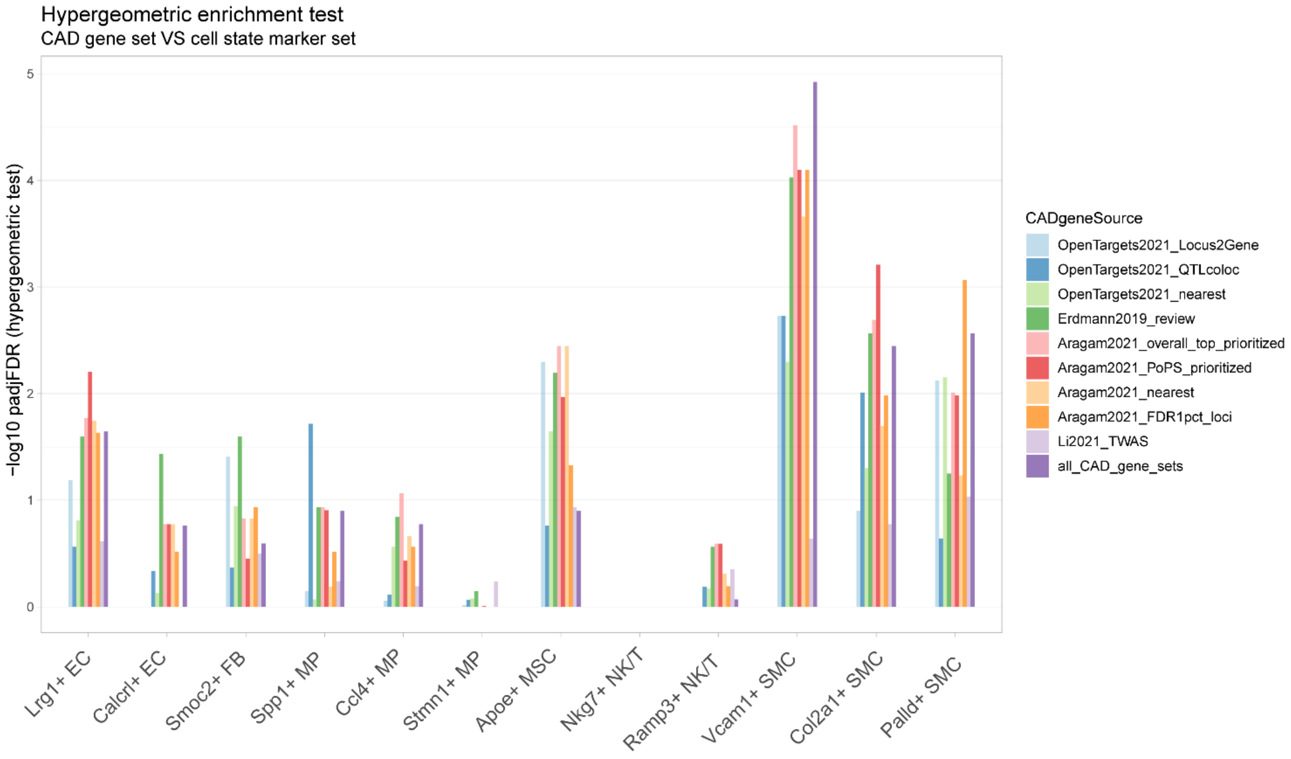

Figure S18. Enrichment of CAD GWAS genes within the disease associated cell states gene signatures. Hypergeometric enrichment test results are shown for CAD GWAS candidate causal gene lists from 9 different sources separately or all combined (different colors). The set of background genes for enrichment testing was all genes expressed at >1 TPM in at least one cell type or cell state in aorta scRNA-Seq (total 14902 genes).

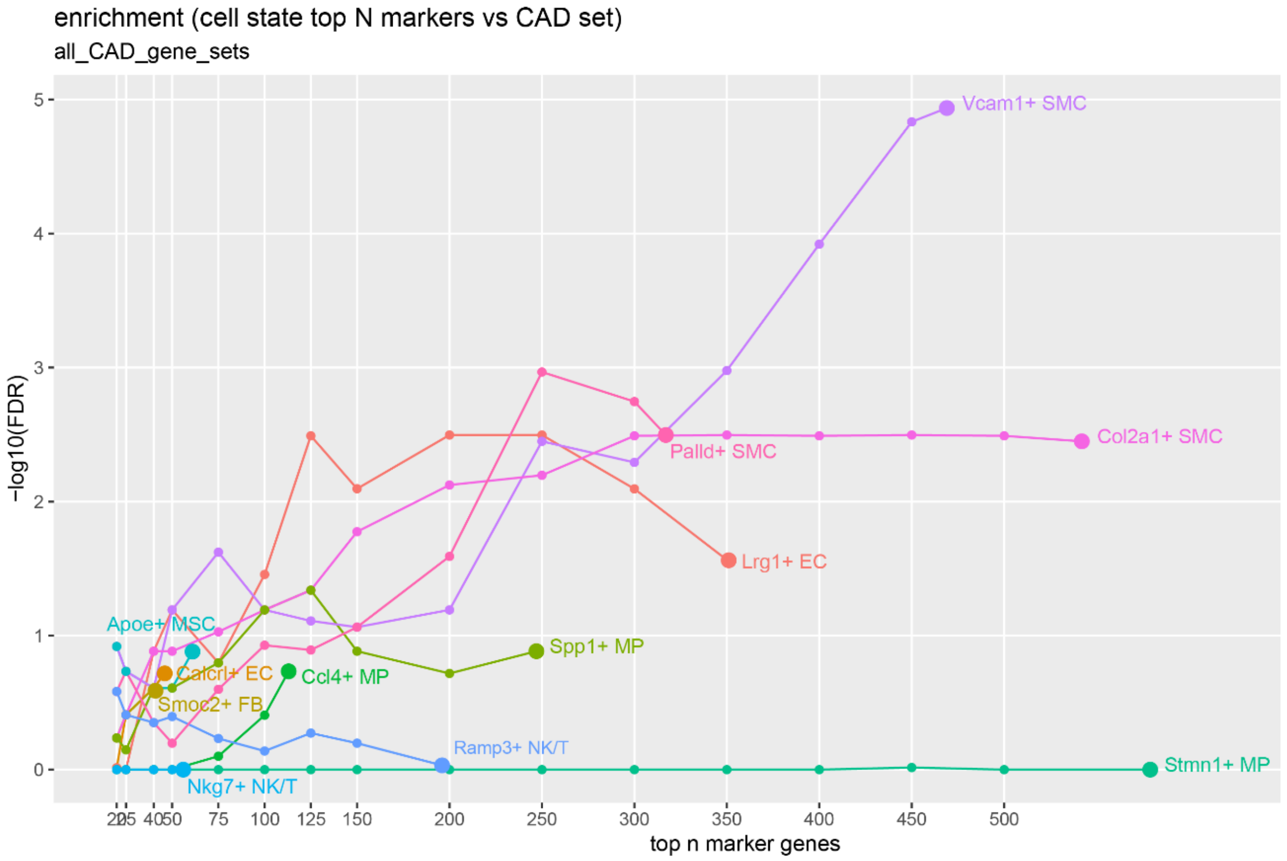

Figure S19. Enrichment of CAD GWAS prioritized genes in cell state marker sets truncated to a specific gene count. The indicated number of top markers were selected for each cell state (up to the total number of marker genes available). The set of CAD GWAS genes was prioritized genes from all 9 sources combined (Methods). The set of background genes for enrichment testing was all genes expressed at >1 TPM in at least one cell type or cell state in aorta scRNA-Seq (total 14902 genes).

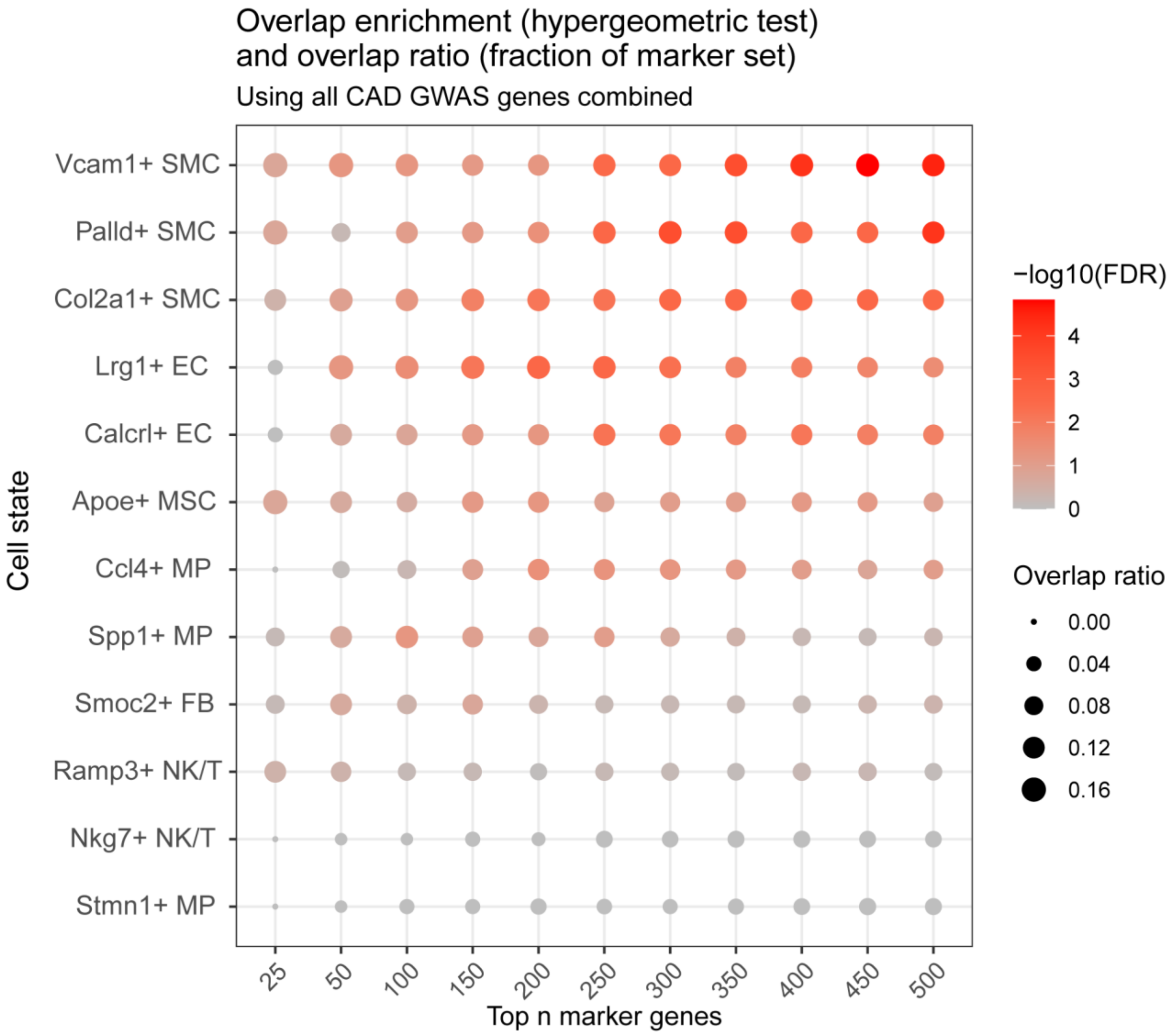

Figure S20. Enrichment of CAD GWAS prioritized genes in cell state marker lists equalized for gene count by including sub-threshold marker genes. The indicated number of top markers were selected for each cell state using log fold change ranking. The required number of top genes were selected irrespective of whether the marker gene criteria (log fold change > 0.25 and FDR < 0.05; Methods) were fulfilled. Overlap enrichment was tested by hypergeometric test. The set of CAD GWAS genes was prioritized genes from all 9 sources combined (see Methods). The set of background genes for enrichment testing was all genes expressed at >1 TPM in at least one cell type or cell state in aorta scRNA-Seq (total 14902 genes). Column and row order is by average -log10(FDR). The dot size indicates the ratio of CAD GWAS genes within the marker gene set.

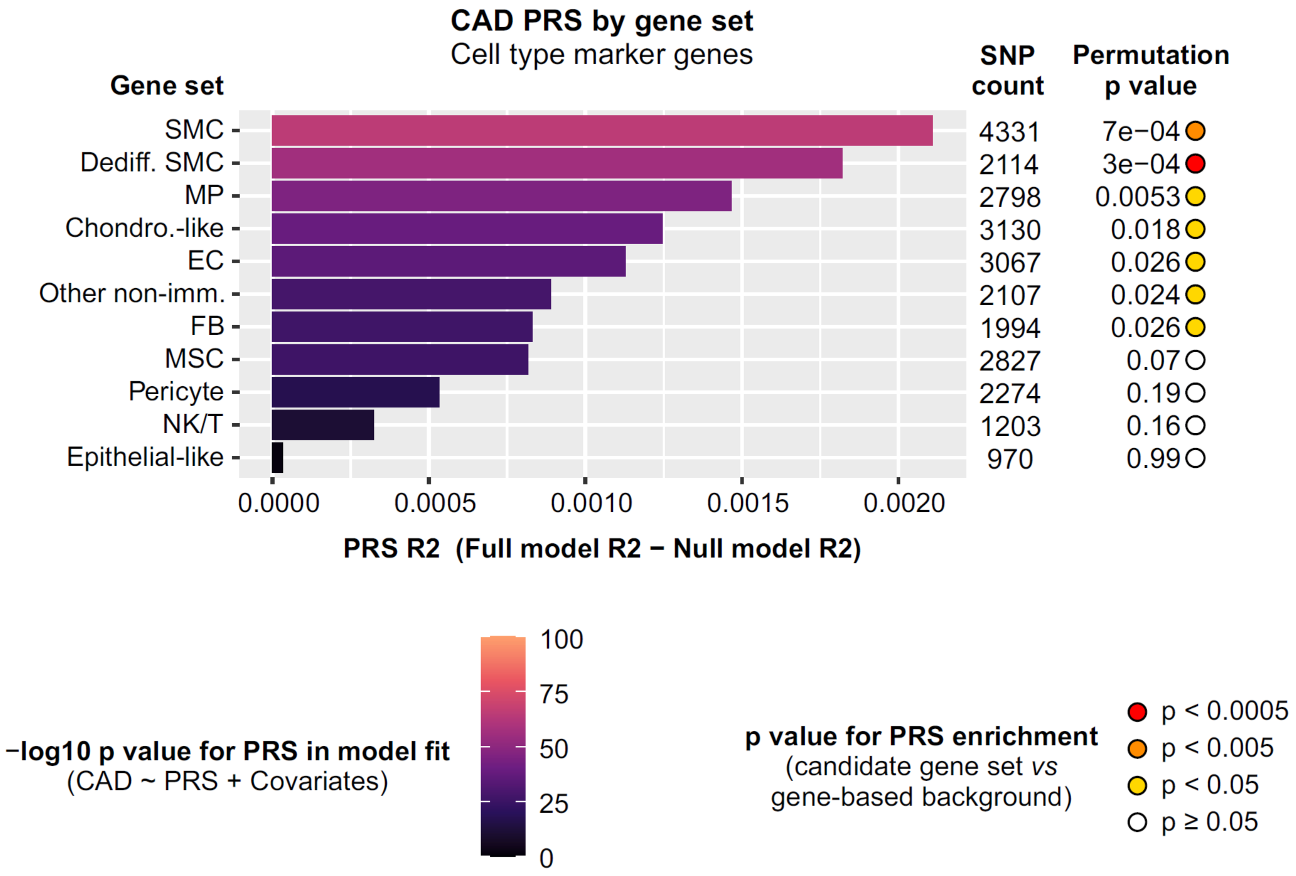

Figure S21. Proportion of variance of CAD explained by the cell type specific marker genes. PRS was constructed using the cell type specific marker gene coordinates (-35 kb upstream to 10 kb downstream; gene list in Table S1) using PRSet[4].

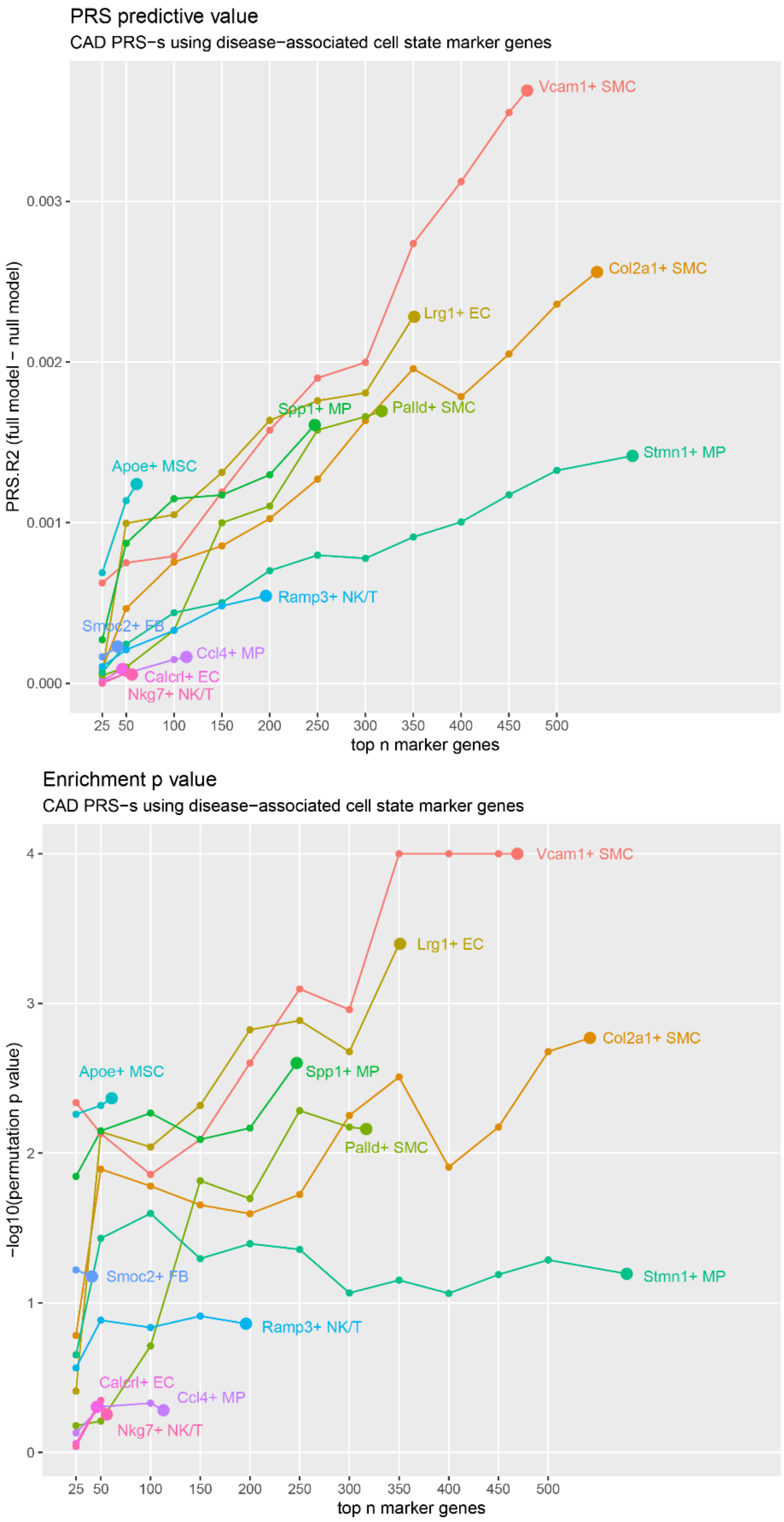

Figure S22. Evaluation of gene set-based PRS for CAD as a function of the number of cell state marker genes selected. The indicated number of top marker genes were retained (up to the maximum available). Upper panel: PRS predictive power (PRS.R2 = full model R2 – null model R2; the difference in R2 for a model including PRS and covariates, compared to a model with only covariates). Bottom panel: permutation-based significance test comparing the performance to identically-clumped SNP sets from background regions (genes + flanks), as implemented by PRSet[4].

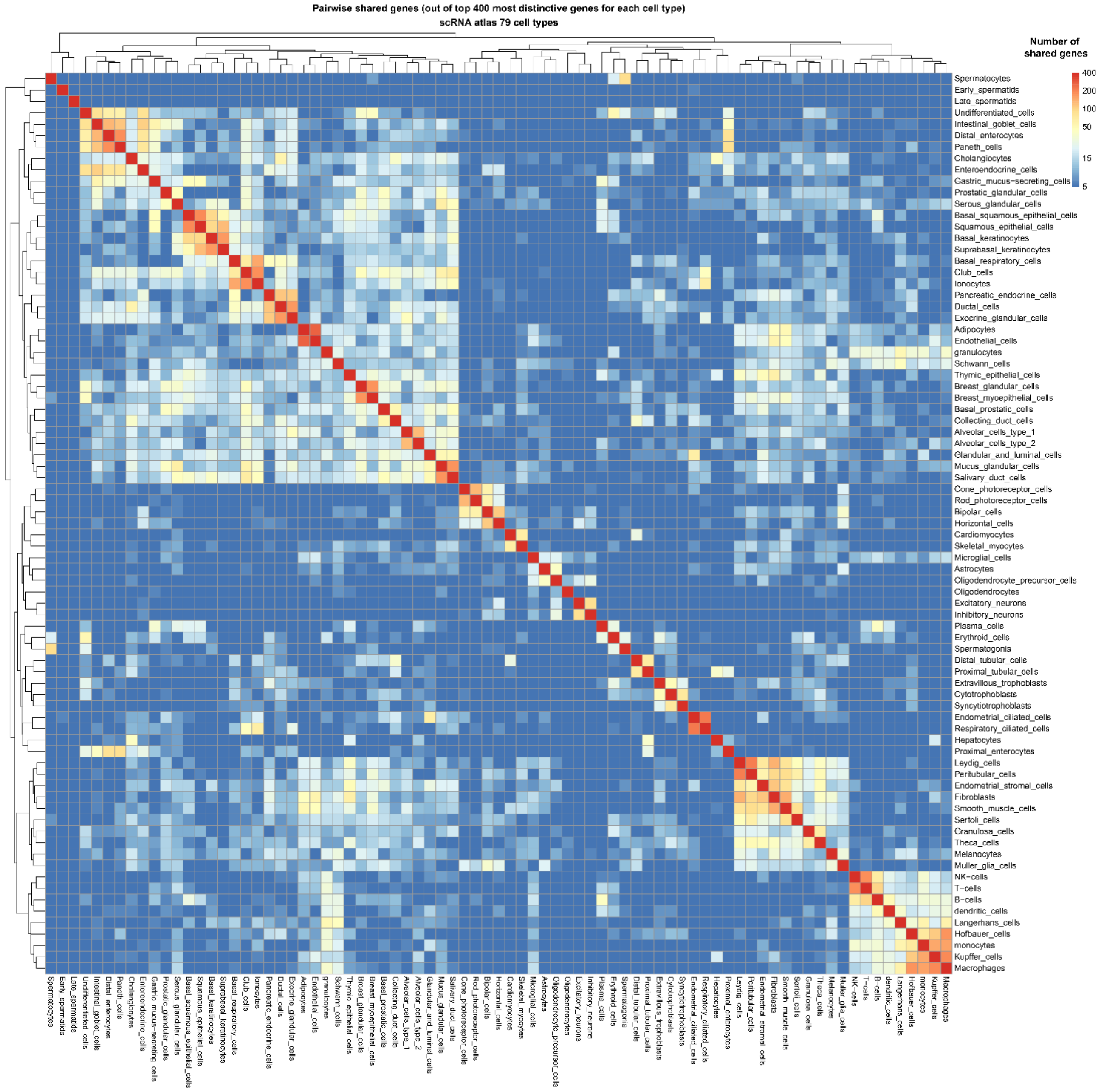

Figure S23. Number of pairwise shared marker genes for 79 human cell types. Marker genes were selected as described in Methods using gene expression profiles compiled by the Protein Atlas from 30 scRNA-Seq datasets. For each cell type, the top 400 marker genes were used. Color scale shows the number of shared genes.

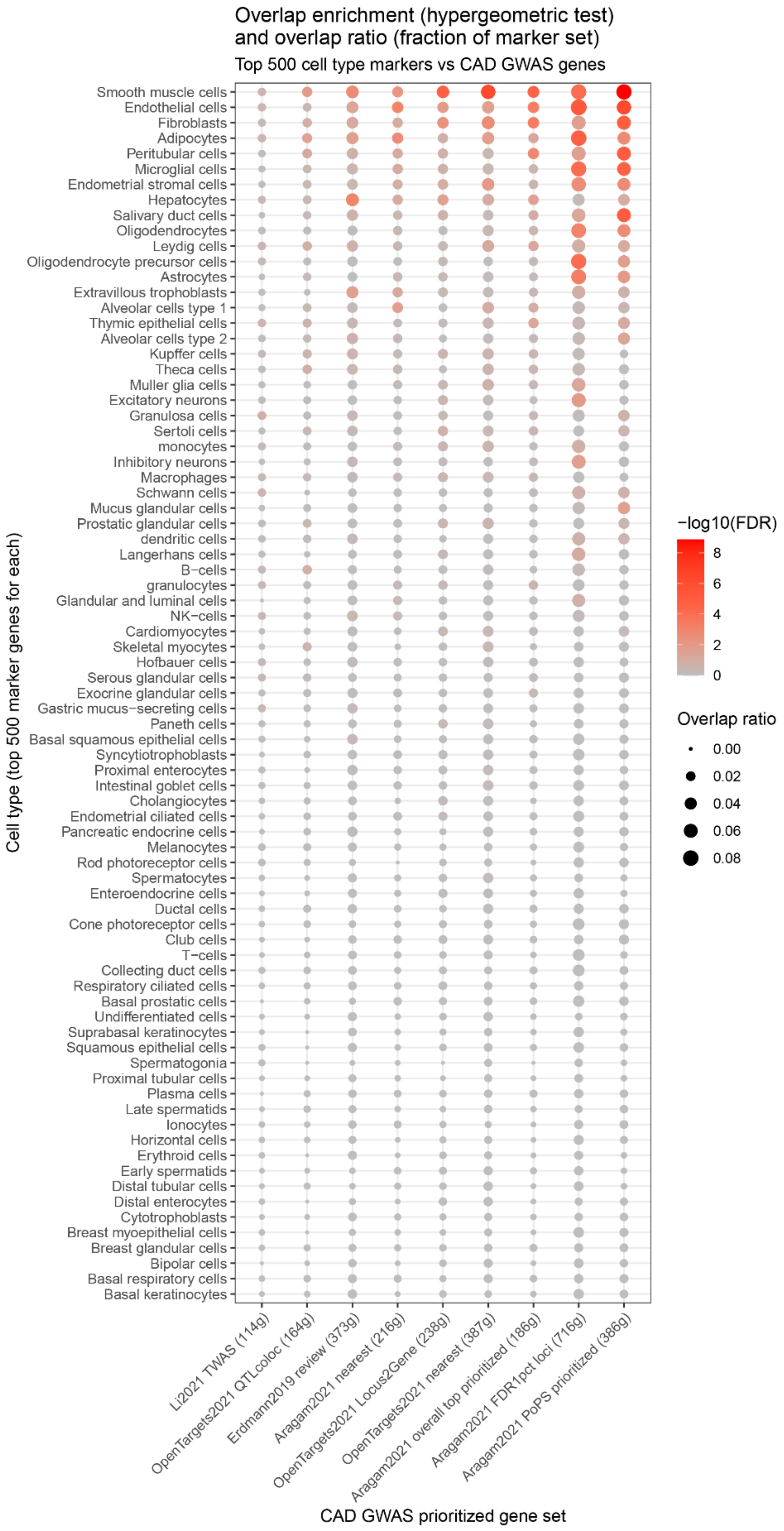

Figure S24. Enrichment of CAD GWAS prioritized genes among the top 500 cell type markers of 79 human cell types. Markers were selected as described in Methods. Overlap enrichment was tested by hypergeometric test using 18043 genes as the background. Column and row order is by average -log10(FDR). The dot size indicates the ratio of CAD GWAS genes within the marker gene set.

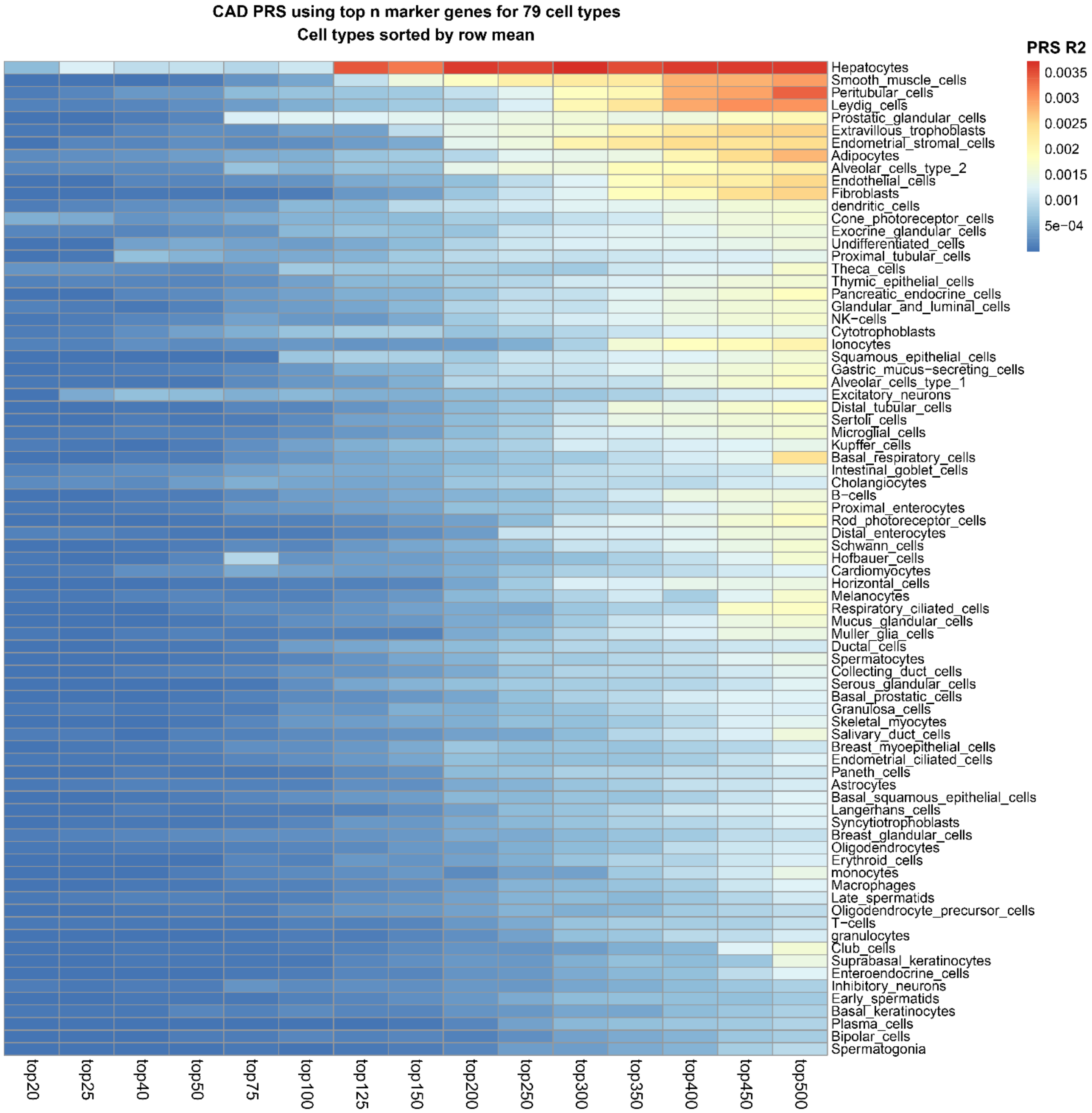

Figure S25. Gene set-based PRS for CAD using marker gene sets for 79 human cell types. The indicated number of top marker genes was selected for each cell type and used to define a PRS using the gene bodies + flanks. Color scale shows the PRS.R2 (full model R2 – null model R2, i.e., the difference in R2 for a model with PRS and covariates, compared to a model with only covariates). Cell types are sorted according to row mean.

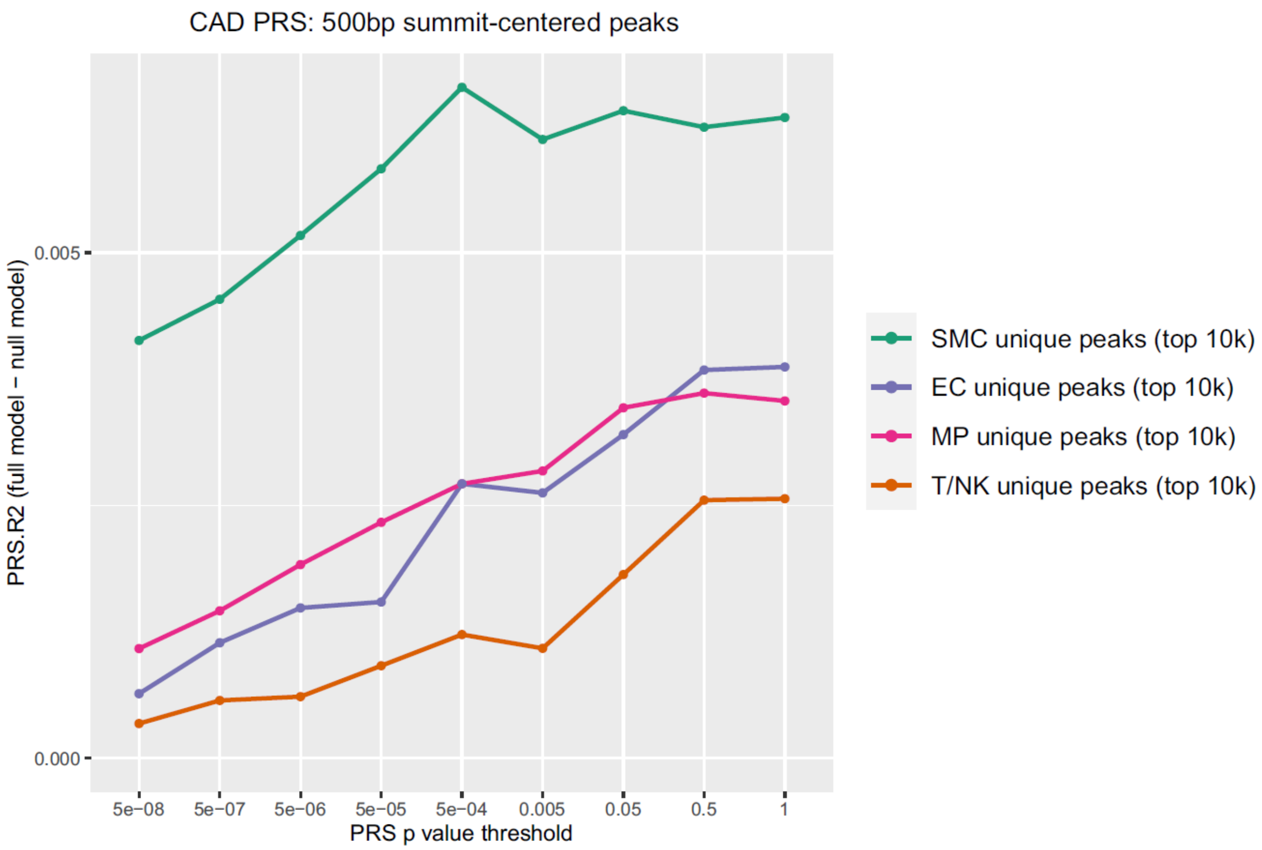

Figure S26. Proportion of variance of CAD explained by polygenic risk score (PRS) calculated using to 10,000 strongest cell type-specific peaks of each cell type. Peaks were ranked by ATAC signal score as calculated by the peak caller (MACS2)[5]. Peaks were each 500 bp in length. Thus, the fraction of genome included in the search space based for each cell type is equal-sized and non-overlapping between the cell types.

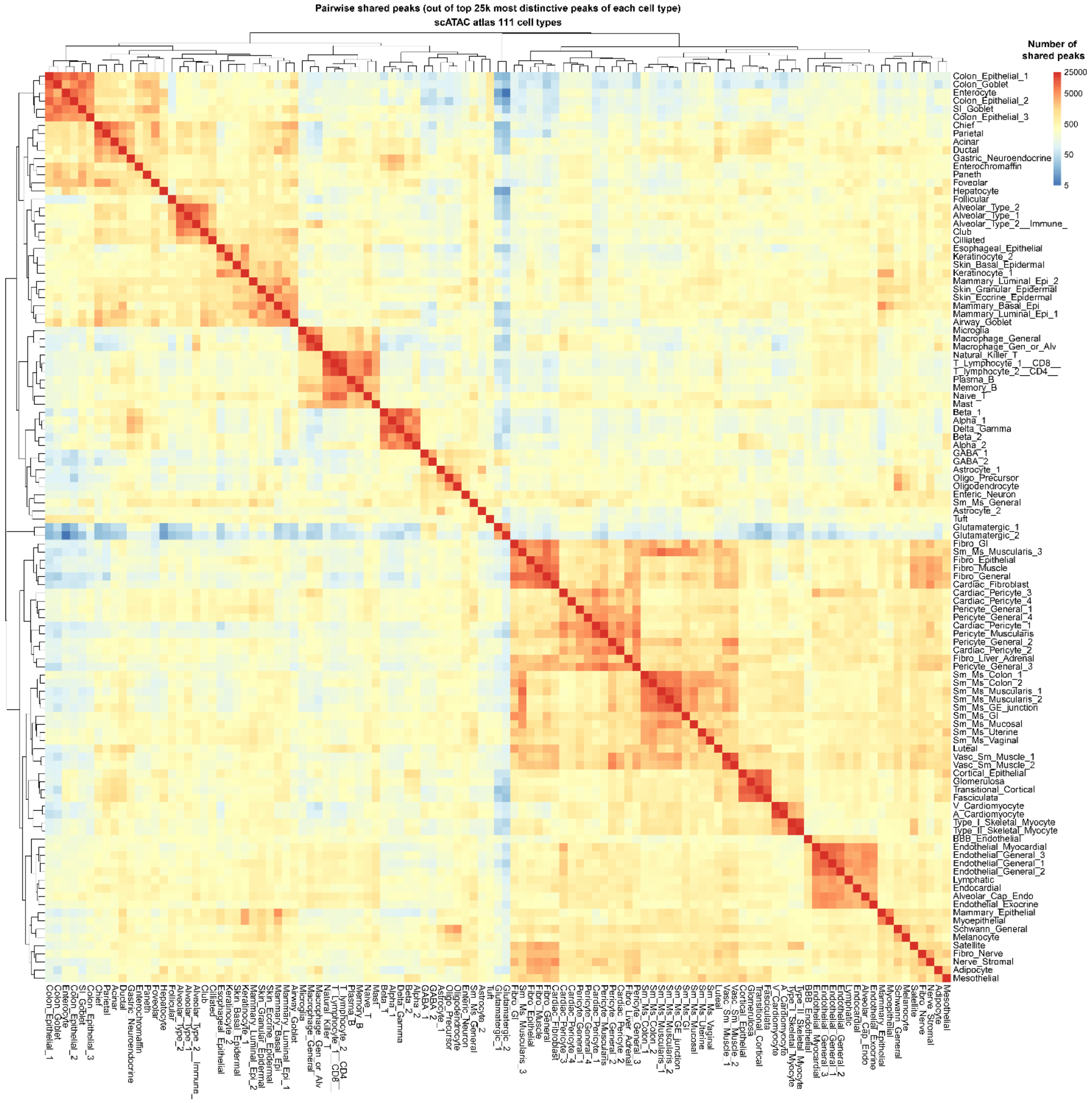

Figure S27. Number of pairwise shared scATAC-Seq peaks among top 25,000 most distinctive peaks of 111 human cell types. All adult cell types of the human scATAC-Seq atlas[6] were used. All peaks were of equal width. The cell type peak accessibility matrix was z-scored per peak, and the top 25,000 peaks were selected per cell type to obtain the most distinctive peaks. Color scale shows the number of shared peaks between pairs of cell types.

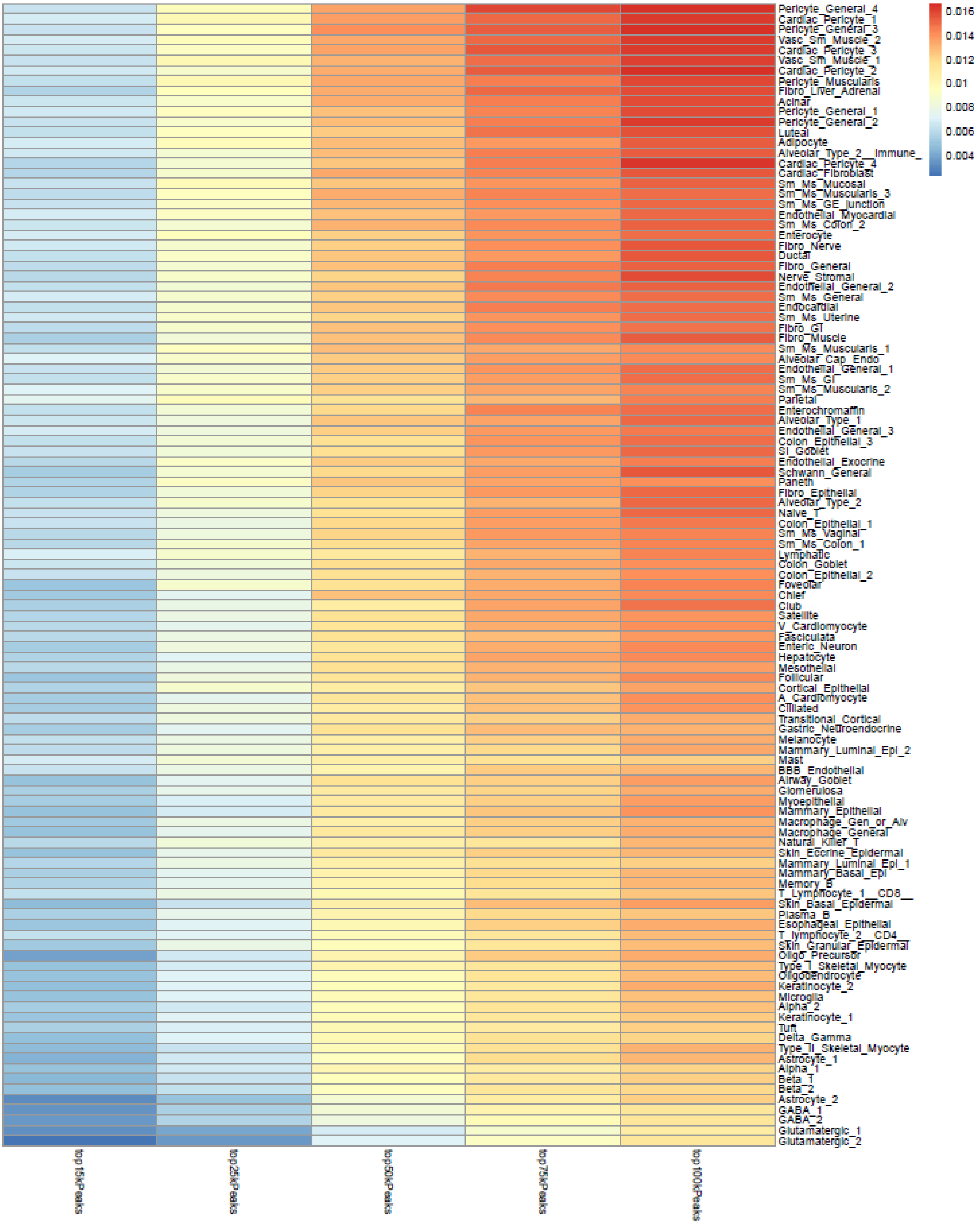

Figure S28. Evaluation of CAD PRS strength among the 111 human cell types of the adult single-cell atlas of chromatin accessibility[6]. PRS R2 (PRS strength) values are shown. For each cell type, the indicated number (either 100,000; 75,000; 50,000; 25,000 or 15,000) of strongest peaks were selected for PRS calculation. All peaks were of equal width.

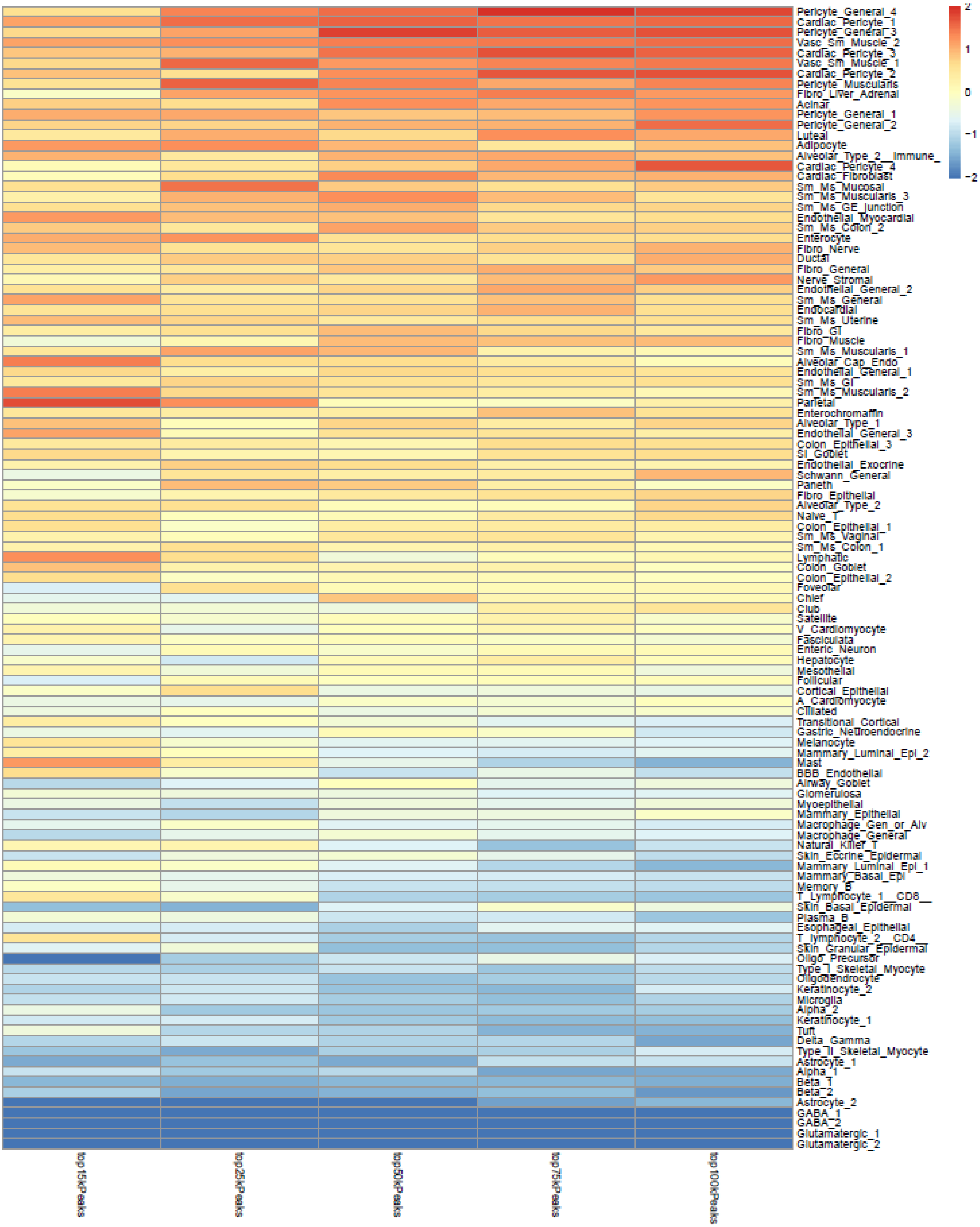

Figure S29. Evaluation of CAD PRS strength among the 111 human cell types of the adult single-cell atlas of chromatin accessibility[6]. The results are as in Figure S28, except the column z-score of the PRS R2 is shown.

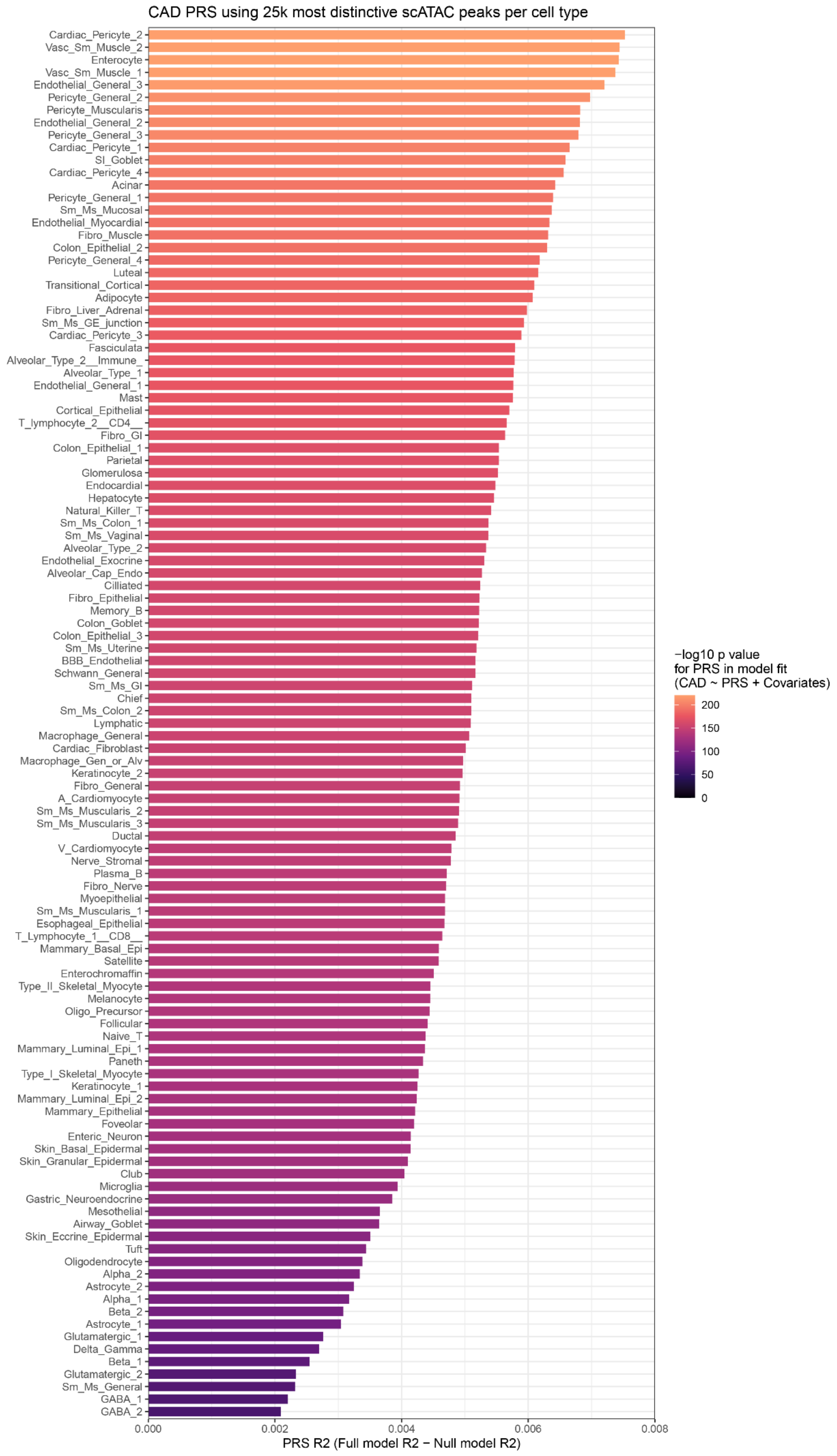
CAD PRS using 25k most distinctive scATAC peaks per cell type
Cardiac_Pericyte_2
Vasc_Sm_Muscle_2
Enterocyte
Vasc_Sm_Muscle_1
Endothelial_General_3
Pericyte_General_2
Pericyte_Muscularis
Endothelial_General_2
Pericyte_General_3
Cardiac_Pericyte_1
SI_Goblet
Cardiac_Pericyte_4
Acinar
Pericyte_General_1
Sm_Ms_Mucosal
Endothelial_Myocardial
Fibro_Muscle
Colon_Epithelial_2
Pericyte_General_4
Luteal
Transitional_Cortical
Adipocyte
Fibro_Liver_Adrenal
Sm_Ms_GE_junction
Cardiac_Pericyte_3
Fasciculata
Alveolar_Type_2__Immune_
Alveolar_Type_1
Endothelial_General_1
Mast
Cortical_Epithelial
T_lymphocyte_2__CD4__
Fibro_GI
Colon_Epithelial_1
Parietal
Glomerulosa
Endocardial
Hepatocyte
Natural_Killer_T
Sm_Ms_Colon_1
Sm_Ms_Vaginal
Alveolar_Type_2
Endothelial_Exocrine
Alveolar_Cap_Endo
Cilliated
Fibro_Epithelial
Memory_B
Colon_Goblet
Colon_Epithelial_3
Sm_Ms_Uterine
BBB_Endothelial
Schwann_General
Sm_Ms_GI
Chief
Sm_Ms_Colon_2
Lymphatic
Macrophage_General
Cardiac_Fibroblast
Macrophage_Gen_or_Alv
Keratinocyte_2
Fibro_General
A_Cardiomyocyte
Sm_Ms_Muscularis_2
Sm_Ms_Muscularis_3
Ductal
V_Cardiomyocyte
Nerve_Stromal
Plasma_B
Fibro_Nerve
Myoepithelial
Sm_Ms_Muscularis_1
Esophageal_Epithelial
T_Lymphocyte_1__CD8__
Mammary_Basal_Epi
Satellite
Enterochromaffin
Type_II_Skeletal_Myocyte
Melanocyte
Oligo_Precursor
Follicular
Naive_T
Mammary_Luminal_Epi_1
Paneth
Type_I_Skeletal_Myocyte
Keratinocyte_1
Mammary_Luminal_Epi_2
Mammary_Epithelial
Foveolar
Enteric_Neuron
Skin_Basal_Epidermal
Skin_Granular_Epidermal
Club
Microglia
Gastric_Neuroendocrine
Mesothelial
Airway_Goblet
Skin_Eccrine_Epidermal
Tuft
Oligodendrocyte
Alpha_2
Astrocyte_2
Alpha_1
Beta_2
Astrocyte_1
Glutamatergic_1
Delta_Gamma
Beta_1
Glutamatergic_2
Sm_Ms_General
GABA_1
GABA_2
0.000
0.002
0.004
0.006
0.008
PRS R2 (Full model R2 − Null model R2)
−log10 p value
for PRS in model fit
(CAD ~ PRS + Covariates)
200
150
100
50
0

Figure S30. Performance of CAD PRS derived using 25,000 most cell type-specific peaks for each of 111 cell types in the adult scATAC atlas[6]. All peaks were of equal width. The cell type peak accessibility matrix was z-scored per peak, and the highest scoring peaks were selected per cell type to obtain the most distinctive peaks.

Supplemental Table Legends

Table S1. Markers of the major cell types identified by supervised clustering.

Table S2. Markers of the 12 atherosclerosis-associated cell states.

Table S3. Differentially expressed genes identified in bulk RNA-Seq experiment. Genes are sorted by effect direction followed by statistical significance.

Table S4. Complete gene ontology listing of the cell state marker genes used to generate Figure 3C.

Table S5. Cell type marker genes for 79 human cell types based on scRNA-Seq expression profiles (https://www.proteinatlas.org/about/download). For each cell type, top 500 genes are ranked in descending order of importance.

Supplemental Methods

Differentially abundant cellular neighborhood analysis for scRNA-Seq

Milo version 1.7.1[1] was used for testing differential neighborhood abundance with a k-nearest neighbor graph calculated using $k = 20$ and the first 30 dimensions from PCA from Seurat.

Mouse bulk RNA-Seq differential expression and cell type decomposition

For differential expression analysis between experiment groups, lowly expressed genes were first removed using the EdgeR (version 3.24.3)[7] function filterByExpr (minimum count per sample 15, minimum total count 50). The remaining 15559 genes were used in differential expression analysis with DESeq2 (version 1.22.2)[8]. FDR < 0.05 was considered significant.

Cell type proportions in mouse aorta bulk tissue RNA-Seq profiles were estimated using the CIBERSORTx web tool (access date 2019-10-17)[9]. Aorta scRNA-Seq cells from the current study were used as the reference transcriptome profiles for bulk RNA expression profile decomposition. For generating the cell type reference signatures, single cells were annotated to the cell type level, 7 most abundant cell types were retained, cells randomly subsampled to a maximum of 1,200 cells per cell type, and genes expressed in >5 cells were retained (total 14,632 genes).

Single cell trajectory analysis

Monocle (version 2.8.0)[10] was used for pseudotime trajectory modeling following the author's recommendations. To model the transition from contractile (*Myh11*-expressing) SMC-s to disease-increased SMC cell states, cells from the 3-month HFD library were used to avoid the need for batch correction. Contractile SMC-s were randomly subsampled to 500

cells to approximately match the number of cells in the disease-increased populations. The gene expression count preprocessing and automated cell clustering was done according to the Monocle 2 tutorial, followed by cluster marker gene detection using the Monocle differentialGeneTest function (model: '~Cluster'). The 1000 most significant genes by *p* value were used for trajectory construction using default parameters. To model the *in vitro* SMC IL-1β response trajectory, G1-phase cells (identified using Seurat cell cycle scoring with default parameters) from control and IL-1β treatment were used. Monocle 2 default processing was used, as above. As the trajectory ordering genes, all differentially expressed genes comparing treatment and control sample cells were used (Wilcoxon test, FDR < 0.05). To evaluate concordance between the *in vivo* SMC dedifferentiation trajectory and the *in vitro* SMC IL-1β response trajectory, gene set activity scores (function AddModuleScore from Seurat) were calculated for each cell and shown on cells positioned in pseudotime space. The scores were based on the 50 most significant genes by *p* value of either the *in vivo* cell state gene signature or the *in vitro* treatment differential expression.

<u>Single-cell analysis of cultured smooth muscle cells</u>

Mouse immortalized aortic smooth muscle cells (MOVAS; ATCC cell line CRL-2797) were cultured in DMEM supplemented with 10% FBS, 100 U/ml penicillin, 100 µg/ml streptomycin and 200 µg/ml geneticin in a 37 °C incubator with 5% CO_2. Cells were cultured in 12-well plates to approximately 70% confluency. Prior to experimental treatments, cells were incubated for 24 h in serum-starvation medium (DMEM supplemented with 0.2% BSA). Subsequently, the medium was replaced with serum-starvation medium supplemented with recombinant interleukin-1β (IL-1β; Sino Biological #10139HNAE) at 25 or 50 ng/ml and incubated for a further 24 or 48 h. The different IL-1β treatments were timed to end concurrently and, after trypsinization, the cells were pooled in equal counts to obtain a mixture of cells at different phases of the IL-1β response. As a control treatment, 24 h serum-

starved cells were placed in fresh serum starvation medium for a further 48 h.

The Chromium Single Cell 3' Kit (v3 Chemistry; 10xGenomics) was used to prepare scRNA-Seq libraries for control and IL-1β treated cells in separate lanes. Paired-end high-throughput sequencing was carried out on an Illumina NextSeq 550 instrument (Read 1: 28 bp, Read 2: 91 bp). Sequencing reads were processed using the Cell Ranger pipeline (version 3.0.2; 10xGenomics) and the mm10 reference transcriptome package (version 3.0.0).

Cell state gene signature activity in human plaque scRNA-seq cells and scRNA-Seq of alternative models of mouse atherosclerosis

Human coronary atherosclerosis scRNA-Seq datasets published by Wirka et al[3] (GEO: GSE131778) and mouse atherosclerosis scRNA-Seq datasets of *Apoe*$^{-/-}$ and *Ldlr*$^{-/-}$ mouse models published by Pan et al[2] (GEO: GSE155513) were analyzed to identify cells that have activated the cell state gene programs using the gene signature activity calculation implemented in the Seurat function AddModuleScore. Briefly, genes are binned based on average log expression level across samples, and, in each sample, a bin background level (calculated from random control genes in the same bin) is subtracted from the levels of the test genes.

Chromatin accessibility and gene expression by cell type for human tissues throughout the body

The adult human scATAC-Seq atlas[6] data matrix of chromatin accessibility of 111 cell types in a common peak set of approximately 890000 equal-width peaks was downloaded from Mendeley Data (DOI: 10.17632/yv4fzv6cnm.4) and the peak coordinates were lifted over from hg38 to hg19. To obtain peak profiles representative of each cell type, peaks were ranked within a cell type by accessibility signal strength and the 15000, 25000, 50000, 75000

or 100000 strongest peaks were selected.

Gene expression (TPM) profiles for 79 human cell types across the body, compiled from 30 scRNA-Seq datasets, were obtained from the Protein Atlas[11] (https://www.proteinatlas.org/about/download; access date 2023-02-10). Out of the initial 20090 genes, genes expressed at >5 TPM in at least one cell type were retained, resulting in 18043 genes. To select cell type marker genes (most distinctive genes), gene expression was first transformed into z-score per gene (gene expression relative to the variation of that gene), and then top n genes were selected within each cell type.

Immunohistochemistry

Tissue sections were blocked with 10% normal goat serum, and incubated with following primary antibodies: recombinant anti-VCAM1 antibody (ab134047, Abcam, Cambridge, UK; dilution 1:100), palladin polyclonal antibody (10853-1-AP, Proteintech, Manchester, UK; dilution 1:100), LRG1 polyclonal antibody (PA5-76287, Thermo Fisher Scientific, Waltham, MA; dilution 1:200), and monoclonal mouse anti-actin, α-smooth muscle-Cy3 (C6198, Sigma-Aldrich, St. Louis, MO; dilution 1:50 or 1:100). Biotinylated goat anti-rabbit IgG (BA-1000, Vector Laboratories, Burlingame, CA) secondary antibody and fluorescein Avidin DCS (A-2011, Vector Laboratories) were used. Nuclei were stained with DAPI (H-1200, Vector Laboratories). Imaging was performed by Zeiss LSM800 Airyscan confocal microscope with 405/488/555 nm diode lasers together with the appropriate emission filters (Plan-Apochromat 20×/0.8 objective, 1024 × 1024 and 2048 × 2048 frame sizes).

Molecular Cartography

Mice were euthanized and a full-body perfusion was executed using PBS (Gibco). Aortas and hearts were extracted on ice. The tissues were embedded in (VWR Chemicals) and then snap frozen fresh in isopentane (Fisher Scientific). The isopentane was chilled on dry

ice for 30 minutes prior to tissue freezing. The embedded tissues were kept in -70°C until sectioning. The embedded tissue samples and the Resolve Biosciences Molecular CartographyTM slides were put in the cryostat (Leica Biosystems CM1950 Cryostat, temperature at -20°C) 30 minutes prior to cryosectioning. 10 µm sections were cut and placed on the capture areas of Resolve Biosciences Molecular CartographyTM slides. The slides were packed in dry ice and sent to Resolve Biosciences for further processing. Upon arrival, tissue sections were thawed for 30 min at 37°C to improve adhesion and were fixed with 4% v/v Formaldehyde (Sigma-Aldrich F8775) in 1x PBS for 30 min at 4 °C. After fixation, sections were washed three times in 1x PBS for one min, followed by one min washes in 70% Ethanol, isopropanol, 100% Ethanol and 70% Ethanol at room temperature. Fixed samples were used for Molecular Cartography (100-plex combinatorial single molecule fluorescence in-situ hybridization) according to the manufacturer's instructions, starting with the aspiration of ethanol and incubation in Trueblack for 5 min, followed by buffer DST1, tissue priming and hybridization. Briefly, tissues were primed for 30 min at 37°C followed by 24h hybridization of all probes specific for the target genes. After the hybridizations step, samples were washed to remove excess probes and fluorescently tagged in a two-step color development process. Regions of interest were imaged as described below and fluorescent signals removed during decolorization. Color development, imaging and decolorization were repeated for multiple cycles to build a unique combinatorial code for every target gene that was derived from raw images as described below.

The probes for selected genes were designed using Resolve's proprietary design algorithm. Briefly, the probe-design was performed at the gene-level. For every targeted gene all full-length protein coding transcript sequences from the ENSEMBL database were used as design targets if the isoform had the GENCODE annotation tag 'basic'[12; 13]. To speed up the process, the calculation of computationally expensive parts, especially the off-

target searches, the selection of probe sequences was not performed randomly, but limited to sequences with high success rates. To filter highly repetitive regions, the abundance of k-mers was obtained from the background transcriptome using Jellyfish[14]. Every target sequence was scanned once for all k-mers, and those regions with rare k-mers were preferred as seeds for full probe design. A probe candidate was generated by extending a seed sequence until a certain target stability was reached. A set of simple rules was applied to discard sequences that were found experimentally to cause problems. After these fast screens, every kept probe candidate was mapped to the background transcriptome using ThermonucleotideBLAST[15] and probes with stable off-target hits were discarded. Specific probes were then scored based on the number of on-target matches (isoforms), which were weighted by their associated APPRIS level[16], favoring principal isoforms over others. A bonus was added if the binding-site was inside the protein-coding region. From the pool of accepted probes, the final set was composed by greedily picking the highest scoring probes. The following table highlights the gene names and Catalogue numbers for the specific probes designed by Resolve BioSciences with gene list name KG719.

Samples were imaged on a Zeiss Celldiscoverer 7, using the 50x Plan Apochromat water immersion objective with an NA of 1.2 and the 0.5x magnification changer, resulting in a 25x final magnification. Standard CD7 LED excitation light source, filters, and dichroic mirrors were used together with customized emission filters optimized for detecting specific signals. Excitation time per image was 1000 ms for each channel (DAPI was 20 ms). A z-stack was taken at each region with a distance per z-slice according to the Nyquist-Shannon sampling theorem. The custom CD7 CMOS camera (Zeiss Axiocam Mono 712, 3.45 µm pixel size) was used. For each region, a z-stack per fluorescent color (two colors) was imaged per imaging round. A total of 8 imaging rounds were done for each position, resulting in 16 z-stacks per region. The completely automated imaging process per round (including

water immersion generation and precise relocation of regions to image in all three dimensions) was realized by a custom python script using the scripting API of the Zeiss ZEN software (Open application development).

The algorithms for spot segmentation were written in Java and are based on the ImageJ library functionalities. Only the iterative closest point algorithm is written in C++ based on the libpointmatcher library (https://github.com/ethz-asl/libpointmatcher). As a first step all images were corrected for background fluorescence. A target value for the allowed number of maxima was determined based upon the area of the slice in μm^2 multiplied by the factor 0.5. This factor was empirically optimized. The brightest maxima per plane were determined, based upon an empirically optimized threshold. The number and location of the respective maxima was stored. This procedure was done for every image slice independently. Maxima that did not have a neighboring maximum in an adjacent slice (called z-group) were excluded. The resulting maxima list was further filtered in an iterative loop by adjusting the allowed thresholds for (Babs-Bback) and (Bperi-Bback) to reach a feature target value (Babs: absolute brightness, Bback: local background, Bperi: background of periphery within 1 pixel). This feature target values were based upon the volume of the 3D-image. Only maxima still in a z-group of at least 2 after filtering were passing the filter step. Each z-group was counted as one hit. The members of the z-groups with the highest absolute brightness were used as features and written to a file. They resemble a 3D-point cloud. Final signal segmentation and decoding: To align the raw data images from different imaging rounds, images had to be corrected. To do so the extracted feature point clouds were used to find the transformation matrices. For this purpose, an iterative closest point cloud algorithm was used to minimize the error between two point-clouds. The point clouds of each round were aligned to the point cloud of round one (reference point cloud). The corresponding point clouds were stored for downstream processes. Based upon the

transformation matrices the corresponding images were processed by a rigid transformation using trilinear interpolation. The aligned images were used to create a profile for each pixel consisting of 16 values (16 images from two color channels in 8 imaging rounds). The pixel profiles were filtered for variance from zero normalized by total brightness of all pixels in the profile. Matched pixel profiles with the highest score were assigned as an ID to the pixel. Pixels with neighbors having the same ID were grouped. The pixel groups were filtered by group size, number of direct adjacent pixels in group, number of dimensions with size of two pixels. The local 3D-maxima of the groups were determined as potential final transcript locations. Maxima were filtered by number of maxima in the raw data images where a maximum was expected. Remaining maxima were further evaluated by the fit to the corresponding code. The remaining maxima were written to the results file and considered to resemble transcripts of the corresponding gene. The ratio of signals matching to codes used in the experiment and signals matching to codes not used in the experiment were used as estimation for specificity (false positives). Final image analysis was performed in ImageJ using the Polylux tool plugin from Resolve BioSciences to examine specific Molecular Cartography signals.

Supplemental References

1. Dann, E., Henderson, N.C., Teichmann, S.A., Morgan, M.D., and Marioni, J.C. (2022). Differential abundance testing on single-cell data using k-nearest neighbor graphs. Nat Biotechnol 40, 245-253.
2. Pan, H., Xue, C., Auerbach, B.J., Fan, J., Bashore, A.C., Cui, J., Yang, D.Y., Trignano, S.B., Liu, W., Shi, J., et al. (2020). Single-Cell Genomics Reveals a Novel Cell State During Smooth Muscle Cell Phenotypic Switching and Potential Therapeutic Targets for Atherosclerosis in Mouse and Human. Circulation 142, 2060-2075.
3. Wirka, R.C., Wagh, D., Paik, D.T., Pjanic, M., Nguyen, T., Miller, C.L., Kundu, R., Nagao, M., Coller, J., Koyano, T.K., et al. (2019). Atheroprotective roles of smooth muscle cell phenotypic modulation and the TCF21 disease gene as revealed by single-cell analysis. Nat Med 25, 1280-1289.
4. Choi, S.W., Garcia-Gonzalez, J., Ruan, Y., Wu, H.M., Porras, C., Johnson, J., Bipolar Disorder Working group of the Psychiatric Genomics, C., Hoggart, C.J., and O'Reilly, P.F. (2023). PRSet: Pathway-based polygenic risk score analyses and software. PLoS Genet 19, e1010624.
5. Zhang, Y., Liu, T., Meyer, C.A., Eeckhoute, J., Johnson, D.S., Bernstein, B.E., Nusbaum, C., Myers, R.M., Brown, M., Li, W., et al. (2008). Model-based analysis of ChIP-Seq (MACS). Genome Biol 9, R137.
6. Zhang, K., Hocker, J.D., Miller, M., Hou, X., Chiou, J., Poirion, O.B., Qiu, Y., Li, Y.E., Gaulton, K.J., Wang, A., et al. (2021). A single-cell atlas of chromatin accessibility in the human genome. Cell 184, 5985-6001 e5919.
7. Robinson, M.D., McCarthy, D.J., and Smyth, G.K. (2010). edgeR: a Bioconductor package for differential expression analysis of digital gene expression data. Bioinformatics 26, 139-140.
8. Love, M.I., Huber, W., and Anders, S. (2014). Moderated estimation of fold change and dispersion for RNA-seq data with DESeq2. Genome Biol 15, 550.
9. Newman, A.M., Steen, C.B., Liu, C.L., Gentles, A.J., Chaudhuri, A.A., Scherer, F., Khodadoust, M.S., Esfahani, M.S., Luca, B.A., Steiner, D., et al. (2019). Determining cell type abundance and expression from bulk tissues with digital cytometry. Nat Biotechnol 37, 773-782.
10. Qiu, X., Mao, Q., Tang, Y., Wang, L., Chawla, R., Pliner, H.A., and Trapnell, C. (2017). Reversed graph embedding resolves complex single-cell trajectories. Nat Methods 14, 979-982.
11. Karlsson, M., Zhang, C., Mear, L., Zhong, W., Digre, A., Katona, B., Sjostedt, E., Butler, L., Odeberg, J., Dusart, P., et al. (2021). A single-cell type transcriptomics map of human tissues. Sci Adv 7.
12. Frankish, A., Carbonell-Sala, S., Diekhans, M., Jungreis, I., Loveland, J.E., Mudge, J.M., Sisu, C., Wright, J.C., Arnan, C., Barnes, I., et al. (2022). GENCODE: reference annotation for the human and mouse genomes in 2023. Nucleic Acids Res.
13. Yates, A.D., Achuthan, P., Akanni, W., Allen, J., Allen, J., Alvarez-Jarreta, J., Amode, M.R., Armean, I.M., Azov, A.G., Bennett, R., et al. (2020). Ensembl 2020. Nucleic Acids Res 48, D682-D688.
14. Marcais, G., and Kingsford, C. (2011). A fast, lock-free approach for efficient parallel counting of occurrences of k-mers. Bioinformatics 27, 764-770.
15. Gans, J.D., and Wolinsky, M. (2008). Improved assay-dependent searching of nucleic acid sequence databases. Nucleic Acids Res 36, e74.
16. Rodriguez, J.M., Rodriguez-Rivas, J., Di Domenico, T., Vazquez, J., Valencia, A., and Tress, M.L. (2018). APPRIS 2017: principal isoforms for multiple gene sets. Nucleic Acids Res 46, D213-D217.
