# Supplementary material for: Dissecting the polygenic basis of atherosclerosis via disease-associated cell state signatures
Source: Am J Hum Genet. 2023 Apr 14;110(5):722–40. doi: 10.1016/j.ajhg.2023.03.013 (PMC10183377; doi:10.1016/j.ajhg.2023.03.013)
Supplement: Document S2. Article plus supplemental information [file mmc3.pdf]

ARTICLE

Dissecting the polygenic basis of atherosclerosis via disease-associated cell state signatures

Graphical abstract

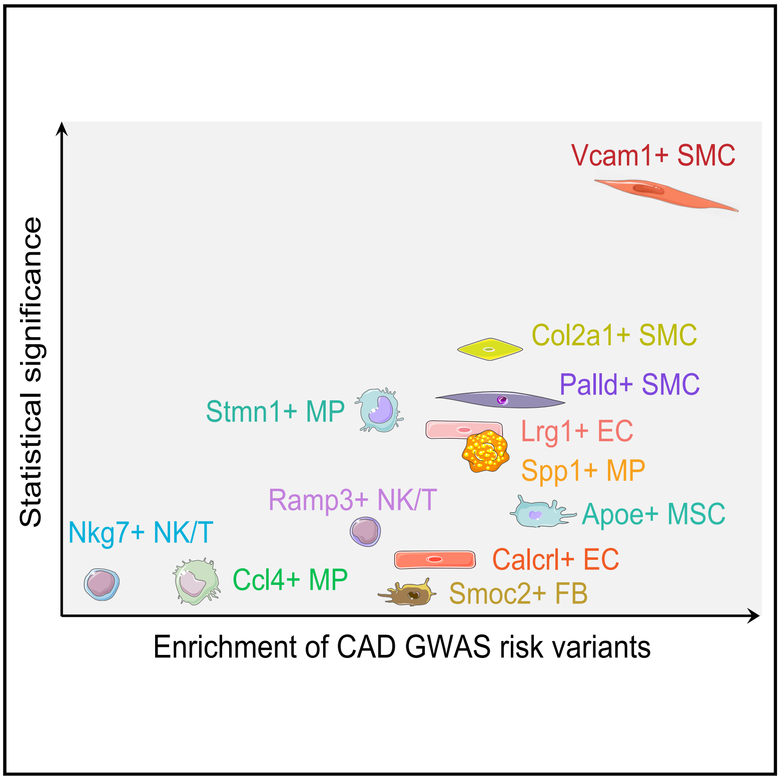

Authors

Tiit Örd, Tapio Lönnberg, Valtteri Nurminen, ..., Johanna P. Laakkonen, Terho Lehtimäki, Minna U. Kaikkonen

Correspondence

tiit.ord@uef.fi (T.Ö.),
minna.kaikkonen@uef.fi (M.U.K.)

Using single-cell RNA sequencing, researchers identified 12 atherosclerosis-associated cell states and found that Vcam1+ smooth muscle cell state contributed the most to the genetic heritability of coronary artery disease. A hybrid polygenic risk score (PRS) that considers cell-type-specific regulatory elements demonstrated improved performance over a classical PRS.

Örd et al., 2023, The American Journal of Human Genetics *110*, 722–740
May 4, 2023 © 2023 The Author(s).
https://doi.org/10.1016/j.ajhg.2023.03.013

ARTICLE

Dissecting the polygenic basis of atherosclerosis via disease-associated cell state signatures

Tiit Örd,[1,*] Tapio Lönnberg,[2,3] Valtteri Nurminen,[1] Aarthi Ravindran,[1] Henri Niskanen,[1] Miika Kiema,[1] Kadri Õunap,[1] Maleeha Maria,[1] Pierre R. Moreau,[1] Pashupati P. Mishra,[4] Senthil Palani,[5] Jenni Virta,[5] Heidi Liljenbäck,[5,6] Einari Aavik,[1] Anne Roivainen,[5,6,7] Seppo Ylä-Herttuala,[1] Johanna P. Laakkonen,[1] Terho Lehtimäki,[4] and Minna U. Kaikkonen[1,*]

Summary

Coronary artery disease (CAD) is a pandemic disease where up to half of the risk is explained by genetic factors. Advanced insights into the genetic basis of CAD require deeper understanding of the contributions of different cell types, molecular pathways, and genes to disease heritability. Here, we investigate the biological diversity of atherosclerosis-associated cell states and interrogate their contribution to the genetic risk of CAD by using single-cell and bulk RNA sequencing (RNA-seq) of mouse and human lesions. We identified 12 disease-associated cell states that we characterized further by gene set functional profiling, ligand-receptor prediction, and transcription factor inference. Importantly, Vcam1+ smooth muscle cell state genes contributed most to SNP-based heritability of CAD. In line with this, genetic variants near smooth muscle cell state genes and regulatory elements explained the largest fraction of CAD-risk variance between individuals. Using this information for variant prioritization, we derived a hybrid polygenic risk score (PRS) that demonstrated improved performance over a classical PRS. Our results provide insights into the biological mechanisms associated with CAD risk, which could make a promising contribution to precision medicine and tailored therapeutic interventions in the future.

Introduction

Cell cycle stages and cell types are well understood dimensions of cellular heterogeneity that are routinely applied to distinguish cell populations. Still, within a population of relatively homogeneous cells, significant variability in specific responses to an identical stimulus can be detected, also known as cell states. Efforts to determine the extent of such cell-to-cell heterogeneity in pathological conditions have been revolutionized by the emergence of single-cell RNA sequencing (scRNA-seq)-based studies. To this end, cardiovascular research has witnessed an explosion of studies describing an unprecedented degree of cell diversity in atherosclerotic lesions. scRNA-seq and cytometry by time of flight (CyTOF) methods have identified at least 19 types of leukocytes, consisting of 3–5 different macrophage subsets, 3–5 T cell subsets, two B cell subsets, two types of monocytes, two types of dendritic cells, one NK cell subset, neutrophils, and eosinophils in atherosclerotic mouse aortas.[1] In addition, seven clusters of smooth muscle cells (SMCs) have been identified, demonstrating dedifferentiation of contractile SMCs into intermediate cells, termed "SEM" cells (stem cell, endothelial cell, monocyte) and further into fibromyocytes/fibrochondrocyte-like cells and to an osteogenic phenotype.[2–6] Additionally, eight endothelial cell (EC) subpopulations have been identified as a result of diabetogenic high-fat diet or disturbed flow where proatherogenic conditions induced a dramatic transition of ECs into progenitor, proinflammatory, mesenchymal (EndMT), and immune cell-like phenotypes.[7,8] Still, consistent definition and comparison of altered cell states in atherosclerosis is missing, complicating the understanding of mechanisms and pathways that contribute to pathological cell states.

An individual's risk of atherosclerotic coronary artery disease (CAD) is determined by an interplay of environmental and genetic factors. The recent genome-wide association studies (GWASs) for CAD with over 1 million participants have identified over 300 risk loci.[9,10] Despite extensive progress in statistical and experimental tools used to link regions of the genome to disease risk, it remains challenging to identify the causal genes underlying genetic associations and the cell types through which the effect is mediated. We have recently demonstrated that cell-type-specific chromatin accessibility and gene expression provide a means for predicting the cell type of action for CAD loci.[11] To this end, we demonstrated that the *cis*-regulatory elements active in SMCs and ECs show the highest enrichment of GWAS SNPs for CAD and blood pressure among the lesional cell types. However, the contributions of specific disease-associated cell states or gene signatures to the disease risk and to CAD heritability remain unknown.

[1]A.I. Virtanen Institute for Molecular Sciences, University of Eastern Finland, 70211 Kuopio, Finland; [2]Turku Bioscience Centre, University of Turku and Åbo Akademi University, 20520 Turku, Finland; [3]InFLAMES Research Flagship Center, University of Turku; [4]Department of Clinical Chemistry, Fimlab Laboratories and Finnish Cardiovascular Research Center Tampere, Faculty of Medicine and Health Technology, Tampere University, 33100 Tampere, Finland; [5]Turku PET Centre, University of Turku, Kiinamyllynkatu 4-8, 20520 Turku, Finland; [6]Turku Center for Disease Modeling, University of Turku, 20520 Turku, Finland; [7]Turku PET Centre, Turku University Hospital, 20520 Turku, Finland
*Correspondence: tiit.ord@uef.fi (T.Ö.), minna.kaikkonen@uef.fi (M.U.K.)
https://doi.org/10.1016/j.ajhg.2023.03.013.
© 2023 The Author(s). This is an open access article under the CC BY license (http://creativecommons.org/licenses/by/4.0/).

To shed light into these questions, we performed scRNA-seq of thoracic aortas of healthy and atherosclerotic *Ldlr*$^{-/-}$/*Apob*$^{100/100}$ mice during disease progression. Our results identify 12 cell states that are increased in response to atherogenic changes. We validate our findings in bulk RNA-sequencing (RNA-seq) studies in a separate mouse experiment and in human samples from affected individuals. We identify common pathways, transcription factors, and ligands that define the cell state changes. Finally, we link our findings to GWASs to prioritize the relevance of atherosclerosis-associated cell states in the biology and heritability of CAD and apply this knowledge in polygenic risk prediction.

Material and methods

Mouse model

To model a disease stage course of atherosclerosis in mouse, we performed four combinations of genotype and dietary protocol. We fed the atherosclerosis-prone low-density lipoprotein receptor-deficient mice expressing only apolipoprotein B100 (*Ldlr*$^{-/-}$/*Apob*$^{100/100}$)[12,13] (The Jackson Laboratory strain #003000) a high-fat diet (HFD; Teklad TD.88137) for 0, 1, or 3 months to model prelesion, early, and late disease stages, respectively. We timed diet-starting age to equalize the age at sample collection between all groups (8 months old). Age-matched wild-type C57BL/6J mice fed chow diet were used as a healthy control. Male and female mice were used (mouse counts for each experiment are stated below). Throughout the study, mice were maintained on a 12-h light-dark cycle and had access to food and water *ad libitum*. All animal experiments were approved by the local ethics committee and carried out in compliance with European Union Directive 2010/63/EU.

Aorta single-cell RNA-seq library preparation

Mice were anesthetized with isoflurane and euthanized by cervical dislocation. The mice were perfused by cardiac puncture with 10 mL of ice-cold PBS supplemented with 20 U/mL heparin and placed on ice for dissection. The entire thoracic aorta (i.e., the ascending aorta, the aortic arch, and the descending aorta up to the diaphragm) was extracted and used in scRNA-seq sample preparation. The adventitia was mechanically removed under a dissection microscope and discarded. Each thoracic aorta minced with a scalpel and enzymatically dissociated into single-cell suspension in 0.8 mL of Multi Tissue Dissociation Kit 1 (Miltenyi Biotec #130-110-201) enzymatic mixture reconstituted in RPMI 1640 medium supplemented with 0.5% bovine serum albumin (BSA) and 20 mM HEPES buffer (pH = 7.2). The mixture was incubated at 37°C with end-over-end rotation. After 20, 40, and 60 min of incubation time, tissue pieces were left to settle for 10 s, and 0.8 mL of cell suspension supernatant was collected from the tube and placed on ice. To continue the digestion, we added 0.8 mL of fresh enzymatic digestion mixture. The collected cell suspension was filtered through a 30 μm cell strainer, centrifuged at 400 g for 8 min at 4°C, resuspended in PBS supplemented with 1% BSA, and placed on ice. The three cell suspension fractions for each aorta were subsequently pooled together and red blood cell lysis was carried out by adding 9 volumes of ice-cold 1X RBC Lysis Buffer, Multi-species (eBioscience #00-4300-54) and incubating on ice for 3 min. Subsequently, we added PBS to normalize the buffer and collected the cells by centrifugation (400 g for 8 min at 4°C). Magnetic removal of dead cells was carried out with the Dead Cell Removal Kit (Miltenyi Biotec #130-090-101) with Miltenyi MS magnetic columns following the manufacturer's instructions. Cells were resuspended in PBS containing 0.04% BSA and counted by hemocytometry with trypan blue staining. Cell viability was between 74% and 85%. In each experiment group, aortic cell isolation was carried out separately from three male mice, the cells were stained with TotalSeq cell hashing antibodies (BioLegend) according to the manufacturers' recommendations, and subsequently the cells from individual mice were pooled in equal proportions into one lane of the Chromium Controller microfluidics chip (10x Genomics). We only used male mice to minimize experimental variation and increase sensitivity of the experiment to detect disease-associated changes. This decision was justified on the basis of bulk RNA-seq results (below), which indicated that the effect of treatment on the major cell type proportions was stronger than the effect of individual factors such as sex (Figure S8C). We used the Chromium Single Cell 3′ Kit (v2 Chemistry; 10x Genomics) to prepare scRNA-seq libraries. Paired-end high-throughput sequencing was carried out on an Illumina NovaSeq instrument (read 1: 26 bp, read 2: 91 bp).

Aorta bulk RNA-seq library preparation

Mouse aortas were dissected as for scRNA-seq (described above), flash-frozen, and cryogenically pulverized with the Cellcrusher cryo-press cooled with liquid nitrogen. Total RNA was isolated and treated with DNaseI with the Absolutely RNA Nanoprep Kit (Agilent). RNA was quantified with the Bioanalyzer RNA 6000 pico assay (Agilent) and RNA-seq libraries were prepared with the SMARTer v2 Stranded Total RNA-Seq Pico Kit (Takara Bio). Sample size for bulk RNA-seq was six per group, consisting of four males and two females (except the 3-month HFD group, which had four males and one female). High-throughput Illumina sequencing (read length 75 bp, single-end) was carried out at EMBL GeneCore.

Single-cell RNA-seq data analysis

Sequencing reads were processed with the Cell Ranger pipeline (version 3.0.2; 10x Genomics) and the 10x Genomics mm10 reference transcriptome package (version 3.0.0). The Cell Ranger-filtered cell barcode count matrices were subsequently processed with Seurat (version 3.1.0)[14] running in R version 3.5.3. The standard (log normalization-based) workflow recommended by Seurat authors was used. For each library, to remove low-quality barcodes, we visually evaluated cell quality metrics (genes per barcode, UMIs per barcode, and mitochondrial read fraction) by using violin plots and selected cutoffs, which resulted in retaining 87%–93% of barcodes. In total, 36,157 cells were retained with median 1,629 (mean 2,043) UMI counts per cell, median 875 (mean 965.4) genes per cell, and median 5.8% (mean 5.8%) mitochondrial counts. Cells from all libraries were integrated with the canonical correlation analysis (CCA) method implemented in Seurat version 3[14] with default parameters. After CCA, the standard count processing was rerun on the integrated assay with default parameters, except for the number of principle components used (set to 35) and the clustering resolution (set to 1.1). The resulting 23 clusters were manually annotated to a general cell type level with the following marker genes: macrophage (*Csf1r*, *Cd68*, *Adgre1*), smooth muscle (*Myh11*, *Tagln*, *Cnn1*), endothelial (*Pecam1*, *Cdh5*, *Cldn5*), pericyte (*Rgs5*), dedifferentiated SMC (*Vcam1*,

Lgals3, *Dcn*), chondrocyte-like (*Comp*, *Fmod*), fibroblast (*Fbln1*, *Pdgfra*, *Serpinf1*), mesenchymal stromal (*Ly6a*, *Pi16*), NK/T (*Cd3d*, *Cd8b1*, *Nkg7*), and epithelial-like (*Upk3b*). Hashtag assignment was carried out with the HTODemux function of Seurat. As the Biolegend TotalSeq cell hashing staining reagent is a mixture of anti-CD45 and anti-MHC I antibodies, HTODemux was run separately for the immune and non-immune cells of each library. To remove ambient RNA contamination, we ran the DecontX function (celda R package version 1.1.6)[15] by using the automated clusters (resolution 1.1) as the cell population labels and we used the decontaminated counts for subsequent analysis. Cells with an estimated ambient RNA contamination fraction > 0.3 were excluded from downstream analyses as possible doublets or otherwise low-quality cells.

To identify disease-associated cell states, we divided the Uniform Manifold Approximation and Projection (UMAP) plot from the integration of all libraries into a grid of 200 by 200 (for visualization) or 50 by 50 (for cell selection) subdivisions and counted the number of cells from each library falling into each sector. The cell occupancy was normalized to the total number of cells in each library, scaled to a library size of 10,000 cells, and $\log_2$-transformed. Subsequently, for each sector, the fold change in cell abundance was calculated between the late disease sample and the healthy control sample. UMAP regions displaying a more than 2-fold (>1 $\log_2$ fold change) increase in cell occupancy in response to disease were selected for further investigation.

Marker genes of disease-increased UMAP regions (cell states) were defined with the Wilcoxon rank-sum test (implemented in Seurat) comparing the cells located within the area of increased UMAP occupancy to the other cells of the same general cell type (i.e., cells not changed in abundance upon disease). Genes expressed in at least 10% of cells (in at least one of the cell populations of the comparison) were considered and the minimum required log fold change was 0.25. p values were corrected for multiple testing with false discovery rate (FDR) and $p_{adj} < 0.05$ was considered significant. One highly reliable marker gene was selected to name the cell state whenever possible. The full marker lists are available in Table S2.

Gene Ontology enrichment analysis for cell state marker gene lists was performed with the g:Profiler web tool (access date 2020-03-26).[16] All cell state marker gene lists were tested for enrichment against all Gene Ontology Biological Process (GO:BP) categories. The cell state SMC-4 was not included in this analysis, as it had only three marker genes. To reduce redundant and overly general hits, we filtered the GO:BP enrichment results for each cell state marker gene list to remove categories with >1,000 genes in mouse and we further filtered for GO term semantic similarity by using the GOSemSim package (version 2.8.0)[17] by ranking the GO terms on the basis enrichment p value and, starting from the top, removing any less significant terms with Rel similarity measure > 0.9. After the GO term filtering, the top four most significantly enriched GO:BP terms were selected from each cell state (provided the terms satisfy $p_{adj} < 0.05$) for a comparative enrichment heatmap.

To plot gene set enrichment at the single-cell level, we used the VISION package (version 2.0.0)[18] with the GO:BP and MSigDB Hallmarks[19] gene sets.

To predict which extracellular ligands may serve as upstream inducers of a cell state, we used the NicheNet package (version 0.1.0).[20] For the query gene signature (i.e., the gene set to be explained by ligand-receptor interactions), an entire list of marker genes for a disease-associated cell state was used (described above). The cells of the disease-increased cell state were considered the "receiver" cell population (expressing receptor) and all other cell types in the dataset were considered potential "sender" cells (expressing ligands). A ligand or receptor was considered expressed in a cell population if detected in at least 10% of the cells. Predicted ligand activities were ranked with the Pearson correlation coefficient (default).

We used SCENIC (version 1.1.1)[21] to predict transcriptional regulators, motifs, and regulons that are preferentially active in disease-associated cell states. SCENIC single-cell regulatory network inference was run following the published vignette for one cell type at a time. Cells from the maximally different disease stages (control and late disease) were used. For SMC-related cells, cells were further randomly subsampled with the automated clusters (resolution 1.1; described above), keeping a maximum of 200 cells per cluster. The cisTarget database was mouse mm9 transcriptional start site (TSS)-centered ($\pm$10 kb), v9 motifs, seven species (download date 2020-01-24). The activity of each predicted gene network was scored in each cell and binarized, as described by the SCENIC authors. We used the Wilcoxon rank-sum test to find regulons that are differentially active in one cell state compared to the other cells of the same cell type. For differential activity, the regulon was required to be active in at least 20% of the cells of one population and satisfy log fold change > 0.15 and FDR < 0.05.

Decomposition of cell states in mouse bulk RNA-seq

We trimmed mouse aorta RNA-seq reads by using Trim Galore (version 0.4.4; GitHub: https://github.com/FelixKrueger/TrimGalore) to remove adapters and bases with quality score below 20. We aligned reads to the mm9 mouse genome with STAR (version 2.5.4)[22] and used HOMER (version 4.9)[23] to generate a gene count matrix from uniquely mapped reads.

To evaluate relative changes in cell state abundance between atherosclerosis disease stages based on mouse aorta bulk RNA-seq profiles, we applied the Cell Population Mapping method (scBio R package; version 0.1.5).[24] Aorta scRNA-seq cells and the integrated UMAP were used as the cell state space. The bulk RNA-seq count matrix was normalized for transcript length with reads per kilobase per million reads mapped (RPKM) and log-transformed. Cell Population Mapping was run in relative abundance mode (subtracting the mean of the reference group from all samples in the test group) with default parameters.

Cell state gene signature activity in human atherosclerosis study cohorts

Gene expression microarray data was obtained for human aortic plaque (n = 15; abdominal aorta), carotid plaque (n = 29), femoral plaque (n = 24), and non-atherosclerotic control artery (n = 24; left internal thoracic artery) from the Tampere Vascular Study[25] and from human carotid artery segments classified as either advanced (n = 16) or early (n = 13) atherosclerotic plaque (GEO: GSE28829).[26] Gene expression levels were quantile-normalized with the preprocessCore R package (version 1.52.1; GitHub: https://github.com/bmbolstad/preprocessCore). To calculate gene signature activity scores in these bulk RNA profiles, we used the approach of Tirosh et al.[27] (implemented as the Seurat function AddModuleScore), wherein genes are binned on the basis of average log expression level across samples, and in each sample, a bin background level (calculated from random control genes in the same bin) is subtracted from the levels of the test genes. The

full lists of marker genes of atherosclerosis-increased cell states were used as the tested gene sets (gene programs).

Prioritized genes at human CAD GWAS loci

To associate human CAD GWAS loci to their potential causal genes, we collected published gene prioritization results across multiple different prioritization approaches and GWASs.

We used the OpenTargets Genetics Portal (data release: version 5–21.06)[28] to obtain data for the following published GWASs of CAD, myocardial infarction, and stroke[29–35] and also for the biobank-based GWAS results[36,37] for FinnGen coronary atherosclerosis and UK Biobank ischemic heart disease, myocardial infarction, and coronary atherosclerosis (OpenTargets study accessions FINNGEN_R5_I9_CORATHER, SAIGE_411, SAIGE_411_2, and SAIGE_411_4). Across the studies, the OpenTargets Locus2Gene algorithm, a machine learning method trained on a gold-standard curated result set, prioritized 238 unique genes, OpenTargets eQTL colocalization 164 genes, and OpenTargets "nearest gene" 387 genes. The recent very large CAD GWAS by Aragam et al.[9] was additionally included. From this study, the per-association overall top-prioritized genes contributed 186 unique genes, polygenic priority score (PoPS) 386 genes, and "nearest gene" 216 genes, and for the GWAS 1% FDR threshold loci, "nearest gene" contributed 716 genes. Additionally, the transcriptome-wide association study (TWAS) of CAD by Li et al.[38] prioritized 114 unique genes and the CAD GWAS review by Erdmann et al.[39] listed 373 genes at CAD loci.

Partitioned heritability with linkage disequilibrium score regression (LDSC)

LDSC (version 1.0.1)[40] and the van der Harst et al. CAD GWAS[29] full summary statistics (downloaded from Mendeley Data: https://doi.org/10.17632/gbbsrpx6bs.1) were used for partitioned heritability analysis of CAD following the recommendations published by LDSC authors. The marker genes of atherosclerosis-associated cell states (described above) were used as the gene sets. The gene-transcribed regions in the hg19 human genome were padded with 100 kb upstream and downstream. The LD score window was 1 cM (default), the provided 1000 Genomes (1000G) Phase 3 EUR data files were used, and the 1000G Phase 3 EUR baseline models were included in the calculation.

Accessible chromatin by cell type in human atherosclerotic plaques

We used human endarterectomy single-cell assay for transposase-accessible chromatin with sequencing (scATAC-seq)[11] transposase cut site coordinates and cell annotations (available at FigShare: https://doi.org/10.6084/m9.figshare.14501985.v2) to aggregate cut sites to pseudobulk at the level of cell lineages (SMC, EC, macrophage, T/NK, and B/plasma) and we called peaks in each cell lineage separately by using MACS2[41] with fragment extension 200 bp and shift −100 bp. Where equal-width peaks are stated, we resized peaks centering on the MACS2-called peak summit coordinate (cell type specific).

Gene set-based PRSs

We used the PRSice-2 (version 2.3.3)[42] feature PRSet,[43] which performs region-aware (coordinate set-based) SNP clumping, to generate clumping and thresholding PRSs based on sets of cell state marker genes, scATAC-seq peaks, or genes of a biological pathway. In PRSet, SNPs falling within regions of interest are preferentially retained for each LD clump of SNPs. The clumping distance was 500 kb to either side of the index SNP and the LD r^2 threshold was 0.2.

The base data for all PRSs were the additive model summary statistics of the CARDIoGRAMplusC4D 1000 Genomes-based CAD GWAS,[44] a study that does not have significant sample overlap with the UK Biobank.[29] We filtered base data genetic variants to keep variants with INFO score $\geq$ 0.8 and to exclude variants with strand-ambiguous alleles.

The UK Biobank[45] was used as the target cohort for all PRS scoring. We filtered the imputed genotype data to keep autosomal variants with MAF $\geq$ 0.01 and INFO score $\geq$ 0.8 and exclude variants with Hardy-Weinberg equilibrium test $p < 1e{-}25$ or genotype missingness rate > 0.1 by using PLINK (version 2.00).[46] The final variant set across base and target data consisted of 4,958,173 variants. We included participants of self-reported White ancestry in the precomputed UK Biobank PCA calculation (unrelated samples) and selected available imputed genotype data and filtered to exclude participants with sex chromosome aneuploidy, heterozygosity or genotype missingness outliers, participants with excess relatives, kinship inference analysis exclusions, those more than seven standard deviations away from mean in the first six principal components (PCs) (GitHub: https://github.com/Nealelab/UK_Biobank_GWAS), and participants who had withdrawn consent. CAD phenotype status in the UK Biobank was defined on the basis of the example of Choi et al.[43] (specifically, GitLab: https://gitlab.com/choishingwan/prset_analyses/-/blob/master/script/sql/generate_cad.sql), which defined CAD cases by using primary or secondary ICD10 or ICD9 codes for hospital inpatient records or cause of death and additionally OPSC4 operation codes and self-reporting by the participant. In total, there were 21,600 cases and 359,254 controls.

For gene set-based PRSs generated from gene coordinates alone (i.e., not using scATAC-seq regulatory region information), we extended the transcript regions to include 35 kb upstream and 10 kb downstream. To utilize scATAC-seq peaks to inform cell state marker gene PRSs, we only considered the scATAC-seq peaks called in the corresponding cell type and peaks within ±500 kb of the gene TSS were retained for PRS generation.

PRS performance was evaluated as set out in the PRSet method.[43] We evaluated variance explained by the PRS ($PRS.R^2$) by subtracting the pseudo R^2 of the full model ($CAD \sim PRS +$ covariates) from the pseudo R^2 of the null model ($CAD \sim$ covariates). The covariates used in PRS modeling were sex, age (earliest age of CAD for cases, oldest age at attending UK Biobank center for controls), UK Biobank assessment center, genotype batch, and the first ten genetic PCs. We used the PRSet competitive p value calculation,[43] based on permutation testing (10,000 permutations), to test for signal enrichment compared to identically clumped SNPs in regions of the genome considered background (defined as either all genes or all scATAC peaks).

Results

Identification of atherosclerosis-associated cell states

To provide an unbiased, enrichment free, analysis of all the cell types of the healthy and atherosclerotic vascular wall, we performed scRNA-seq from single cell suspensions extracted from thoracic aorta. Cells from three mice were labeled with hashtag antibodies and pooled from each of four conditions, including healthy control

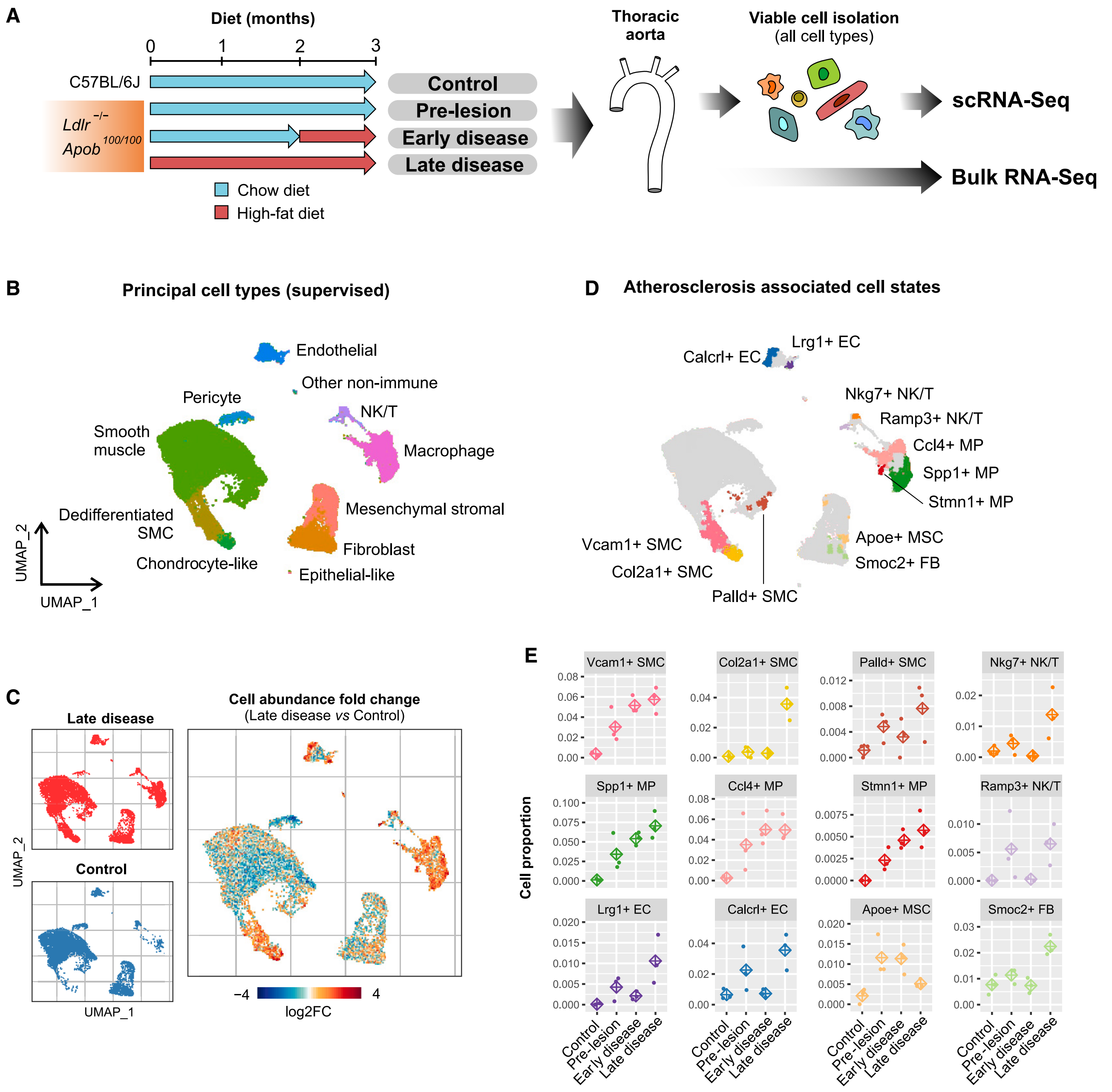

Figure 1. Clustering and identification of atherosclerosis-associated cell states
(A) Schematic overview of the experimental setup.
(B and C) (B) UMAP projection of the scRNA-seq profiles represented as eleven manually annotated clusters. (C) UMAP regional occupancy analysis demonstrating relative changes in cell density comparing atherosclerotic vascular wall to healthy controls. Atherosclerosis-associated cell states are revealed by increased local abundance of cells in regions of the UMAP plot (log2FC, log2 fold change).
(D) UMAP plot depicting the 12 disease-associated cell states and the selected top marker genes.
(E) Relative changes in the cell state proportions during different stages of atherosclerosis shown for each of the three biological replicates. Diamond represents the average of the three replicates.

mice and *Ldlr*$^{-/-}$/*Apob*$^{100/100}$ mice with pre-lesioned, early, and late atherosclerotic lesions, and prepared into libraries with the 10× Chromium system (Figure 1A). The scRNA-seq profiles of 36,157 cells passed quality control and were selected for downstream analysis (Figure S1). Joint analysis of atherosclerotic and control samples with automated clustering identified 23 cell clusters that corresponded to nine major cell types, including endothelial cells (ECs), NK/T cells, monocyte/macrophages (MPs), smooth muscle cells (SMCs), pericytes, dedifferentiated SMCs, chondrocyte-like cells, mesenchymal stromal cells (MSCs), and fibroblasts (FBs) according to marker gene-based curation (Figures 1B and S2 and Table S1).

Extensive variability in the regional occupancy of the Uniform Manifold Approximation and Projection

(UMAP) was observed across different disease stages after data integration, indicating a spectrum of cellular states associated with atherosclerosis. To define discrete cell states (within broader cell types) that are atherosclerosis associated, we selected regions of the UMAP plot where the local cell density was increased >2-fold in late disease compared to control. (Figures 1C and S3). Differential neighborhood abundance analysis based on the *k*-nearest neighbor graph (Milo method[47]) largely confirmed the regions of altered abundance (Figure S4). The marker genes of disease-increased cell states were defined by comparing the cells located within the area of increased UMAP occupancy to the other cells of the same cell type that did not change in abundance (full marker lists in Table S2). Therefore, it should be noted that the cell state markers represent a distinct signature from the general cell type markers that are obtained by comparison between cell types (Tables S1 versus S2). Using the marker gene sets to score gene program activity revealed that the cell state signatures tend to be upregulated gradually, and the cells within the defined cell state showed >1 SD unit of elevated expression relative to the expression variation in the cell type as a whole (Figure S5).

Our analysis identified 12 cell states that were increased in at least one of the diseased conditions (Figure 1D), which were named on the basis of one of the top marker genes. These included three SMC cell states, including Vcam1+ SMC, Col2a1+ SMCs, and Palld+ SMCs, three macrophage-derived cell states called Spp1+ MPs, Ccl4+ MPs, and Stmn1+ MPs, two EC cell states corresponding to Lrg1+ ECs and Calcrl+ ECs, and two NK/T cell states, Nkg7+ NK/Ts and Ramp3+ NK/Ts, as well as Smoc2+ FB state and Apoe+ MSC state. We noted a further candidate population of disease-increased cells among the contractile (Myh11+) SMCs (Figures S2B, S4, and 1D). However, it appeared to be a very mild perturbation, showing only three marker genes (*Slc22a1*, *Rbp4*, *Ndufa3*), and therefore was not included in subsequent analyses. Importantly, the cell states demonstrated temporal patterns corresponding to the progression of the disease. For example, the proportion of cells associated with EC and MP states as well as Vcam1+ SMC and Palld+ SMC state cells exhibited a gradual increase toward late disease stage, whereas NK/T states, Col2a1+ SMCs, and Smoc2+ FBs were mostly detected at the late disease stage (Figure 1E). This highlights the importance of temporal resolution in scRNA-seq-based analysis of disease progression. Among the cell state markers, we selected LRG1, VCAM1, and PALLD for validation with immunohistochemistry (Figure S6) and *Lrg1* and *Palld* for confirmation by using spatial transcriptomics with the Molecular Cartography platform by Resolve Biosciences (Figure S7). In addition, we used alternative marker genes to visualize the Vcam1+ SMCs (*Col6a3*), Col2a1+ SMCs (*Col6a3* and *Sox9*), Ccl4+ MPs (*Il1b*), Spp1+ MPs (*Abca1*), and Stmn1+ MPs (*Top2a*) because the primary marker genes of these states exceeded the maximum expression level limit and had to be excluded at the panel design stage (Figure S7). Altogether, this analysis confirmed the distinct location and identities of several of the cell states.

Validation of the atherosclerosis-associated cell states in mouse and human lesions

To confirm that the identification of atherosclerosis-associated cell states is not confounded by technical issues related to scRNA-seq, we further sough to analyze the presence of the gene signatures in bulk RNA-seq. In particular, cell dissociation could alter the relative proportions of cell populations in scRNA-seq experiments, an effect that would not occur with bulk RNA-seq.[48] We therefore performed a parallel bulk RNA-seq experiment with 5–6 replicates and estimated the performance of these two methods in capturing cell type proportions during atherosclerosis progression (Figure 1A). Altogether 1,049 unique genes were upregulated and 851 downregulated during the course of disease development (Table S3). Notably, a large majority of the bulk RNA disease-induced genes were expressed in immune cells, in line with expected infiltration of these cells to the vascular wall during disease progression and indicating that changes in cell type proportion are a major driver of bulk RNA-seq differential expression (Figures S8A and S8B). Deconvolution of bulk RNA-seq data with the scRNA-seq data as in Newman et al.[49] confirmed the gradual increase of macrophage and NK/T cells relative to other cell types (Figure S8C). This was also evident from the scRNA-seq data itself, although the relative proportion of leukocytes was smaller compared to bulk RNA deconvolution results, possibly reflecting selective cell loss due to tissue dissociation (Figure S8D). To further leverage the bulk RNA-seq data to validate the atherosclerosis-associated cell states, we estimated the relative cell state abundance changes by using cell population mapping,[24] where scRNA-seq profiles are used to infer the composition of cell states from bulk transcriptome data. This analysis supported the increase in cell state abundance for majority (9/12) of the cell states but failed to recapitulate Palld+ SMCs, Smoc2+ FBs, and Apoe+ MSC state changes, possibly because of the low amount of cells in these populations (Figure 2A). Altogether, this analysis supports the reliability of our workflow for cell state identification from single cell expression data.

We next sought to investigate the reproducibility of the atherosclerosis-associated cell states in public datasets of mouse and human scRNA-seq. To achieve this, we compared the enrichment of gene set modules in the cell type of interest in our data, in different mouse models of atherosclerosis[4] and in human coronary lesions[3] (Figures S9–S14). In the Pan et al.[4] dataset including *Ldlr*$^{-/-}$ (0, 8, 16, and 26 weeks of HFD) and *Apoe*$^{-/-}$ (8, 16, and 22 weeks of HFD) mouse models, the cell state markers were enriched in a specific subpopulation of cells, suggesting that they represent distinct phenotypes (Figure S11). The fraction of gene set positive cells were increased upon disease progression for all other cell types

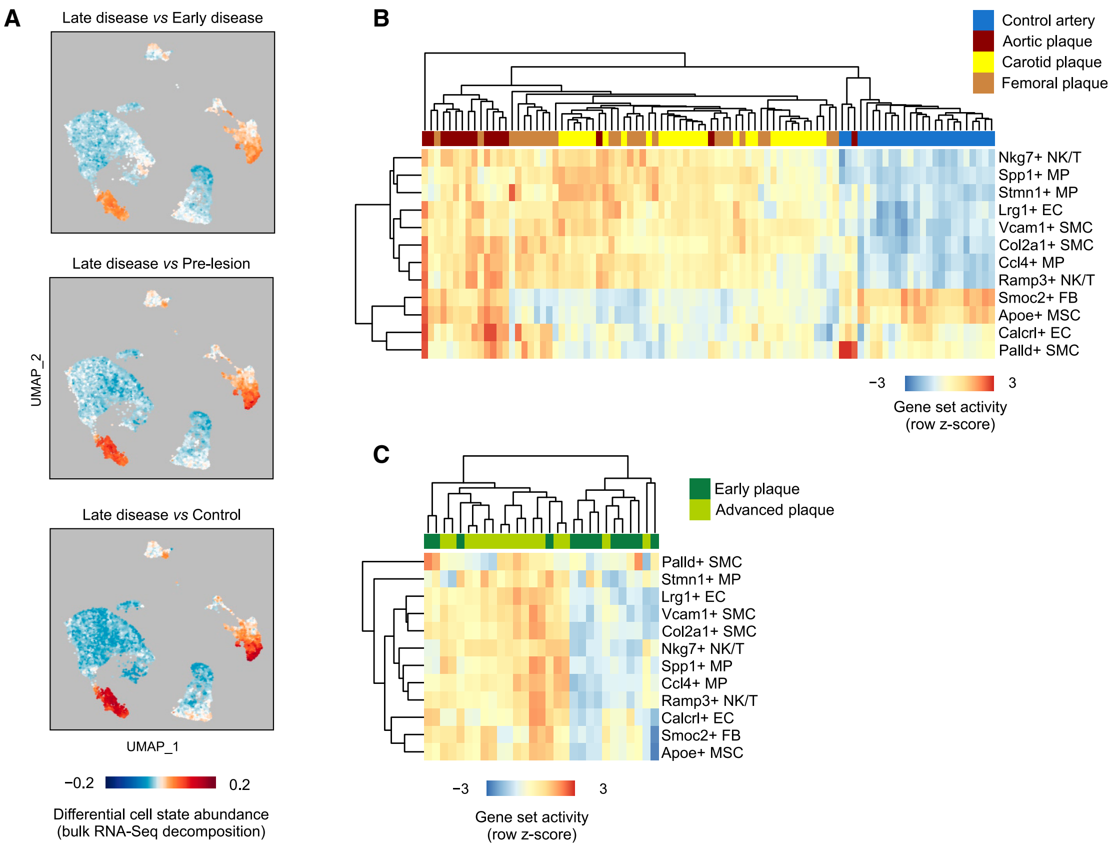

Figure 2. Atherosclerosis-associated cell state signatures are activated in mouse and human lesions based on bulk RNA-seq
(A–C) (A) Cell population mapping using the scRNA-seq cell state space to plot the differential cell abundance estimated from the mouse bulk RNA-seq data. The gene set activity scores for each cell state were further investigated in the (B) Tampere Vascular Study[15] representing 68 advanced atherosclerotic plaques (15 aortic, 29 carotid, and 24 femoral plaques) and 28 controls (left internal thoracic artery) and (C) the Maastricht Pathology Tissue Collection[16] representing atherosclerotic carotid artery segments from 13 early intimal thickening/xanthoma lesions and from 16 advanced fibrous cap atheroma lesions.

except the Palld+ SMC and Calcrl+ EC states, which demonstrated a relative decrease from the earliest time-points in both mouse models (Figures S12 and S13). In addition, FB and MSC states were only increased between 8 and 16 weeks of HFD in the *Ldlr*$^{-/-}$ mouse model, whereas NK/T states exhibited a decrease in cell fraction between 16 and 26 weeks of HFD, suggesting mouse-model- and time-point-specific differences.

In human lesions,[3] all the cell state markers with the exception of Calcrl+ ECs had a distinct localization within the cell type cluster (Figure S14), supporting that the same cell states are present. However, the available human scRNA-seq datasets do not allow comparison between healthy and diseased vasculature, which is why we further extended our analysis to bulk RNA-seq datasets to investigate whether the cell state signatures were associated with disease progression. To achieve this, we studied the expression of the cell state markers in two human clinical cohorts, the Tampere Vascular Study,[25] representing 68 advanced atherosclerotic plaques (15 aortic, 29 carotid, and 24 femoral plaques) and 28 controls (left internal thoracic artery), and the Maastricht Pathology Tissue Collection,[26] representing atherosclerotic carotid artery segments from 13 lesions at the early intimal thickening/xanthoma stage and from 16 advanced fibrous cap atheroma lesions. Altogether, 10 out of the 13 disease state gene signatures separated the diseased samples from the controls (Figures 2B and 2C). The Palld+ SMC and Calcrl+ EC markers were only induced in the aortic plaques, compared to femoral or carotid plaques or control arteries, suggesting vascular-bed-specific differences. These results demonstrate similarities between the atherosclerotic cell states in mouse and human and support a potential role for these genes in disease etiology.

Identification of cell-state-specific and shared pathways among gene signatures

The maintenance and transition of cellular states are controlled by environmental signals that translate into gene regulatory mechanisms and biological processes. To investigate the similarity of the biological process activities of the atherosclerosis-associated cell states, we next evaluated the uniqueness of the cell state markers and their associated gene ontologies. Comparison of the marker gene sets of each disease-associated cell state demonstrated that the large majority (71%; 1,516/2,122) of the signature genes were

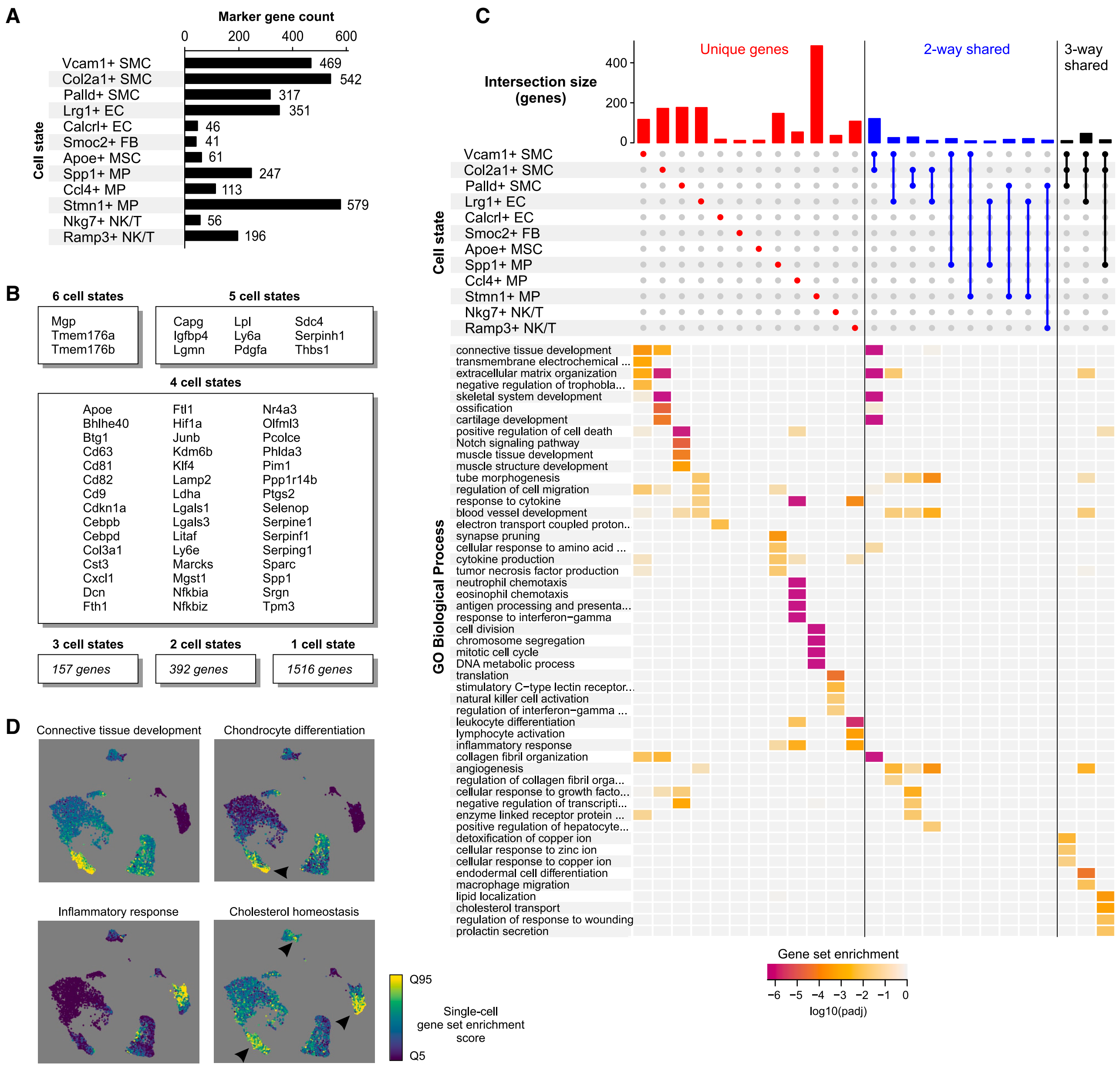

Figure 3. Characterization of atherosclerosis-associated cell states and key biological pathways
(A) Marker gene counts for the 12 most abundant disease-associated cell states.
(B) Common markers between disease-increased cell states.
(C) UpSet plot showing the gene overlaps between cell state signatures and the gene ontologies enriched (log10 of adjusted p value) in the intersections.
(D) Single-cell gene set enrichment scoring for selected biological processes. Enrichment score is shown from the lowest 5% (Q5) to highest 95% (Q95).

unique to one cell state (Figures 3A and 3B). Still, 12 genes were common to five or more cell states, as exemplified by matrix Gla protein (*Mgp*), stem cell antigen-1 (*Sca1*; also called *Ly6A*) and legumain (*Lgmn*), syndecan 4 (*Sdc4*), and insulin-like growth factor binding protein 4 (*Igfbp4*), and could thus represent global markers of atherosclerosis.

Comparison of marker gene lists at the level of gene ontology allowed to further shed light into the phenotypes of these cell states (Figures 3C and 3D; Table S4). For example, *Vcam1* itself and the other associated cell state markers participating in ECM remodeling have been previously identified as hallmarks of SMC-derived intermediate cells,[4] also called fibromyocytes.[3] These cells have been suggested to differentiate into chondrocyte-like cells,[2,5] i.e., fibrochondrocytes,[4] which correspond to the Col2a1+ SMC state. This gave us a unique opportunity to model a three-state continuum for the SMCs (Myh11+ - Vcam1+ - Col2a1+) not evident for the other cell types. The hypothesis proposing that contractile SMCs can undergo transdifferentiation into Vcam1+ SMCs and Col2a1+ SMCs was further corroborated by the results of pseudotime trajectory analysis (Figure S15).

Among ECs, the Lrg1+ ECs expressed markers of endothelial-to-mesenchymal transition, such as *Sox4*,[50] *Tubb3*,[51] and *Fbln5*,[52] in line with a recent report.[8] On the other hand, Calcrl+ EC state genes were indicative of regulation of endothelial function by mitochondrial reactive oxygen species (electron-transport chain) and shear stress (e.g., *Calcrl*, *Klf4*, *Pecam1*, *Tek* [*Tie2*], *Jun*, and *Fos*).[7] Our analysis also uncovered the phenotypes of the five atherosclerosis-associated immune cell states as similar to lipid-associated Trem2+ MPs (Spp1+ MP1), proinflammatory MPs (Ccl4+ MPs), proliferating MPs (Stmn1+ MPs),[1] Nkg7+ Cytotoxic CD8 T cells, and Ramp3+ antigen-specific CD4 T cells.[53,54]

Despite extensive cell state specificity of the marker gene sets, we also identified shared biological processes between some cell states. Gene ontologies related to ECM organization, blood vessel development, endodermal cell differentiation, and angiogenesis were enriched among the shared genes of Vcam1+ SMC, Col2a1+ SMC, and Lrg1+ EC states, whereas enrichment for cholesterol transport and homeostasis genes was shared between Vcam1+ SMC, Col2a1+ SMC, and Spp1+ MP states (Figures 3C and 3D). Taken together, these results provide evidence that the disease-associated cell states largely contribute to specific cellular functions in the atherosclerotic niche with, however, important overlapping activities related to vascular development, angiogenesis, and lipid metabolism.

Disease states share similar upstream regulators but differ in predicted TF activities

We next sought to investigate how the gene signatures of disease-associated cell states could be defined by the cellular microenvironment by inferring intercellular communications and intracellular signaling networks. We first looked for expressed ligands and receptors and modeled their gene regulatory effects in putative ligand-receiving cells for the five most abundant disease states by using the NicheNet tool[20] (Figure 4A). Ligands belonging to the transforming growth factor β (TGFβ), inflammation (TNF, IL-1α/β), angiogenesis (VEGFA, FGF1/2), and cholesterol pathways (ApoE) were identified as the most probable ligands to give rise to the gene sets, suggesting extensive similarities in the microenvironmental signals underlying cell-state-specific gene expression (Figures 4A and 4B). Among them, *Tnf* and *Il1a/b* were mostly expressed by the lipid-associated Spp1+ and proinflammatory Ccl4+ MPs, suggesting extensive auto- and paracrine proinflammatory signals originating from myeloid cells (Figure 4C). Importantly, these proinflammatory ligands were predicted to regulate several cell state genes specific for Vcam1+ and Col2a1+ SMCs (Figure 4B). To test the validity of these computational predictions, we stimulated mouse SMCs *in vitro* by using the top-ranked ligand IL-1β for 24 and 48 h and modeled the pseudotemporal trajectory of the gene expression response on the basis of scRNA-seq. Our results demonstrated that the genes upregulated along the *in vitro* SMC IL-1β response trajectory were induced along trajectory of *in vivo* SMC disease-associated transition and vice versa, supporting the predictions (Figure S16). Finally, Col2a1+ chondrocyte-like cells were predicted to be the main producers of TGFβ1, VEGFA, and BMP2, highlighting their potential role in a cell-cell signaling network of autocrine osteochondrogenic signaling and paracrine signaling driving the fibrogenic and angiogenic gene expression programs in Vcam1+ SMCs and Lrg1+ ECs (Figures 4D and S17).

The predicted similarities in the microenvironmental signals suggest that the differences in disease-associated gene signatures could arise as a result of differential response of the cell types to the same stimulus and thus cell-state-specific transcription factor (TF) activity. To investigate this, we inferred TF regulatory networks by single-cell regulatory network inference and clustering (SCENIC) analysis for the three major cell lineages (SMCs, ECs, and MPs; Figure 5A).[21] First, we predicted the transcription factors for the Palld+, Vcam1+, and Col2a1+ SMC-derived cell states (Figure 5B). The Palld+ cell state gene expression profile was predicted to be driven by proinflammatory TFs such as the NF-κB, AP-1, and Maf family members. In line with the predicted differentiation trajectory of the Vcam1+ and Col2a1+ cell states from contractile SMCs, the SCENIC predictions supported a gradient of TF activity (Figures 5E and S15C) and evolution of gene networks. CEBPD and RUNX1 were identified as the key regulators for both atherosclerosis-associated cell states despite the higher expression of *Cebpd* in Vcam1+ SMCs and *Runx1* in Col2a1+ SMCs. In addition, the transition from Vcam1+ cell state to Col2a1+ state was predicted to be driven by the ER-Golgi stress transducer CREB3 family members[55] as well as EndMT associated HAND2 and SNAI1 TFs.[56] TFs induced by inflammation and metabolic stress, including XBP1, ATF4, RARG, and NFATC1/2, were identified as key drivers of the Col2a1+ state.

In line with marker gene expression, SOX4 was predicted as the main driver of the Lrg1+ EC state, followed by XBP1, TCF4, CEBPB, IRF3, CREB3L2, and ELK3 (Figures 5C and 5E). The analysis was unable to identify candidate regulators for Calcrl+ EC state. Interestingly, XBP1, TCF4, CEBPB, CREB3L2, and ELK3 were also identified as key regulators of the Vcam1+ and Col2a1+ SMC states, suggesting a key role of these TFs across cell types. As expected, the inflammatory Ccl4+ MPs showed enrichment of proinflammatory TF motifs, such as STAT, IRF, and AP-1 factors, whereas the Spp1+ lipid-associated MPs were enriched for SREBF1, USF1, NR1H3, and the ER stress response TFs DDIT3 and XBP1, which all have been implicated in lipid homeostasis[57] (Figures 5D and 5E). The proliferating Stmn1+ MPs were enriched for TF motifs implicated in cell cycle regulation, as exemplified by the members of the E2F TFs and EZH2. Among the TFs identified, XBP1, MAFB, MLX, and NFE2L2 (also called NRF2) were also identified as drivers of the Vcam1+ and

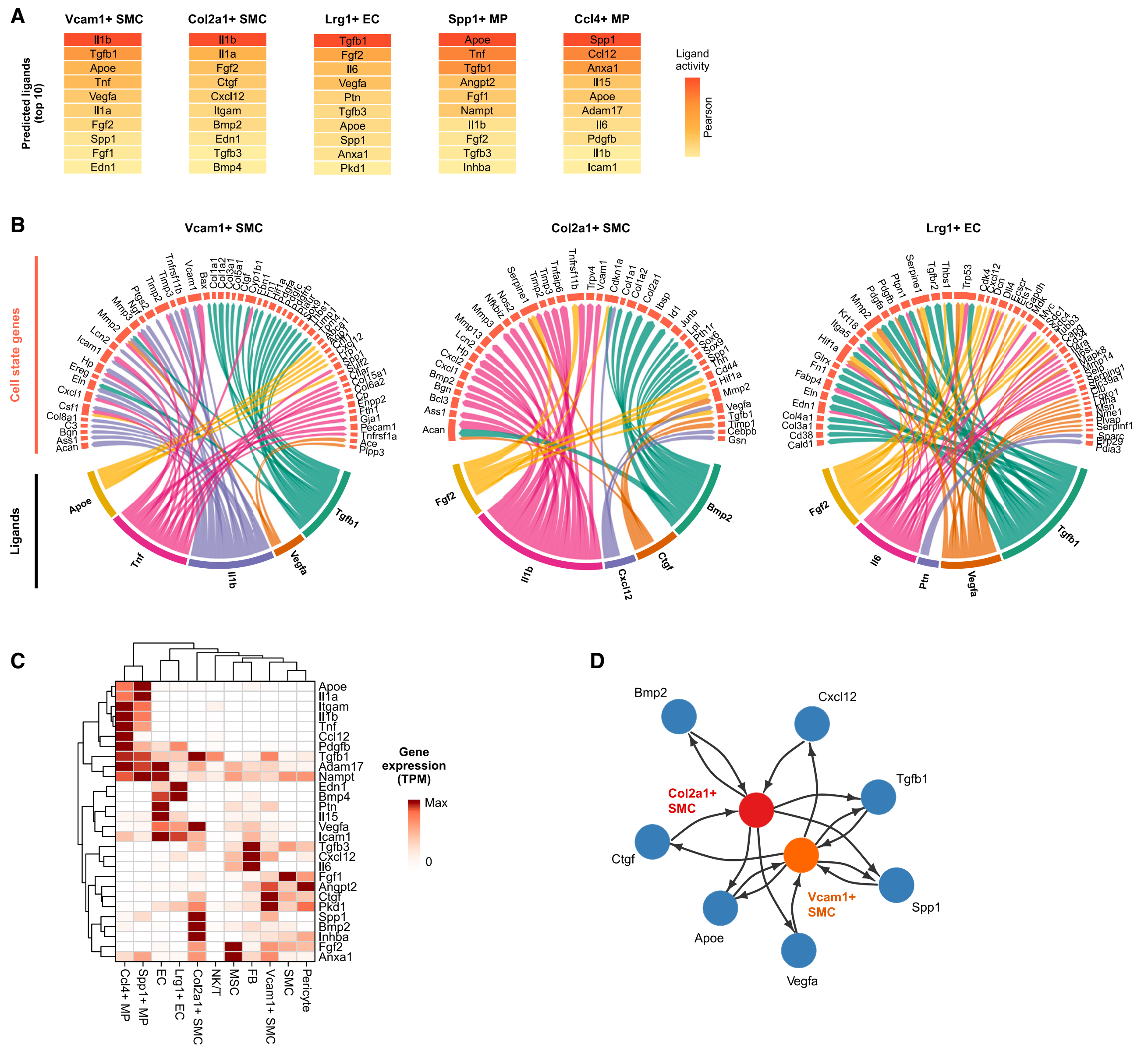

Figure 4. Modeling intercellular communication between cell states
Ligand–receptor–target gene analysis was carried out with NicheNet.
(A) Top 10 prioritized upstream ligands for cell state signature gene sets.
(B) Ligand target gene networks presented for Vcam1+ SMCs, Col2a1+ SMCs, and Lrg1+ ECs.
(C) Expression of the prioritized ligands by the different cell states and types. Row normalized gene expression (TPM = transcripts per million) is shown.
(D) Schematic of predicted ligand-mediated signaling between Vcam1+ and Col2a1+ SMC states involving autocrine and paracrine signaling.

Col2a1+ SMC states. Overall, we conclude that the majority of the single-cell gene regulatory networks are cell state specific and could provide critical insights into essential factors driving the progression of atherosclerosis.

Interrogation of GWAS loci and partitioning the heritability of CAD highlights the importance of smooth muscle cell states

GWASs have identified over 300 risk loci for CAD.[9,10,29] Nonetheless, for a vast majority of the loci, the causal gene(s) underlying the association are not known with certainty. In the last few years, however, several new methods have emerged that, on the basis of genomic proximity, protein-coding variants, variant association to gene expression (QTL, TWAS), enhancer-gene maps, or similarity in gene functions or pathways, establish links between risk loci and genes,[9,28,38,39] providing hundreds of candidate causal genes. We made use of these candidate causal gene lists to compute their enrichment among the cell state gene sets by using the hypergeometric test with expressed genes (>1 TPM in any aortic cell type or state; 14,902 genes) as the background. Our results demonstrate that

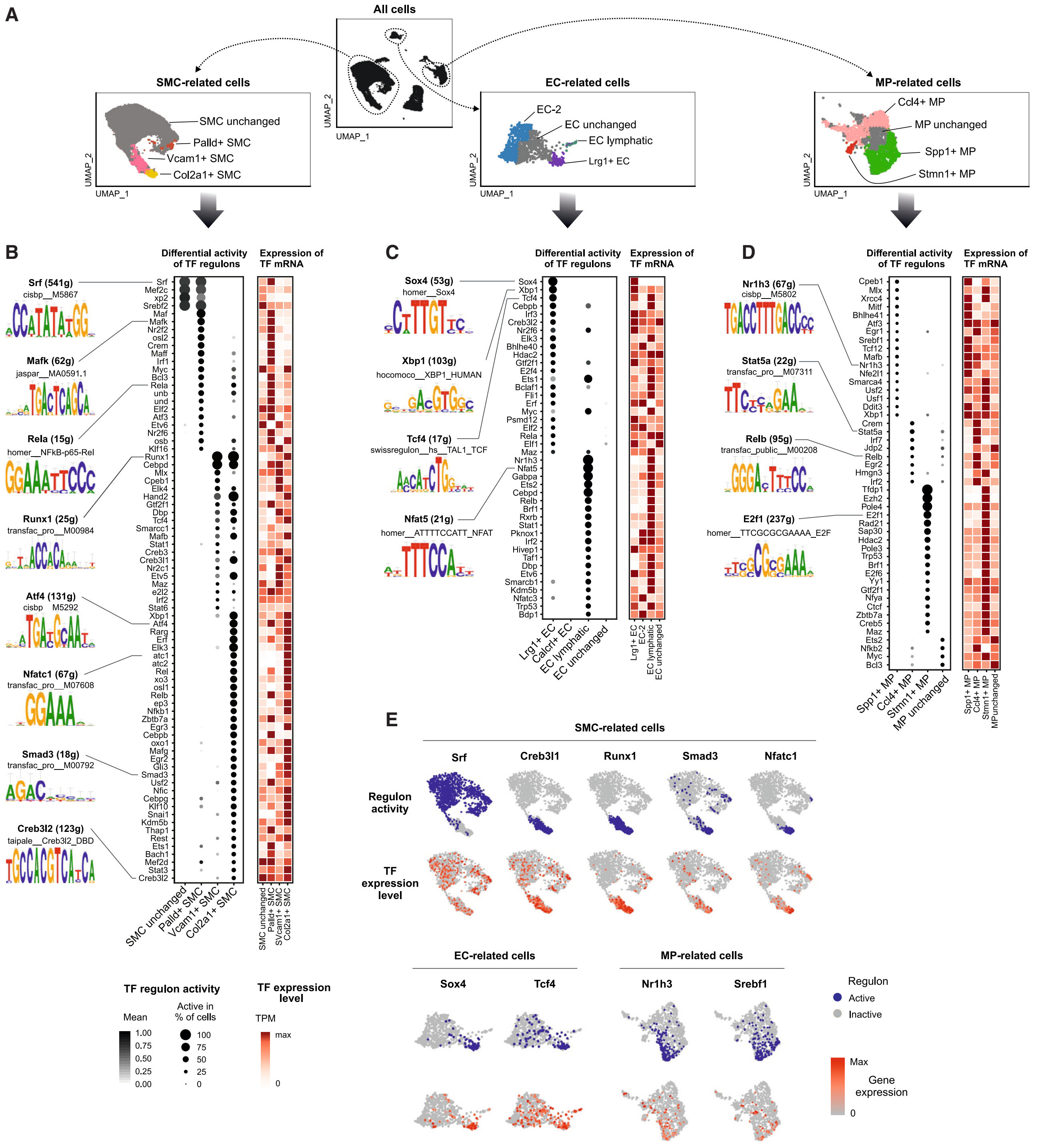

Figure 5. Prediction of cell-state-specific transcription factor activities

(A–D) (A) The most abundant atherosclerosis-associated cell states were selected for SCENIC analysis[21] along with disease-unperturbed cells of the same cell type. Differentially active gene regulatory networks identified for (B) smooth muscle cell (SMC), (C) endothelial cell (EC), and (D) macrophage (MP) cell states. The predicted regulon activity and transcription factor gene expression (row normalized TPM) are shown.

(E) Selected examples of regulon activities and transcription factor gene expression plotted on UMAP.

11 of the 12 cell states included genes prioritized as candidate causal genes, with the highest enrichment detected for the three SMC state (Vcam1+, Col2a1+ and Palld+) and Lrg1+ EC state genes (Figures 6A and S18). This enrichment trend was also evident when the marker gene lists were truncated to a specific number of top genes (Figure S19). In a further analysis, we equalized the number of marker genes for each cell state across a larger range (top 25 to 500 genes) by also including sub-threshold marker genes where needed. These results (Figure S20) also

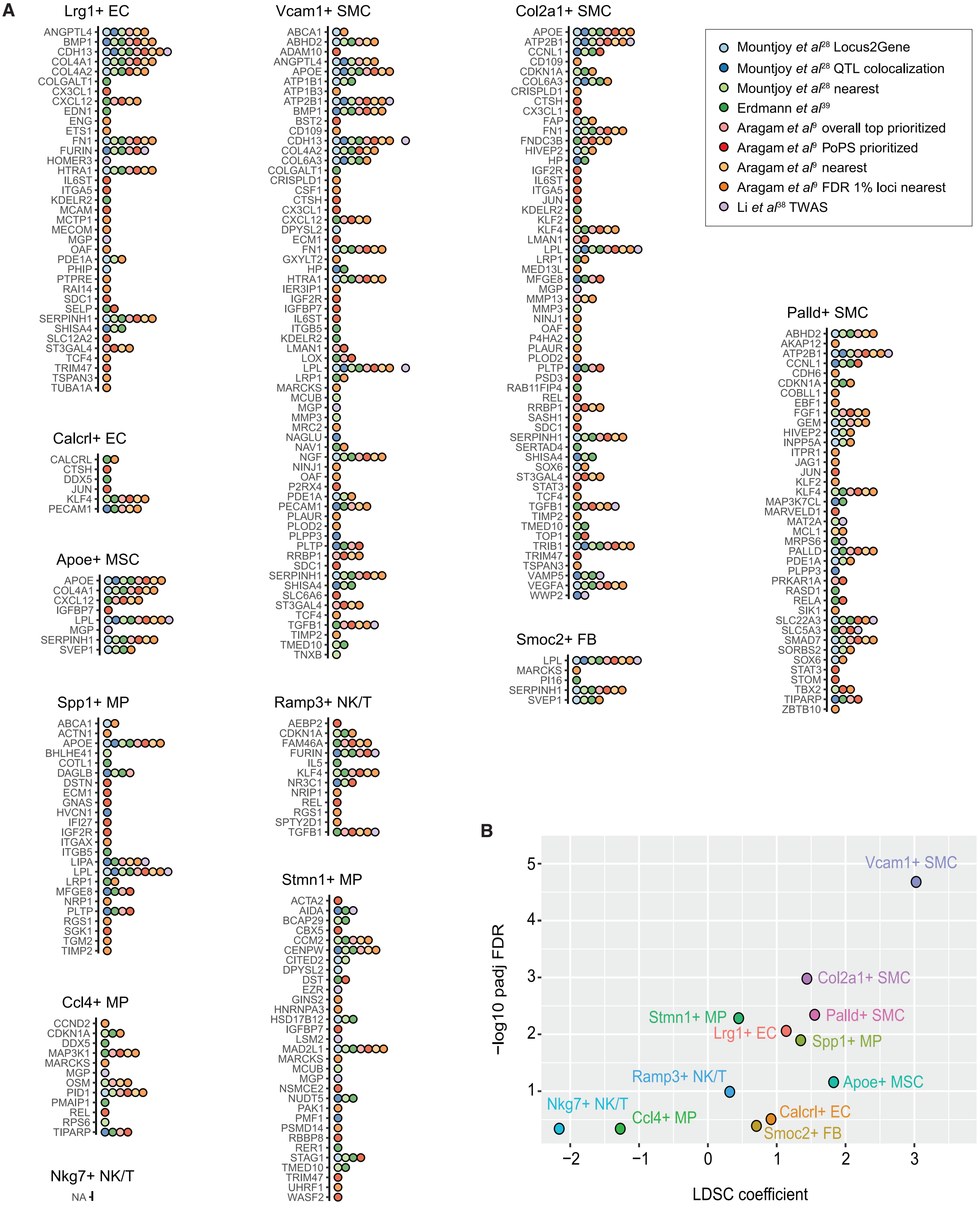

Figure 6. The contribution of cell states to CAD heritability

(A) Overlap of the CAD GWAS candidate causal gene lists from nine different sources (different colors) with the cell state markers. NA indicates no overlapping genes.

(B) Results from LD score regression (LDSC)[40] applied to cell state marker genes (using 100 kb flanking regions) to partition CAD heritability within the genome.

confirmed SMC (Vcam1+, Col2a1+, and Palld+) and EC (Lrg1+) states as the most enriched for CAD-GWAS-prioritized genes, including when measured by overlap ratio.

To further investigate the contribution of atherosclerosis-associated cell state signatures to the SNP-based heritability of CAD, we applied the LDSC tool[40] to partition heritability to gene sets by using CAD GWAS summary statistics. In our analysis, CAD heritability was most significantly enriched in regions surrounding Vcam1+ SMC state genes, followed by Col2a1+ and Palld+ SMC states, Spp1+ and Stmn1+ MPs, and Lrg1+ ECs (Figure 6B). Our results demonstrate significant enrichment of SMC cell state genes over all other cell types of the lesions, which could help in functionally interpreting the GWAS signal.

Cell-state- and pathway-specific PRSs provide insight into the biological mechanisms of CAD risk

An emerging body of evidence has shown that aggregation and weighing of CAD-associated variants into PRSs can improve an individual's risk prediction beyond traditional risk factors and provide an opportunity to identify novel mechanisms influencing CAD risk.[58] GWAS results could be considered a composite of signals corresponding to CAD-relevant processes encoded by different genomic regions and biological pathways. We therefore sought to test how much polygenic risk for CAD is influenced by the cell state, cell type, and gene ontology pathways by aggregating the risk alleles across the respective gene sets. To study this, we employed the PRSice-2 extension PRSet,[43] which performs LD clumping in a region set-aware manner, generating PRSs that preferentially include SNPs falling into regions of interest. We used the GWAS summary statistics from the CARDIoGRAMplusC4D[44] study as the basis for PRS generation, and we used the UK Biobank cohort (21,600 CAD cases and 359,254 controls) to evaluate PRS performance.

We first applied PRSet to cell state marker gene sets (gene body with −35 kb and +10 kb flanks). To evaluate PRS performance, PRSet computes the amount of phenotypic variance explained by the PRS, defined as the increase in model R^2 when PRS is included, compared to a null model consisting of covariates only. Further, permutation-based significance testing is carried out, comparing the performance for a PRS to identically clumped SNP sets from background regions.[43] We observed that PRS derived from the Vcam1+ SMC state genes explained greatest CAD risk variance and was strongly enriched in the predictive power relative to background, followed by Col2a1+ SMCs and Lrg1+ ECs (Figure 7A). Importantly, the cell state genes outperformed the corresponding cell type marker genes in predictive performance (Figure S21). Truncating the cell state marker gene sets to a specific number of top genes revealed that performance does tend to increase with the number of genes; however, the increase appears more rapid for some cell states than others, and permutation-based p value (i.e., predictive power relative to background SNP sets) tends to plateau for several cell states (Figure S22).

To investigate how marker genes of non-plaque cell types perform in a similar analysis, we derived marker sets (Table S5) for 79 human cell types across the body by using the gene expression profiles compiled from scRNA-seq datasets.[59] As an evaluation of the marker sets, related cell types showed more pairwise shared markers (Figure S23), as expected, and testing the 79 cell type marker sets (top 500 genes each) for overlap with CAD-GWAS-prioritized genes revealed the strongest enrichment in SMC and EC, followed by FB and adipocytes, while most cell types showed no significant enrichment (Figure S24). Next, we constructed gene set-based PRSs for CAD by using between 20 and 500 top markers for each cell type (gene body regions + flanks) and evaluated PRS R^2. Out of the 79 cell types, hepatocytes ranked highest, followed by SMCs, with adipocytes and ECs also among the top ten cell types (Figure S25). Several other cell types in the top ten tend to be ones that share markers with SMCs (Figure S23).

Pathway-based PRSs have been suggested to better inform disease biology compared to classical PRSs.[43,58] We therefore also performed PRS analysis on the gene ontologies that were significantly enriched among the cell state signature genes (Figures 2C and 2D). For each of the significant ontology categories (Figures 2C and 2D), we collected all human genes assigned to the ontology and used these to create a CAD PRS. Interestingly, the PRSs derived from functional categories that were shared among several cell states (Figures 2C and 2D), including cholesterol transport, lipid localization, blood vessel development, and angiogenesis gene sets, were among the top in predictive performance and enrichment significance (Figure 7B). In addition, enzyme-linked and growth factor receptor signaling, cell division, leukocyte differentiation, and cell cycle, were among the top ten categories associated with CAD risk. Altogether, our analysis suggests pivotal roles for disease-associated cell states and pathways as mediators of the genetic risk for CAD.

A great majority of the genetic variants associated with CAD are located within non-coding elements of the genome where they are thought to play a role in gene expression regulation. In line with this, inclusion of functional annotations of genomic and epigenomic elements has been shown to improve the prediction accuracy of PRSs.[60] Therefore, we derived a next set of PRSs prioritizing for variants in plaque cell type regulatory elements, represented by scATAC-seq peaks.[11] Using scATAC peaks in range TSS ± 500 kb for cell state marker genes resulted in PRSs consisting of similar numbers of variants as the gene coordinate-based PRSs above, allowing direct comparison of power. Notably, PRS performance was considerably improved when we used the regulatory elements as functional priors, with ~2-fold increase in R^2 (Figures 7A versus 7C). In line with gene coordinate-based analysis, Vcam1+ SMC state gene continued to outperform other cell states.

As the regulatory element-based analysis appeared to inform SNP selection, we further tested scATAC peak-based

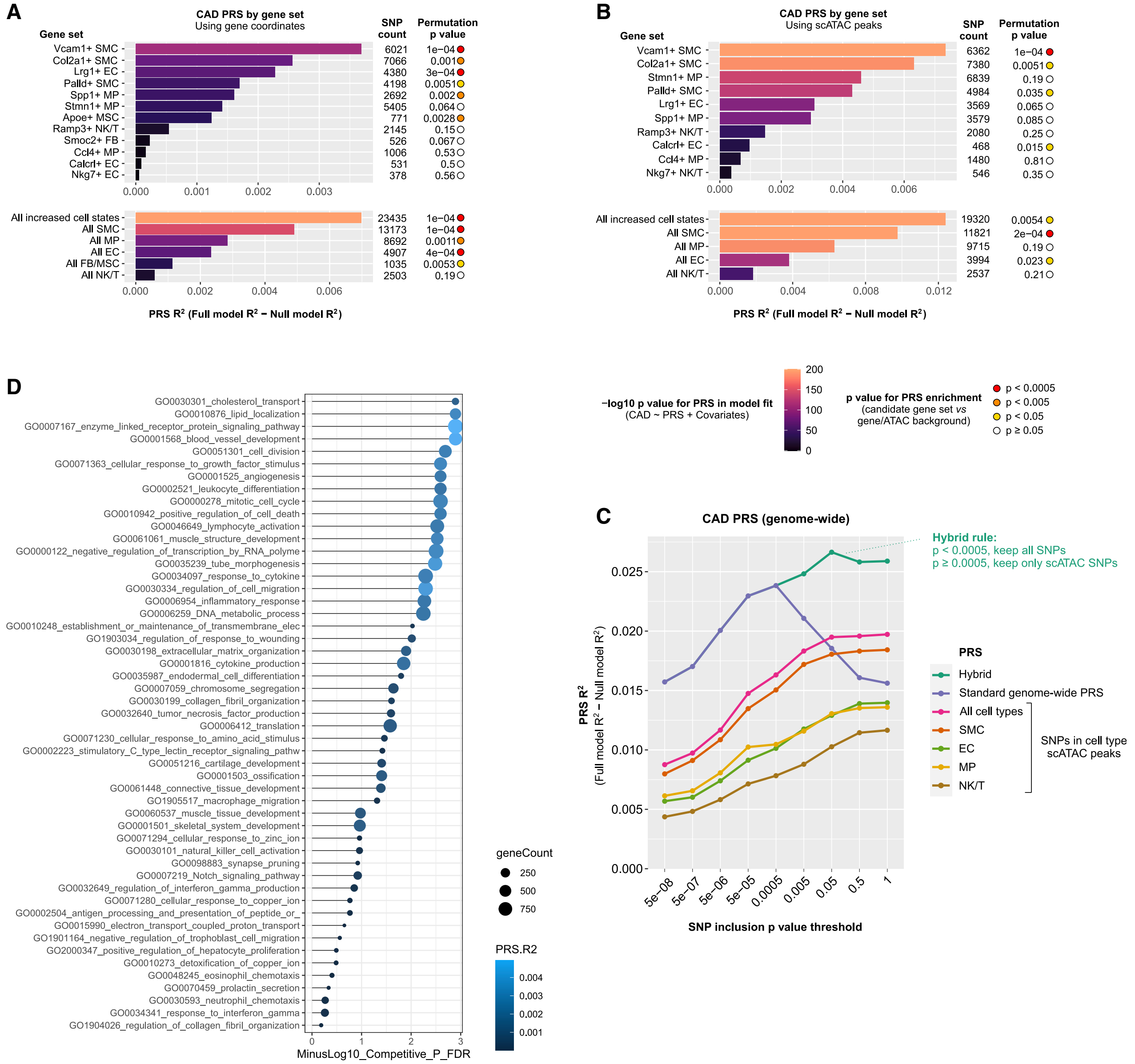

Figure 7. Pathway- and cell-state-specific polygenic risk scores shed light into the genetic basis of CAD

(A) Cell-state-specific PRS was constructed with (A) gene coordinates (−35 kb upstream to 10 kb downstream) using PRSet.[43] To obtain the empirical p value, random SNP sets containing the same number of post-clumping SNPs were selected from background regions of the genome, selected from all genic regions.

(B) Explained variance of each pathway-specific PRS to polygenic risk of CAD calculated for the gene sets listed in Figure 3C.

(C) Cell-state-specific PRS analysis constructed with plaque scATAC-seq peak coordinates that were found within ± 500 kb of the TSS.

(D) Proportion of variance of CAD explained by PRS in genome-wide analysis. The values represent PRS calculated for all cell-type-specific scATAC-seq peaks at different p value thresholds, which are compared to the classical genome-wide clumping and thresholding PRS.

PRSs genome wide (i.e., without gene set limitations). Out of the cell types studied, SMC scATAC-based PRS performed the best, although the combined PRS from all plaque scATAC cell types outperformed it (Figure 7C). This was not dependent on the total peak counts, as selecting the strongest 10,000 cell type unique peaks reproduced similar results (Figure S26). At its optimal p value threshold, the standard (classical) genome-wide PRS outperformed the scATAC PRSs. However, the p value threshold curves for scATAC and standard PRSs were differently shaped, and the scATAC PRS continued to gain power even at weaker p value thresholds whereas the standard PRS lost power (Figure 7D). Based on this observation, we constructed a hybrid PRS where all strong p value SNPs were included irrespective of scATAC data, and scATAC was only used for weaker p value variants. This hybrid PRS outperformed the classical PRS at its optimal p value threshold (Figure 7D).

Because other tissues such as liver and adipose tissue have been associated with the risk of CAD, we also generated genome region-based PRSs for CAD by using the recent scATAC atlas of 30 adult human tissues, consisting

of 111 cell types and approximately 890,000 peaks.[61] To confirm our cell type marker peak selection, we generated a pairwise marker sharing matrix, which revealed the expected similarity patterns between related cell types (Figure S27). In line with our plaque scATAC-based PRS analysis, SMC-related cell types explained the greatest CAD risk variance, followed by adipocytes, ECs, and fibroblasts. Notably, cardiomyocytes, hepatocytes, and immune cells scored considerably lower in R^2-based ranking irrespective of the total peak count used (peaks selected by ATAC signal strength; Figures S28 and 29). The cell type ranking was similar for peaks selected by cell type specificity (Figure S30).

Discussion

By performing a cell-type-unbiased time-course analysis of single cell transcriptomes in mouse atherosclerotic aorta, we identified 12 disease-associated cell states. Majority of these states are concordant with the previous studies focusing on specific subtypes of cells and lineage-tracing experiments.[1–8,53,54] In addition, we identified four less abundant disease states corresponding to Calcrl+ ECs, Smoc2+ FBs, Apoe+ MSCs, and Palld+ SMCs that require further experimental validation. Specifically, these cell states were not activated in *Ldlr*$^{-/-}$ and *Apoe*$^{-/-}$ mouse models to the same extent and could also represent states that are specific to certain aortic vascular beds. Still, our enrichment-free setup allowed us to provide the first comparison of shared and differential gene regulatory mechanisms underlying the atherosclerosis-associated cell states. Despite that the majority of marker genes were specific to a disease state, a handful of genes were shared between five or more states and thus represent potential candidates for global biomarkers of CAD. To this end, circulating levels of LGMN, MGP, SDC4, and IGFBP4 have been associated with atherosclerosis and acute cardiovascular events with potential prognostic or risk stratification value.[62–66] For example, levels of MGP and IGFBP4 could reflect differential vascular calcification burden and highlight differences in plaque pathobiology between ST-segment-elevation myocardial infarction (STEMI) and non-ST segment elevation myocardial infarction (NSTEMI).[66]

Signals from the microenvironment can be transmitted into the intracellular gene expression programs through multiple layers of signal propagation including ligand-receptor interactions, signaling molecules, and transcription factors. Based on the predicted ligand-receptor activities and downstream gene expression changes, our analysis strongly suggests that TGFβ, IL-1α/β, TNF, VEGFA, FGF1/2, APOE, and SPP1 signaling pathways dominate in the atherosclerotic microenvironment and are shared as upstream inducers of many cell states. This suggests that the distinct gene content of the disease-associated gene signatures is a result of cell-type-specific responses to the same environmental stimuli. This is in line with the recent report by us and others where proatherogenic stimulus-induced gene expression responses in ECs, SMCs, and MPs appeared very cell type specific.[67,68] Supporting this, we demonstrate that cell states are largely governed by specific TFs that form intricate gene regulatory networks. Still, a few TF modules were shared between the SMC and EC cell states (XBP1, TCF4, CEBPB, CREB3L2, and ELK3) and between SMC and MP cell states (XBP1, MAFB, MLX, and NRF2), identifying them as potential candidates driving the expression of the genes in shared ontology categories related to ECM organization, blood vessel development, angiogenesis, cholesterol transport, and lipid localization. Indeed, XBP1, a key modulator of unfolded protein response, has been demonstrated to play an important role in the regulation of lipid metabolism and angiogenesis and the inhibition of this pathway alleviates atherosclerosis.[57,69,70]

Analysis of the atherosclerotic cell state signatures in human lesions supported the relevance of the majority of the cell states in separating healthy from diseased samples or early disease from advanced disease samples. But in cases where the cell state gene sets were short, the power of sample classification was not apparent. Supporting the relevance of the disease signatures, we further demonstrated that many of the cell state marker genes were predicted target genes of CAD GWAS variants and the genome regions where the marker genes reside contribute significantly to the heritability of CAD. Importantly, the genetic variation in the Vcam1+ SMCs and Col2a1+ SMCs contributed to CAD heritability beyond other cell states, suggesting that these cell states are particularly important in understanding the pathobiology of atherosclerosis. This is also supported by other recent studies looking into the cell-type-specific expression of predicted GWAS target genes.[71,72] Importantly, our analysis demonstrated that atherosclerosis-associated cell state markers explain a larger proportion of CAD risk variance compared to cell type markers, suggesting a more prominent role for genes that participate in pathological changes compared to those that maintain healthy cell identity.

Substantial ongoing efforts are looking into applying CAD PRS in the clinical practice including risk stratification and prediction of treatment response. By enhancing early prediction of CAD beyond traditional risk factors, PRS could also guide new treatment strategies. This is exemplified by several landmark studies that demonstrated that the individuals at high genetic risk for CAD experience the greatest benefit from lipid-lowering treatments.[73–75] However, the standard PRS sums an individual's genetic profile to a single estimate that may fail to identify more nuanced phenotypes that are necessary for risk stratification, prediction of treatment response, or identification of pathways leading to novel treatment.[43] Here, we evaluated the performance of PRS, which accounts for genomic substructure/regional functional heterogeneity by aggregating risk alleles across cell states/types and biological pathways. Our results demonstrate that while the regular (genome-wide) PRS outperforms the cell state gene set-based PRSs in absolute

accuracy, regulatory element-based PRS effectively quality filters the SNPs with weaker GWAS p value. This allowed us to construct a hybrid PRS that achieved higher power than the regular PRS at its optimal p value threshold. On the other hand, pathway-based PRS identified biological processes conferring higher genetic risk, providing pathway-level processes to target in drug design. In particular, the pathways shared between several cell states related to cholesterol transport, lipid localization, extracellular matrix, blood vessel development, and angiogenesis could provide actionable targets. While our analysis provides the first steps toward cell-state-, cell-type-, and pathway-level understanding of the genetic risk, future studies are needed to evaluate whether incorporation of such functional prior information improves polygenic prediction accuracy in individual stratification or prediction of treatment response.

In summary, we provide in depth characterization of atherosclerosis-associated cell states and demonstrate the value of cell-state-specific markers in understanding the genetic basis of CAD. Substantial work still needs to be done to functionally validate the role of these genes in the pathophysiology of atherosclerosis. Defining the mechanisms that contribute to distinct cell states in pathological conditions could provide a basis for applying precision medicine and targeted therapies in the future.

Data and code availability

The accession number for the mouse bulk RNA-seq and scRNA-seq reported in this paper is GEO: GSE205929 and GSE205930. The MOVAS count matrices are available at FigShare DOI: https://doi.org/10.6084/m9.figshare.20059649.v1. The Maastricht Pathology Tissue Collection data were downloaded under GEO accession number GEO: GSE28829. Previously published mouse and human atherosclerosis scRNA-seq datasets were obtained from GEO accessions GEO: GSE155513 and GSE131778, respectively.

Supplemental information

Supplemental information can be found online at https://doi.org/10.1016/j.ajhg.2023.03.013.

Acknowledgments

We acknowledge Single Cell Genomics Core, Genome Center and Cell and Tissue Imaging Unit at University of Eastern Finland, Finnish Functional Genomics Centre, and Biocenter Finland for infrastructure support. This research has been conducted with the UK Biobank Resource (https://www.ukbiobank.ac.uk/) under application number 58990. We are grateful to Resolve Biosciences for their excellent service in optimizing and performing the Molecular Cartography experiments. This study was funded by the European Research Council (ERC) under the European Union's Horizon 2020 research and innovation program (grant no. 802825 to M.U.K.), Academy of Finland (grant no. 333021 and 335973 to M.U.K.; 311081 and 314557 to T. Lönnberg; 349708 to P.P.M.; 314556 and 335975 to A. Roivainen; 321535 and 328835 to J.P.L.), InFLAMES Flagship Programme of the Academy of Finland (337530 to T. Lönnberg), Finnish Foundation for Cardiovascular Research (M.U.K., P.R.M., and J.P.L.), Instrumentarium Science Foundation (P.R.M.), Ida Montinin Foundation (P.R.M.), Sigrid Jusélius Foundation (M.U.K.), GenomMed doctoral program (740264 to A. Ravindran), and Jane and Aatos Erkko Foundation (A. Roivainen). The Tampere Vascular Study (TVS) was supported with grants from the Competitive Research Funding of the Tampere University Hospital (grants 9M048 and 9N035 to T. Lehtimäki), the Emil Aaltonen Foundation (to T. Lehtimäki), the Pirkanmaa Regional Fund of the Finnish Cultural Foundation, the Research Foundation of Orion Corporation, the Jenny and Antti Wihuri Foundation, and the Academy of Finland (grants 322098 and 104821), the Finnish Foundation for Cardiovascular Research, the Yrjö Jahnsson Foundation, European Union 7th Framework Program (grant 201668 for AtheroRemo), EU Horizon 2020 (grant 755320 for TAXINOMISIS and grant 848146 for To Aition), and the Academy of Finland grant 322098 and Tampere University Hospital Supporting Foundation.

Declaration of interests

The authors declare no competing interests.

Received: December 22, 2022
Accepted: March 21, 2023
Published: April 14, 2023

References

1. Zernecke, A., Winkels, H., Cochain, C., Williams, J.W., Wolf, D., Soehnlein, O., Robbins, C.S., Monaco, C., Park, I., McNamara, C.A., et al. (2020). Meta-Analysis of Leukocyte Diversity in Atherosclerotic Mouse Aortas. Circ. Res. *127*, 402–426.
2. Dobnikar, L., Taylor, A.L., Chappell, J., Oldach, P., Harman, J.L., Oerton, E., Dzierzak, E., Bennett, M.R., Spivakov, M., and Jørgensen, H.F. (2018). Disease-relevant transcriptional signatures identified in individual smooth muscle cells from healthy mouse vessels. Nat. Commun. *9*, 4567.
3. Wirka, R.C., Wagh, D., Paik, D.T., Pjanic, M., Nguyen, T., Miller, C.L., Kundu, R., Nagao, M., Coller, J., Koyano, T.K., et al. (2019). Atheroprotective roles of smooth muscle cell phenotypic modulation and the TCF21 disease gene as revealed by single-cell analysis. Nat. Med. *25*, 1280–1289.
4. Pan, H., Xue, C., Auerbach, B.J., Fan, J., Bashore, A.C., Cui, J., Yang, D.Y., Trignano, S.B., Liu, W., Shi, J., et al. (2020). Single-Cell Genomics Reveals a Novel Cell State During Smooth Muscle Cell Phenotypic Switching and Potential Therapeutic Targets for Atherosclerosis in Mouse and Human. Circulation *142*, 2060–2075.
5. Alencar, G.F., Owsiany, K.M., Karnewar, S., Sukhavasi, K., Mocci, G., Nguyen, A.T., Williams, C.M., Shamsuzzaman, S., Mokry, M., Henderson, C.A., et al. (2020). Stem Cell Pluripotency Genes Klf4 and Oct4 Regulate Complex SMC Phenotypic Changes Critical in Late-Stage Atherosclerotic Lesion Pathogenesis. Circulation *142*, 2045–2059.
6. Kim, J.B., Zhao, Q., Nguyen, T., Pjanic, M., Cheng, P., Wirka, R., Travisano, S., Nagao, M., Kundu, R., and Quertermous, T. (2020). Environment-Sensing Aryl Hydrocarbon Receptor

Inhibits the Chondrogenic Fate of Modulated Smooth Muscle Cells in Atherosclerotic Lesions. Circulation *142*, 575–590.
7. Andueza, A., Kumar, S., Kim, J., Kang, D.W., Mumme, H.L., Perez, J.I., Villa-Roel, N., and Jo, H. (2020). Endothelial Reprogramming by Disturbed Flow Revealed by Single-Cell RNA and Chromatin Accessibility Study. Cell Rep. *33*, 108491.
8. Zhao, G., Lu, H., Liu, Y., Zhao, Y., Zhu, T., Garcia-Barrio, M.T., Chen, Y.E., and Zhang, J. (2021). Single-Cell Transcriptomics Reveals Endothelial Plasticity During Diabetic Atherogenesis. Front. Cell Dev. Biol. *9*, 689469.
9. Aragam, K.G., Jiang, T., Goel, A., Kanoni, S., Wolford, B.N., Atri, D.S., Weeks, E.M., Wang, M., Hindy, G., Zhou, W., et al. (2022). Discovery and systematic characterization of risk variants and genes for coronary artery disease in over a million participants. Nat. Genet. *54*, 1803–1815.
10. Tcheandjieu, C., Zhu, X., Hilliard, A.T., Clarke, S.L., Napolioni, V., Ma, S., Lee, K.M., Fang, H., Chen, F., Lu, Y., et al. (2022). Large-scale genome-wide association study of coronary artery disease in genetically diverse populations. Nat. Med. *28*, 1679–1692.
11. Örd, T., Õunap, K., Stolze, L.K., Aherrahrou, R., Nurminen, V., Toropainen, A., Selvarajan, I., Lönnberg, T., Aavik, E., Ylä-Herttuala, S., et al. (2021). Single-Cell Epigenomics and Functional Fine-Mapping of Atherosclerosis GWAS Loci. Circ. Res. *129*, 240–258.
12. Powell-Braxton, L., Véniant, M., Latvala, R.D., Hirano, K.I., Won, W.B., Ross, J., Dybdal, N., Zlot, C.H., Young, S.G., and Davidson, N.O. (1998). A mouse model of human familial hypercholesterolemia: markedly elevated low density lipoprotein cholesterol levels and severe atherosclerosis on a low-fat chow diet. Nat. Med. *4*, 934–938.
13. Farese, R.V., Jr., Véniant, M.M., Cham, C.M., Flynn, L.M., Pierotti, V., Loring, J.F., Traber, M., Ruland, S., Stokowski, R.S., Huszar, D., and Young, S.G. (1996). Phenotypic analysis of mice expressing exclusively apolipoprotein B48 or apolipoprotein B100. Proc. Natl. Acad. Sci. USA *93*, 6393–6398.
14. Stuart, T., Butler, A., Hoffman, P., Hafemeister, C., Papalexi, E., Mauck, W.M., 3rd, Hao, Y., Stoeckius, M., Smibert, P., and Satija, R. (2019). Comprehensive Integration of Single-Cell Data. Cell *177*, 1888–1902.e21.
15. Yang, S., Corbett, S.E., Koga, Y., Wang, Z., Johnson, W.E., Yajima, M., and Campbell, J.D. (2020). Decontamination of ambient RNA in single-cell RNA-seq with DecontX. Genome Biol. *21*, 57.
16. Raudvere, U., Kolberg, L., Kuzmin, I., Arak, T., Adler, P., Peterson, H., and Vilo, J. (2019). g:Profiler: a web server for functional enrichment analysis and conversions of gene lists (2019 update). Nucleic Acids Res. *47*, W191–W198.
17. Yu, G. (2020). Gene Ontology Semantic Similarity Analysis Using GOSemSim. Methods Mol. Biol. *2117*, 207–215.
18. DeTomaso, D., Jones, M.G., Subramaniam, M., Ashuach, T., Ye, C.J., and Yosef, N. (2019). Functional interpretation of single cell similarity maps. Nat. Commun. *10*, 4376.
19. Liberzon, A., Birger, C., Thorvaldsdóttir, H., Ghandi, M., Mesirov, J.P., and Tamayo, P. (2015). The Molecular Signatures Database (MSigDB) hallmark gene set collection. Cell Syst. *1*, 417–425.
20. Browaeys, R., Saelens, W., and Saeys, Y. (2020). NicheNet: modeling intercellular communication by linking ligands to target genes. Nat. Methods *17*, 159–162.
21. Aibar, S., González-Blas, C.B., Moerman, T., Huynh-Thu, V.A., Imrichova, H., Hulselmans, G., Rambow, F., Marine, J.C., Geurts, P., Aerts, J., et al. (2017). SCENIC: single-cell regulatory network inference and clustering. Nat. Methods *14*, 1083–1086.
22. Dobin, A., Davis, C.A., Schlesinger, F., Drenkow, J., Zaleski, C., Jha, S., Batut, P., Chaisson, M., and Gingeras, T.R. (2013). STAR: ultrafast universal RNA-seq aligner. Bioinformatics *29*, 15–21.
23. Heinz, S., Benner, C., Spann, N., Bertolino, E., Lin, Y.C., Laslo, P., Cheng, J.X., Murre, C., Singh, H., and Glass, C.K. (2010). Simple Combinations of Lineage-Determining Transcription Factors Prime cis-Regulatory Elements Required for Macrophage and B Cell Identities. Mol. Cell *38*, 576–589.
24. Frishberg, A., Peshes-Yaloz, N., Cohn, O., Rosentul, D., Steuerman, Y., Valadarsky, L., Yankovitz, G., Mandelboim, M., Iraqi, F.A., Amit, I., et al. (2019). Cell composition analysis of bulk genomics using single-cell data. Nat. Methods *16*, 327–332.
25. Sulkava, M., Raitoharju, E., Levula, M., Seppälä, I., Lyytikäinen, L.P., Mennander, A., Järvinen, O., Zeitlin, R., Salenius, J.P., Illig, T., et al. (2017). Differentially expressed genes and canonical pathway expression in human atherosclerotic plaques - Tampere Vascular Study. Sci. Rep. *7*, 41483.
26. Döring, Y., Manthey, H.D., Drechsler, M., Lievens, D., Megens, R.T.A., Soehnlein, O., Busch, M., Manca, M., Koenen, R.R., Pelisek, J., et al. (2012). Auto-antigenic protein-DNA complexes stimulate plasmacytoid dendritic cells to promote atherosclerosis. Circulation *125*, 1673–1683.
27. Tirosh, I., Izar, B., Prakadan, S.M., Wadsworth, M.H., 2nd, Treacy, D., Trombetta, J.J., Rotem, A., Rodman, C., Lian, C., Murphy, G., et al. (2016). Dissecting the multicellular ecosystem of metastatic melanoma by single-cell RNA-seq. Science *352*, 189–196.
28. Mountjoy, E., Schmidt, E.M., Carmona, M., Schwartzentruber, J., Peat, G., Miranda, A., Fumis, L., Hayhurst, J., Buniello, A., Karim, M.A., et al. (2021). An open approach to systematically prioritize causal variants and genes at all published human GWAS trait-associated loci. Nat. Genet. *53*, 1527–1533.
29. van der Harst, P., and Verweij, N. (2018). Identification of 64 Novel Genetic Loci Provides an Expanded View on the Genetic Architecture of Coronary Artery Disease. Circ. Res. *122*, 433–443.
30. Nelson, C.P., Goel, A., Butterworth, A.S., Kanoni, S., Webb, T.R., Marouli, E., Zeng, L., Ntalla, I., Lai, F.Y., Hopewell, J.C., et al. (2017). Association analyses based on false discovery rate implicate new loci for coronary artery disease. Nat. Genet. *49*, 1385–1391.
31. Malik, R., Chauhan, G., Traylor, M., Sargurupremraj, M., Okada, Y., Mishra, A., Rutten-Jacobs, L., Giese, A.K., van der Laan, S.W., Gretarsdottir, S., et al. (2018). Multiancestry genome-wide association study of 520,000 subjects identifies 32 loci associated with stroke and stroke subtypes. Nat. Genet. *50*, 524–537.
32. Kichaev, G., Bhatia, G., Loh, P.R., Gazal, S., Burch, K., Freund, M.K., Schoech, A., Pasaniuc, B., and Price, A.L. (2019). Leveraging Polygenic Functional Enrichment to Improve GWAS Power. Am. J. Hum. Genet. *104*, 65–75.
33. Matsunaga, H., Ito, K., Akiyama, M., Takahashi, A., Koyama, S., Nomura, S., Ieki, H., Ozaki, K., Onouchi, Y., Sakaue, S., et al. (2020). Transethnic Meta-Analysis of Genome-Wide Association Studies Identifies Three New Loci and Characterizes Population-Specific Differences for Coronary Artery Disease. Circ. Genom. Precis. Med. *13*, e002670.

34. Koyama, S., Ito, K., Terao, C., Akiyama, M., Horikoshi, M., Momozawa, Y., Matsunaga, H., Ieki, H., Ozaki, K., Onouchi, Y., et al. (2020). Population-specific and trans-ancestry genome-wide analyses identify distinct and shared genetic risk loci for coronary artery disease. Nat. Genet. *52*, 1169–1177.
35. Hartiala, J.A., Han, Y., Jia, Q., Hilser, J.R., Huang, P., Gukasyan, J., Schwartzman, W.S., Cai, Z., Biswas, S., Trégouët, D.A., et al. (2021). Genome-wide analysis identifies novel susceptibility loci for myocardial infarction. Eur. Heart J. *42*, 919–933.
36. Zhou, W., Nielsen, J.B., Fritsche, L.G., Dey, R., Gabrielsen, M.E., Wolford, B.N., LeFaive, J., VandeHaar, P., Gagliano, S.A., Gifford, A., et al. (2018). Efficiently controlling for case-control imbalance and sample relatedness in large-scale genetic association studies. Nat. Genet. *50*, 1335–1341.
37. Kurki, M.I., Karjalainen, J., Palta, P., Sipilä, T.P., Kristiansson, K., Donner, K., Reeve, M.P., Laivuori, H., Aavikko, M., Kaunisto, M.A., et al. (2023). FinnGen provides genetic insights from a well-phenotyped isolated population. Nature. *613*, 508–518.
38. Li, L., Chen, Z., von Scheidt, M., Li, S., Steiner, A., Güldener, U., Koplev, S., Ma, A., Hao, K., Pan, C., et al. (2022). Transcriptome-wide association study of coronary artery disease identifies novel susceptibility genes. Basic Res. Cardiol. *117*, 6.
39. Erdmann, J., Kessler, T., Munoz Venegas, L., and Schunkert, H. (2018). A decade of genome-wide association studies for coronary artery disease: the challenges ahead. Cardiovasc. Res. *114*, 1241–1257.
40. Finucane, H.K., Bulik-Sullivan, B., Gusev, A., Trynka, G., Reshef, Y., Loh, P.R., Anttila, V., Xu, H., Zang, C., Farh, K., et al. (2015). Partitioning heritability by functional annotation using genome-wide association summary statistics. Nat. Genet. *47*, 1228–1235.
41. Zhang, Y., Liu, T., Meyer, C.A., Eeckhoute, J., Johnson, D.S., Bernstein, B.E., Nusbaum, C., Myers, R.M., Brown, M., Li, W., and Liu, X.S. (2008). Model-based analysis of ChIP-Seq (MACS). Genome Biol. *9*, R137.
42. Choi, S.W., and O'Reilly, P.F. (2019). PRSice-2: Polygenic Risk Score software for biobank-scale data. GigaScience *8*, giz082.
43. Choi, S.W., Garcia-Gonzalez, J., Ruan, Y., Wu, H.M., Porras, C., Johnson, J., Bipolar Disorder Working group of the Psychiatric Genomics Consortium, Hoggart, C.J., and O'Reilly, P.F. (2023). PRSet: Pathway-based polygenic risk score analyses and software. PLoS Genet. *19*, e1010624.
44. Nikpay, M., Goel, A., Won, H.H., Hall, L.M., Willenborg, C., Kanoni, S., Saleheen, D., Kyriakou, T., Nelson, C.P., Hopewell, J.C., et al. (2015). A comprehensive 1,000 Genomes-based genome-wide association meta-analysis of coronary artery disease. Nat. Genet. *47*, 1121–1130.
45. Bycroft, C., Freeman, C., Petkova, D., Band, G., Elliott, L.T., Sharp, K., Motyer, A., Vukcevic, D., Delaneau, O., O'Connell, J., et al. (2018). The UK Biobank resource with deep phenotyping and genomic data. Nature *562*, 203–209.
46. Chang, C.C., Chow, C.C., Tellier, L.C., Vattikuti, S., Purcell, S.M., and Lee, J.J. (2015). Second-generation PLINK: rising to the challenge of larger and richer datasets. GigaScience *4*, 7.
47. Dann, E., Henderson, N.C., Teichmann, S.A., Morgan, M.D., and Marioni, J.C. (2022). Differential abundance testing on single-cell data using k-nearest neighbor graphs. Nat. Biotechnol. *40*, 245–253.
48. Denisenko, E., Guo, B.B., Jones, M., Hou, R., de Kock, L., Lassmann, T., Poppe, D., Clément, O., Simmons, R.K., Lister, R., and Forrest, A.R.R. (2020). Systematic assessment of tissue dissociation and storage biases in single-cell and single-nucleus RNA-seq workflows. Genome Biol. *21*, 130.
49. Newman, A.M., Steen, C.B., Liu, C.L., Gentles, A.J., Chaudhuri, A.A., Scherer, F., Khodadoust, M.S., Esfahani, M.S., Luca, B.A., Steiner, D., et al. (2019). Determining cell type abundance and expression from bulk tissues with digital cytometry. Nat. Biotechnol. *37*, 773–782.
50. Cheng, C.K., Lin, X., Pu, Y., Tse, J.K.Y., Wang, Y., Zhang, C.-L., Cao, X., Lau, C.W., Huang, J., He, L., et al. (2022). SOX4 is a novel phenotypic regulator of endothelial cells in atherosclerosis revealed by single-cell analysis. J. Adv. Res. *43*, 187–203.
51. Sobierajska, K., Wawro, M.E., Ciszewski, W.M., and Niewiarowska, J. (2019). Transforming Growth Factor-beta Receptor Internalization via Caveolae Is Regulated by Tubulin-beta2 and Tubulin-beta3 during Endothelial-Mesenchymal Transition. Am. J. Pathol. *189*, 2531–2546.
52. Lee, Y.H., Albig, A.R., Regner, M., Schiemann, B.J., and Schiemann, W.P. (2008). Fibulin-5 initiates epithelial-mesenchymal transition (EMT) and enhances EMT induced by TGF-beta in mammary epithelial cells via a MMP-dependent mechanism. Carcinogenesis *29*, 2243–2251.
53. Winkels, H., and Wolf, D. (2021). Heterogeneity of T Cells in Atherosclerosis Defined by Single-Cell RNA-Sequencing and Cytometry by Time of Flight. Arterioscler. Thromb. Vasc. Biol. *41*, 549–563.
54. Fernandez, D.M., Rahman, A.H., Fernandez, N.F., Chudnovskiy, A., Amir, E.A.D., Amadori, L., Khan, N.S., Wong, C.K., Shamailova, R., Hill, C.A., et al. (2019). Single-cell immune landscape of human atherosclerotic plaques. Nat. Med. *25*, 1576–1588.
55. Sampieri, L., Di Giusto, P., and Alvarez, C. (2019). CREB3 Transcription Factors: ER-Golgi Stress Transducers as Hubs for Cellular Homeostasis. Front. Cell Dev. Biol. *7*, 123.
56. Laurent, F., Girdziusaite, A., Gamart, J., Barozzi, I., Osterwalder, M., Akiyama, J.A., Lincoln, J., Lopez-Rios, J., Visel, A., Zuniga, A., and Zeller, R. (2017). HAND2 Target Gene Regulatory Networks Control Atrioventricular Canal and Cardiac Valve Development. Cell Rep. *19*, 1602–1613.
57. Moncan, M., Mnich, K., Blomme, A., Almanza, A., Samali, A., and Gorman, A.M. (2021). Regulation of lipid metabolism by the unfolded protein response. J. Cell Mol. Med. *25*, 1359–1370.
58. Klarin, D., and Natarajan, P. (2022). Clinical utility of polygenic risk scores for coronary artery disease. Nat. Rev. Cardiol. *19*, 291–301.
59. Karlsson, M., Zhang, C., Méar, L., Zhong, W., Digre, A., Katona, B., Sjöstedt, E., Butler, L., Odeberg, J., Dusart, P., et al. (2021). A single-cell type transcriptomics map of human tissues. Sci. Adv. *7*, eabh2169.
60. Hu, Y., Lu, Q., Powles, R., Yao, X., Yang, C., Fang, F., Xu, X., and Zhao, H. (2017). Leveraging functional annotations in genetic risk prediction for human complex diseases. PLoS Comput. Biol. *13*, e1005589.
61. Zhang, K., Hocker, J.D., Miller, M., Hou, X., Chiou, J., Poirion, O.B., Qiu, Y., Li, Y.E., Gaulton, K.J., Wang, A., et al. (2021). A single-cell atlas of chromatin accessibility in the human genome. Cell *184*, 5985–6001.e19.
62. Lunde, N.N., Holm, S., Dahl, T.B., Elyouncha, I., Sporsheim, B., Gregersen, I., Abbas, A., Skjelland, M., Espevik, T., Solberg, R., et al. (2017). Increased levels of legumain in plasma and plaques from patients with carotid atherosclerosis. Atherosclerosis *257*, 216–223.

63. Lunde, N.N., Gregersen, I., Ueland, T., Shetelig, C., Holm, S., Kong, X.Y., Michelsen, A.E., Otterdal, K., Yndestad, A., Broch, K., et al. (2020). Legumain is upregulated in acute cardiovascular events and associated with improved outcome - potentially related to anti-inflammatory effects on macrophages. Atherosclerosis *296*, 74–82.
64. Kumric, M., Borovac, J.A., Ticinovic Kurir, T., Martinovic, D., Frka Separovic, I., Baric, L., and Bozic, J. (2021). Role of Matrix Gla Protein in the Complex Network of Coronary Artery Disease: A Comprehensive Review. Life *11*, 737.
65. Solbu, M.D., Kolset, S.O., Jenssen, T.G., Wilsgaard, T., Løchen, M.L., Mathiesen, E.B., Melsom, T., Eriksen, B.O., and Reine, T.M. (2018). Gender differences in the association of syndecan-4 with myocardial infarction: The population-based Tromso Study. Atherosclerosis *278*, 166–173.
66. Cediel, G., Rueda, F., Oxvig, C., Oliveras, T., Labata, C., de Diego, O., Ferrer, M., Aranda-Nevado, M.C., Serra-Gregori, J., Núñez, J., et al. (2018). Prognostic value of the Stanniocalcin-2/PAPP-A/IGFBP-4 axis in ST-segment elevation myocardial infarction. Cardiovasc. Diabetol. *17*, 63.
67. Chen, P.Y., Qin, L., Li, G., Wang, Z., Dahlman, J.E., Malagon-Lopez, J., Gujja, S., Cilfone, N.A., Kauffman, K.J., Sun, L., et al. (2019). Endothelial TGF-beta signalling drives vascular inflammation and atherosclerosis. Nat. Metab. *1*, 912–926.
68. Moreau, P.R., Tomas Bosch, V., Bouvy-Liivrand, M., Õunap, K., Örd, T., Pulkkinen, H.H., Pölönen, P., Heinäniemi, M., Ylä--Herttuala, S., Laakkonen, J.P., et al. (2021). Profiling of Primary and Mature miRNA Expression in Atherosclerosis-Associated Cell Types. Arterioscler. Thromb. Vasc. Biol. *41*, 2149–2167.
69. Binet, F., and Sapieha, P. (2015). ER Stress and Angiogenesis. Cell Metab. *22*, 560–575.
70. Tufanli, O., Telkoparan Akillilar, P., Acosta-Alvear, D., Kocaturk, B., Onat, U.I., Hamid, S.M., Çimen, I., Walter, P., Weber, C., and Erbay, E. (2017). Targeting IRE1 with small molecules counteracts progression of atherosclerosis. Proc. Natl. Acad. Sci. USA *114*, E1395–E1404.
71. Slenders, L., Landsmeer, L.P.L., Cui, K., Depuydt, M.A.C., Verwer, M., Mekke, J., Timmerman, N., van den Dungen, N.A.M., Kuiper, J., de Winther, M.P.J., et al. (2022). Intersecting single-cell transcriptomics and genome-wide association studies identifies crucial cell populations and candidate genes for atherosclerosis. Eur. Heart J. Open *2*, oeab043.
72. Depuydt, M.A.C., Prange, K.H.M., Slenders, L., Örd, T., Elbersen, D., Boltjes, A., de Jager, S.C.A., Asselbergs, F.W., de Borst, G.J., Aavik, E., et al. (2020). Microanatomy of the Human Atherosclerotic Plaque by Single-Cell Transcriptomics. Circ. Res. *127*, 1437–1455.
73. Marston, N.A., Kamanu, F.K., Nordio, F., Gurmu, Y., Roselli, C., Sever, P.S., Pedersen, T.R., Keech, A.C., Wang, H., Lira Pineda, A., et al. (2020). Predicting Benefit From Evolocumab Therapy in Patients With Atherosclerotic Disease Using a Genetic Risk Score: Results From the FOURIER Trial. Circulation *141*, 616–623.
74. Damask, A., Steg, P.G., Schwartz, G.G., Szarek, M., Hagström, E., Badimon, L., Chapman, M.J., Boileau, C., Tsimikas, S., Ginsberg, H.N., et al. (2020). Patients With High Genome-Wide Polygenic Risk Scores for Coronary Artery Disease May Receive Greater Clinical Benefit From Alirocumab Treatment in the ODYSSEY OUTCOMES Trial. Circulation *141*, 624–636.
75. Mega, J.L., Stitziel, N.O., Smith, J.G., Chasman, D.I., Caulfield, M., Devlin, J.J., Nordio, F., Hyde, C., Cannon, C.P., Sacks, F., et al. (2015). Genetic risk, coronary heart disease events, and the clinical benefit of statin therapy: an analysis of primary and secondary prevention trials. Lancet *385*, 2264–2271.

The American Journal of Human Genetics, Volume *110*

Supplemental information

Dissecting the polygenic basis of atherosclerosis via disease-associated cell state signatures

Tiit Örd, Tapio Lönnberg, Valtteri Nurminen, Aarthi Ravindran, Henri Niskanen, Miika Kiema, Kadri Õunap, Maleeha Maria, Pierre R. Moreau, Pashupati P. Mishra, Senthil Palani, Jenni Virta, Heidi Liljenbäck, Einari Aavik, Anne Roivainen, Seppo Ylä-Herttuala, Johanna P. Laakkonen, Terho Lehtimäki, and Minna U. Kaikkonen

Supplemental Figures

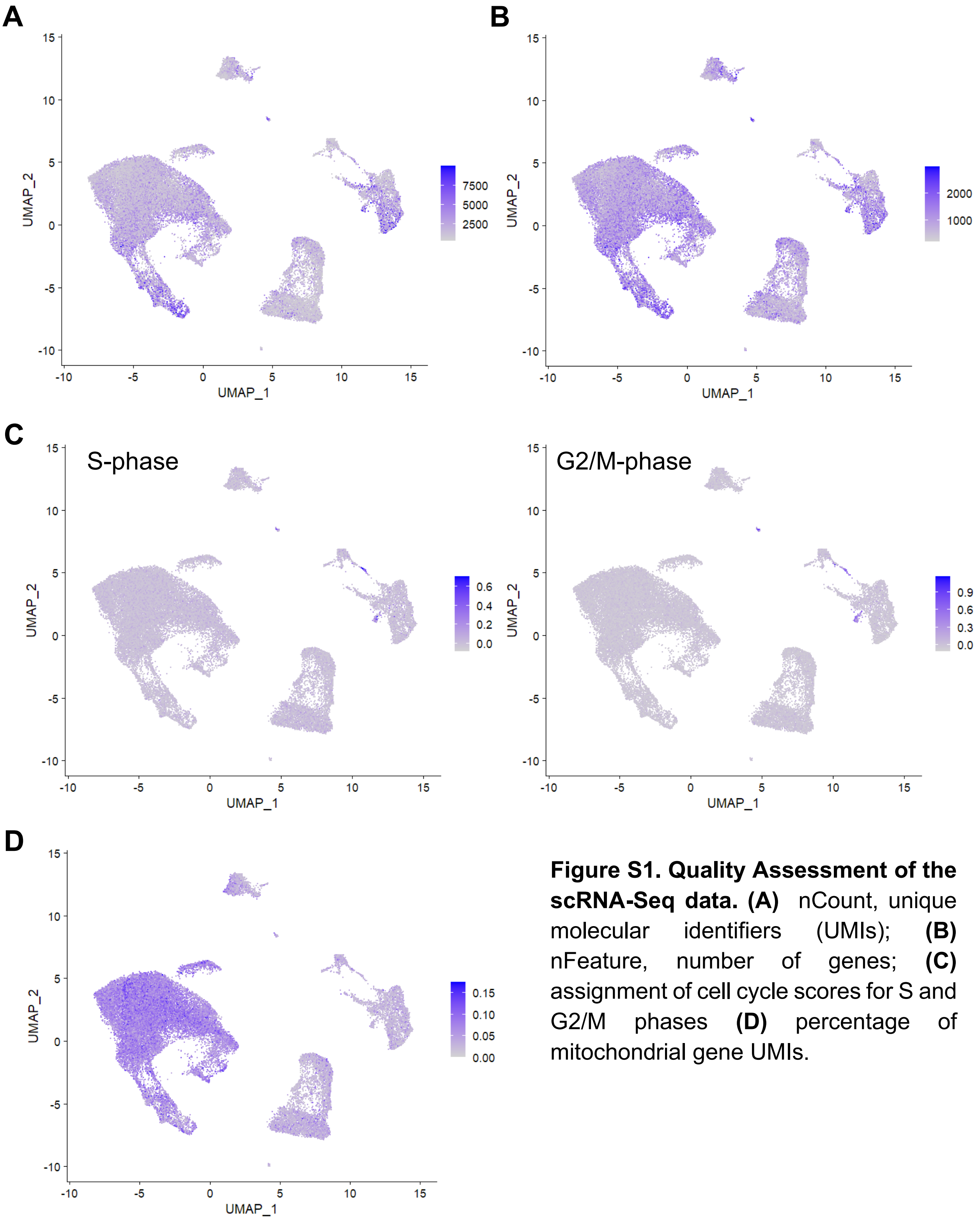

Figure S1. Quality Assessment of the scRNA-Seq data. (A) nCount, unique molecular identifiers (UMIs); **(B)** nFeature, number of genes; **(C)** assignment of cell cycle scores for S and G2/M phases **(D)** percentage of mitochondrial gene UMIs.

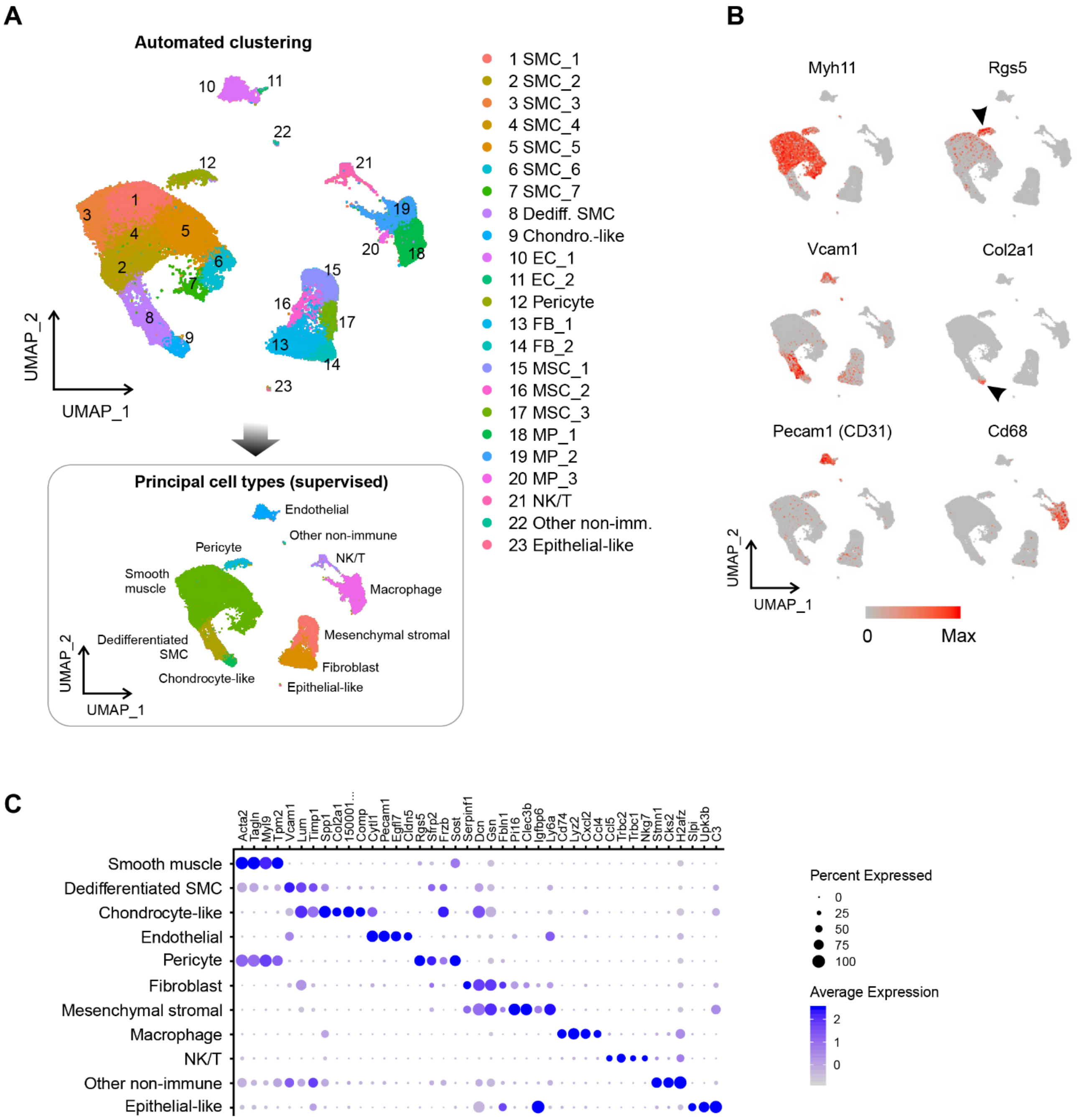

Figure S2. Clustering and identification of cell types in scRNA-Seq of mouse atherosclerotic lesion. (A) UMAP projection of the scRNA-Seq profiles represented as 23 clusters identified using automated clustering and the eleven manually annotated populations. (**B**) UMAP plots showing the expression of selected markers used to annotate the cell types. (**C**) Dot plot demonstrating the top four marker genes for each lesional cell type. Dot size corresponds to the proportion of cells within the cluster that expressed the gene, and dot color intensity corresponds to the average expression level.

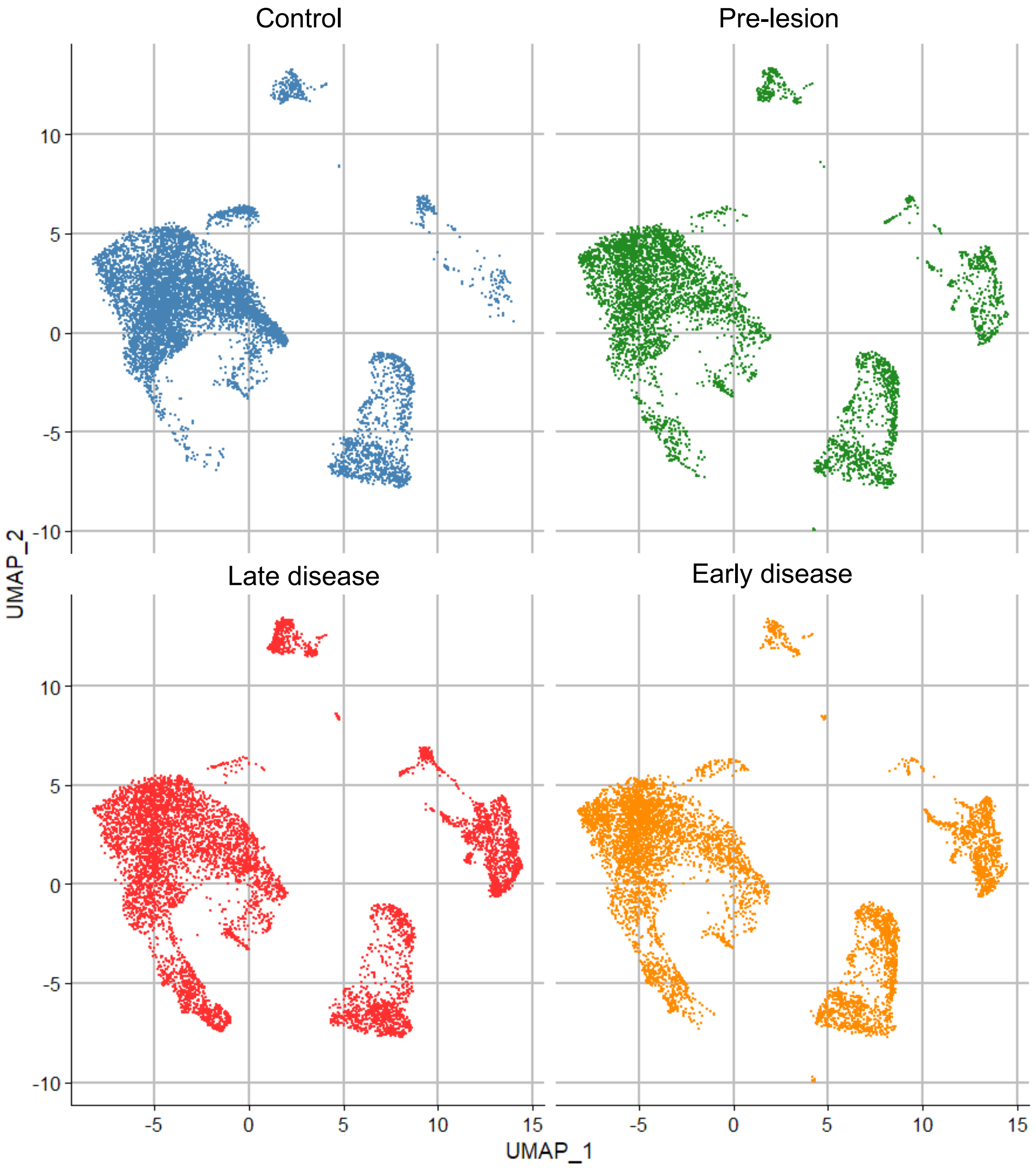

Figure S3. Changes in the cell numbers during progression of atherosclerosis. UMAP projection of the scRNA-Seq profiles separately for each disease condition.

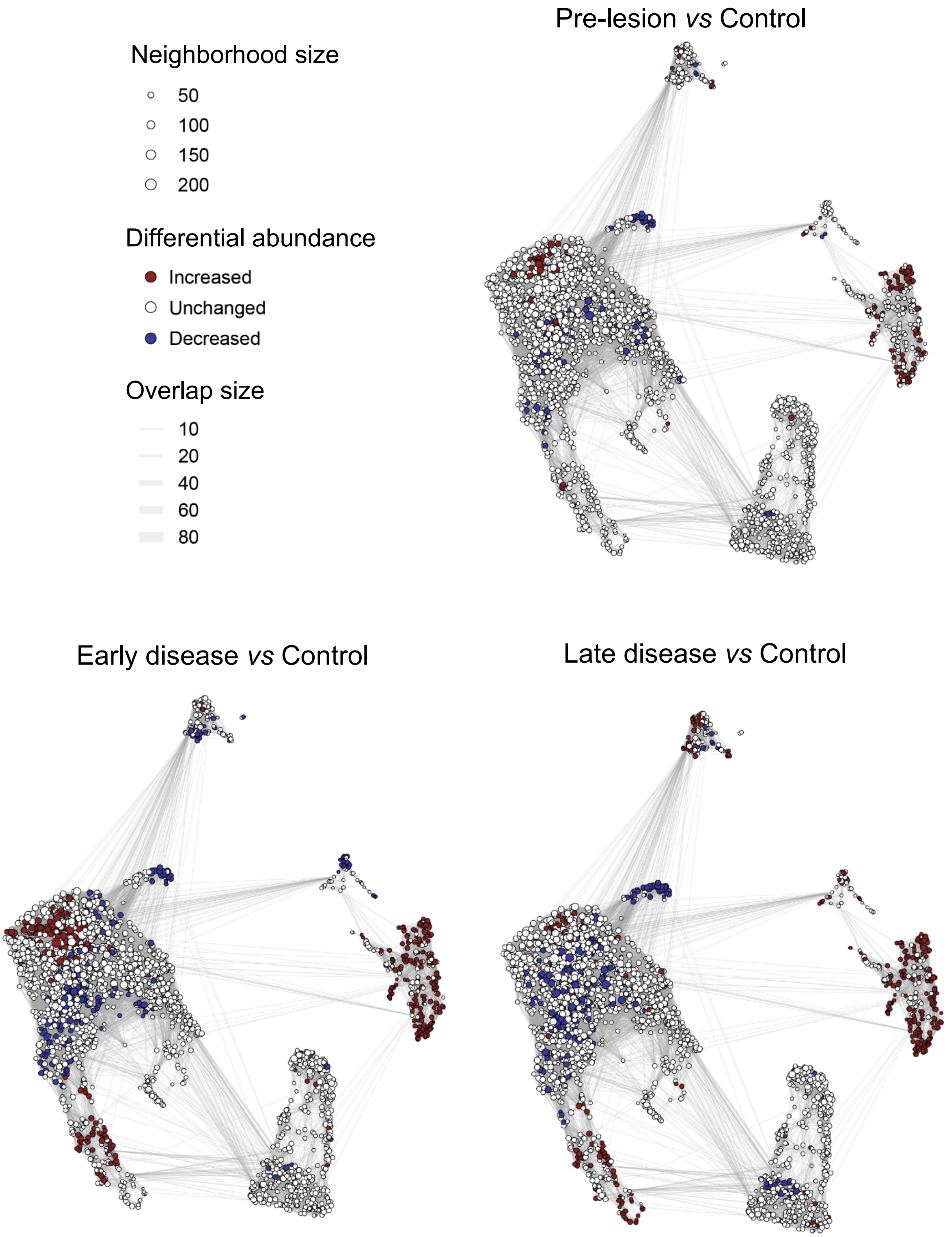

Figure S4. Differentially abundant cellular neighborhoods based on *k*-nearest neighbor graph analysis (Milo[1]). Each stage of disease was compared to control, and significantly increased or decreased neighborhoods (SpatialFDR < 0.1) are indicated by color. The method allows partially overlapping neighborhoods, and the thickness of the lines connecting neighborhoods indicates the number of overlapping cells. The overlap is taken into account when calculating significance in the SpatialFDR procedure.

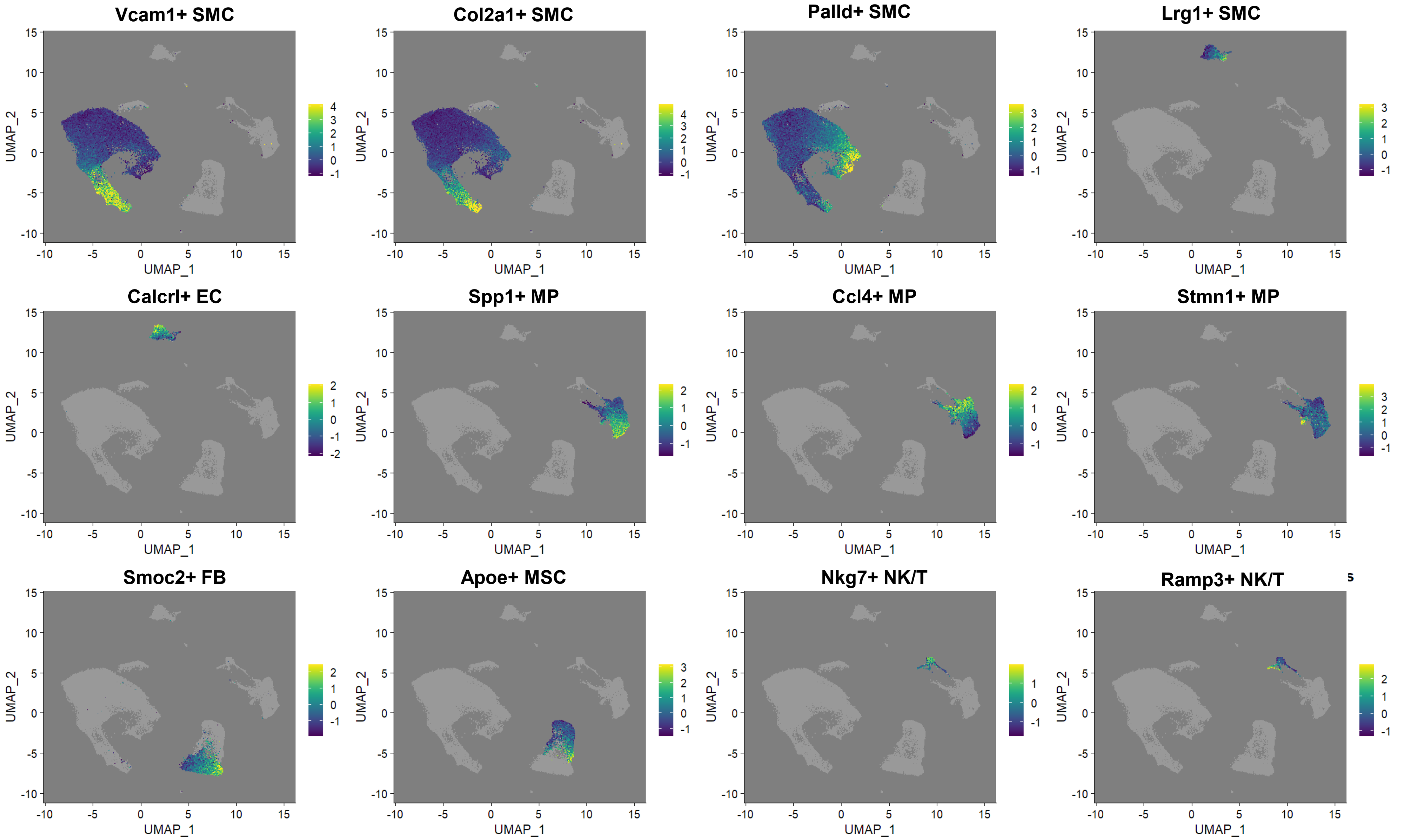

Figure S5. Cell state gene program activity as standard deviation (SD). Cell state marker genes were used as gene sets in the AddModuleScore function of Seurat (expression bin-based averaging). The module scores are presented normalized to the score SD within the cell type.

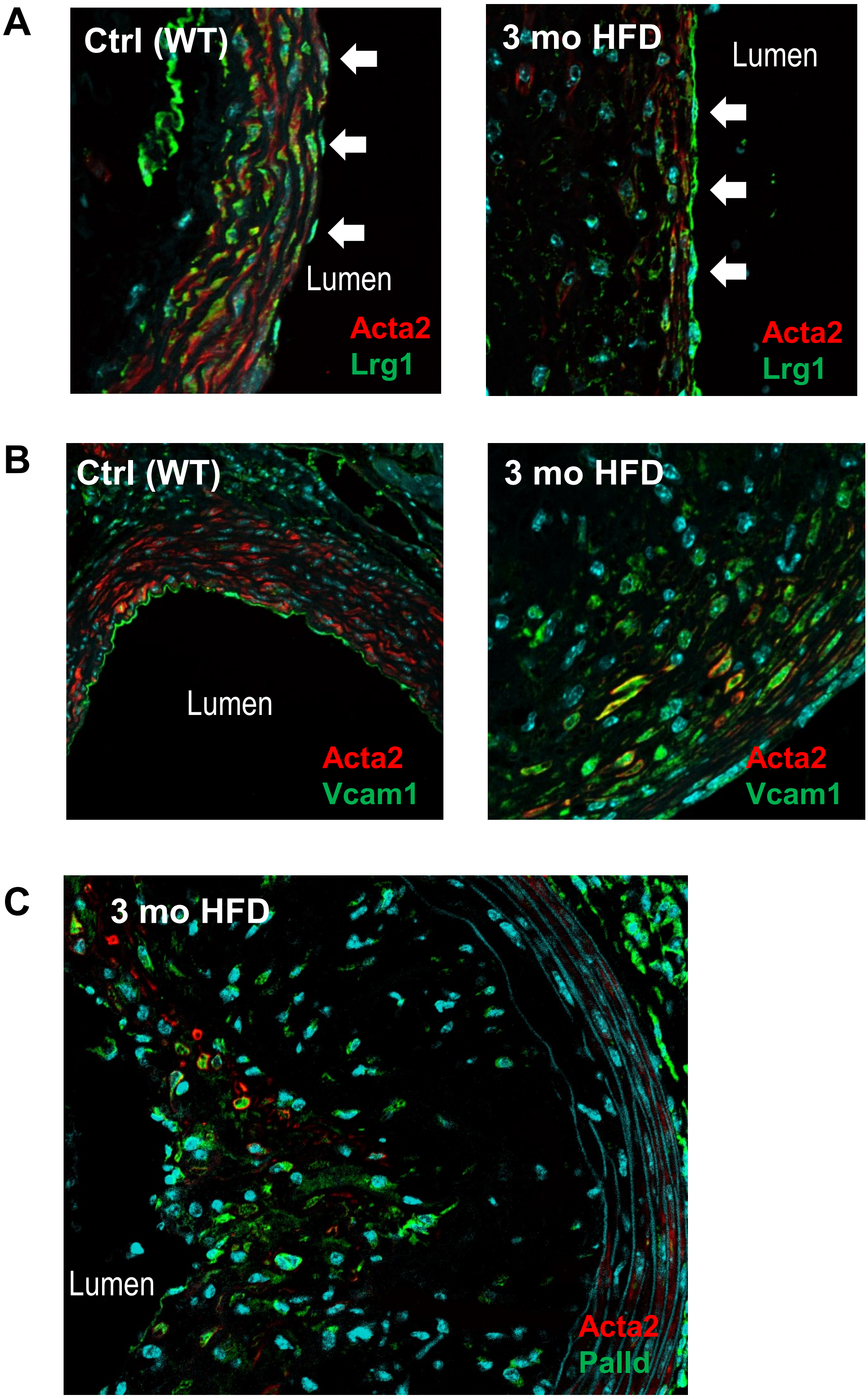

Figure S6. Immunohistological validation of identified cell state marker genes. Representative images of (**A**) Lrg1, (**B**) Vcam1 and (**C**) Palld (all in green) staining in wild type chow diet-fed and *Ldlr*$^{-/-}$/*Apob*$^{100/100}$ 3-month high fat diet mice. The smooth muscle cells are stained with Acta2 antibody (red) and DAPI staining is shown in cyan.

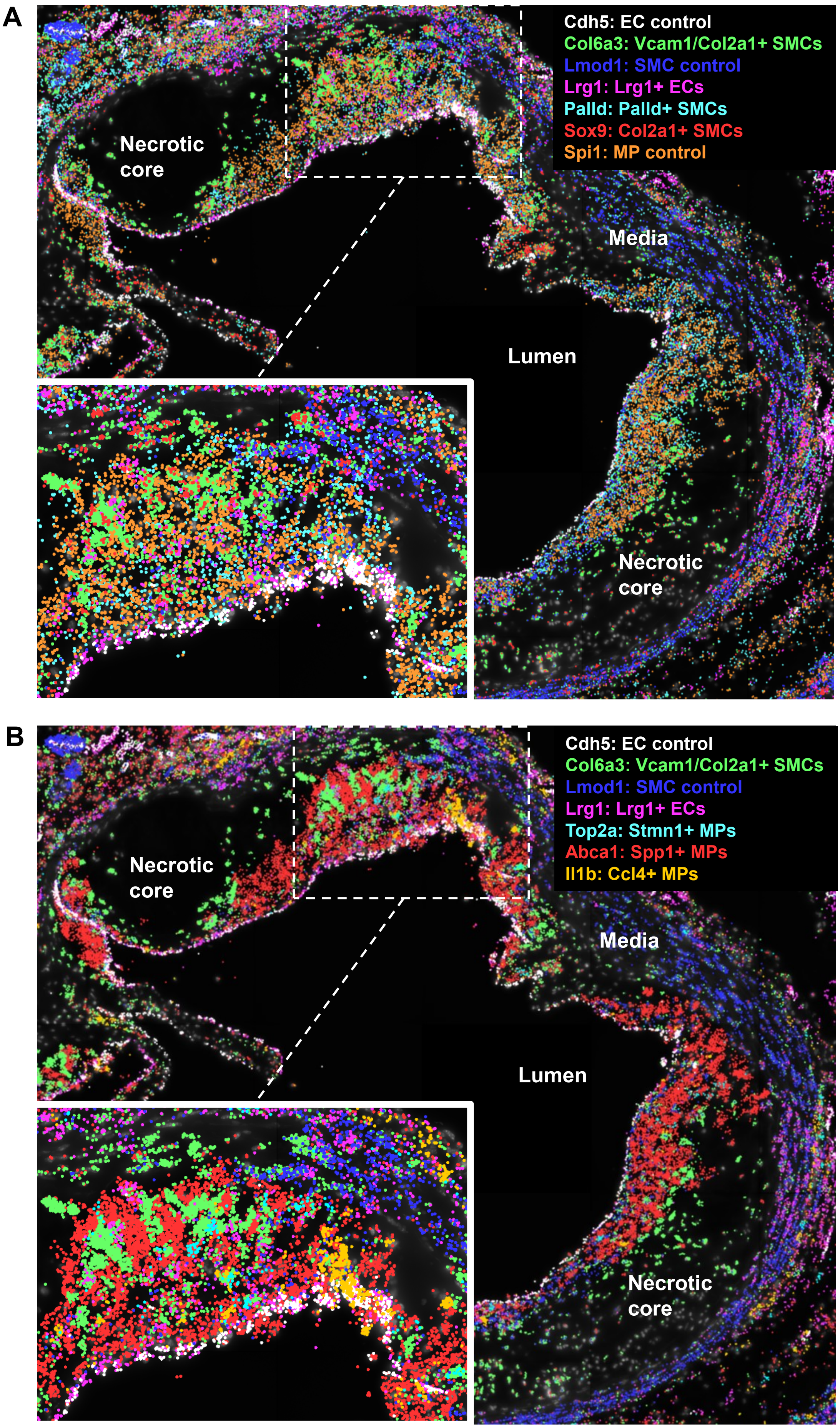
A
Cdh5: EC control
Col6a3: Vcam1/Col2a1+ SMCs
Lmod1: SMC control
Lrg1: Lrg1+ ECs
Palld: Palld+ SMCs
Sox9: Col2a1+ SMCs
Spi1: MP control
Necrotic core
Media
Lumen
Necrotic core
B
Cdh5: EC control
Col6a3: Vcam1/Col2a1+ SMCs
Lmod1: SMC control
Lrg1: Lrg1+ ECs
Top2a: Stmn1+ MPs
Abca1: Spp1+ MPs
Il1b: Ccl4+ MPs
Necrotic core
Media
Lumen
Necrotic core

Figure S7. Molecular Cartography based identification of selected cell states. Distribution of seven selected genes representing the Lrg1+ ECs and (**A**) the three SMC cell states and (**B**) the three MP cell states along with established cell type markers (*Cdh5*, *Lmod1* and *Spi1*) in the aortic root of $Ldlr^{-/-}/Apob^{100/100}$ mice (3-month high fat diet). Each dot represents a single RNA molecule and each pixel equals 138 nm. Insert on the left corner represents magnification of the part of image marked with a dashed line.

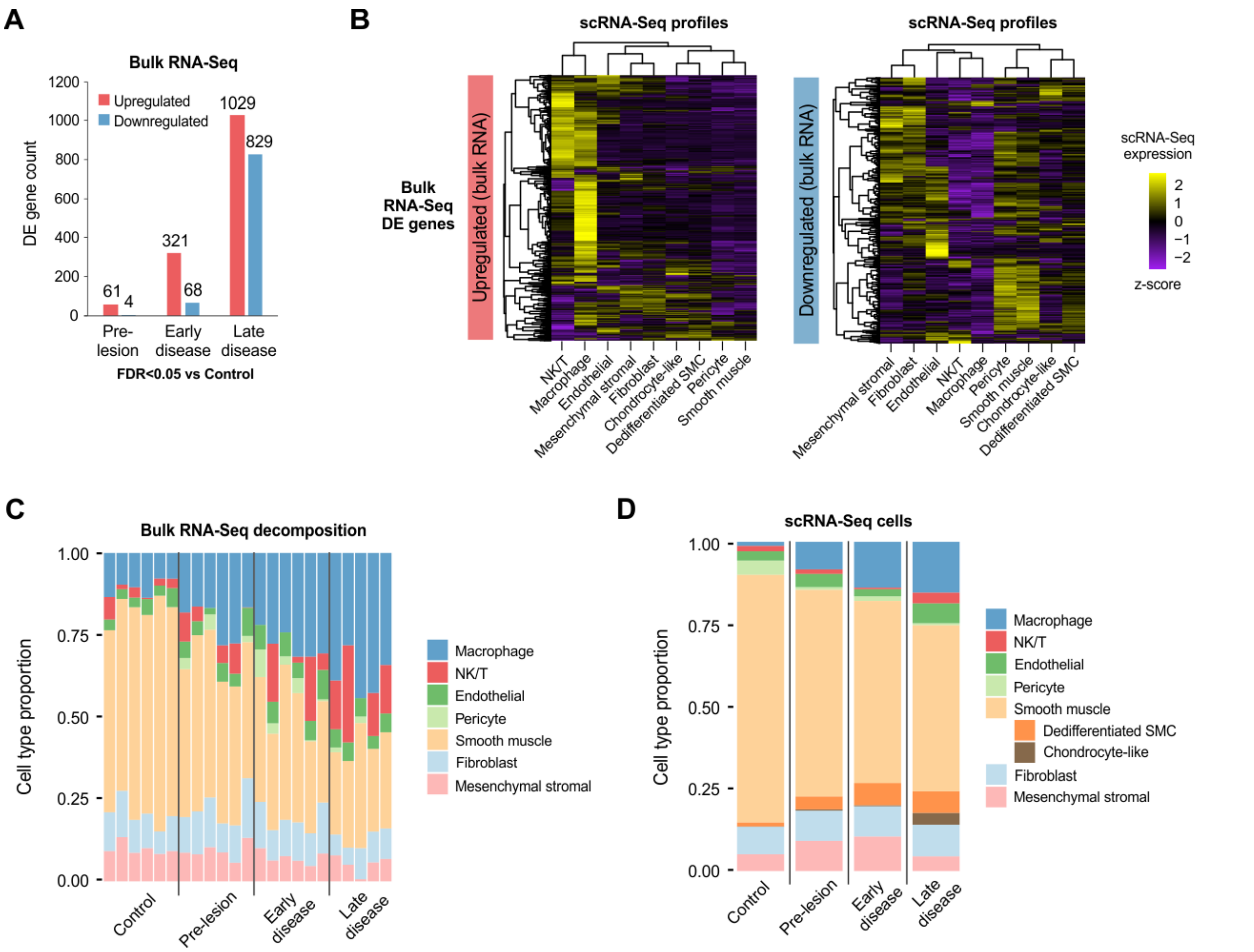

Figure S8. Analysis of cell type proportions from bulk transcriptomics data. (**A**) Number of differentially expressed genes identified from bulk RNA-Seq analysis during different stages of disease progression (see Figure 1A). (**B**) Cell types expressing the genes detected as differentially expressed in bulk RNA-Seq comparison of disease stages. Expression levels of differentially expressed genes from bulk RNA-seq analysis (panel A) are plotted from the cell types identified in scRNA-Seq. (**C**) Computational prediction of cell type composition from the bulk RNA-Seq using the scRNA-Seq data as reference. (**D**) Cell type proportions detected in the scRNA-seq data.

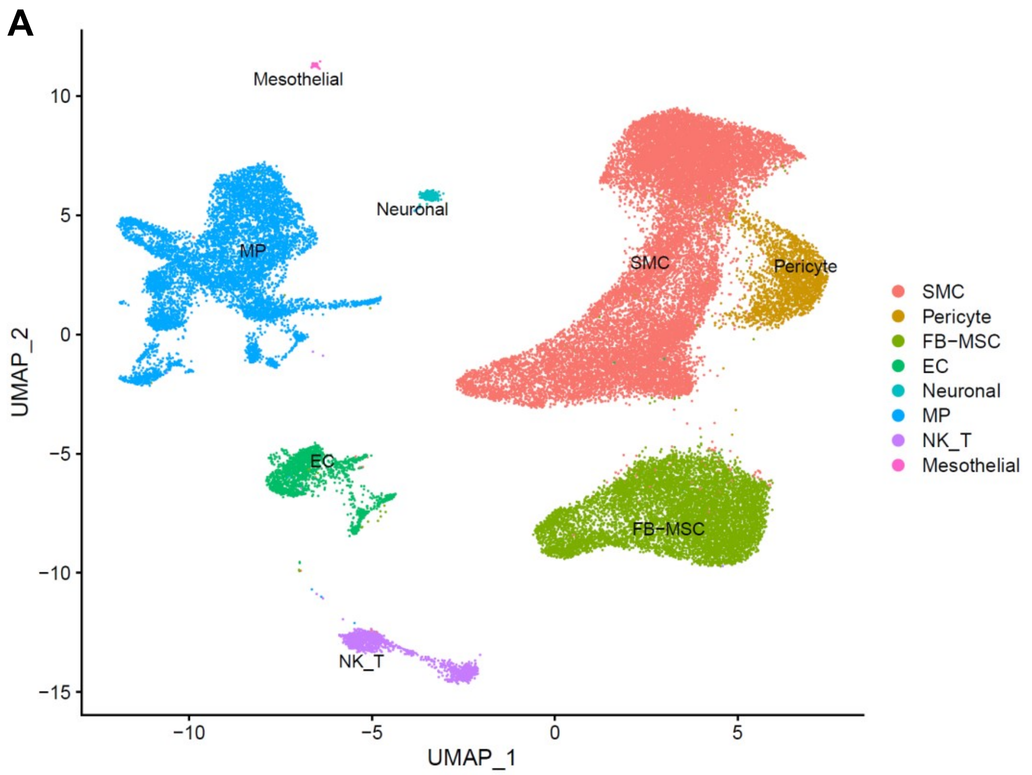

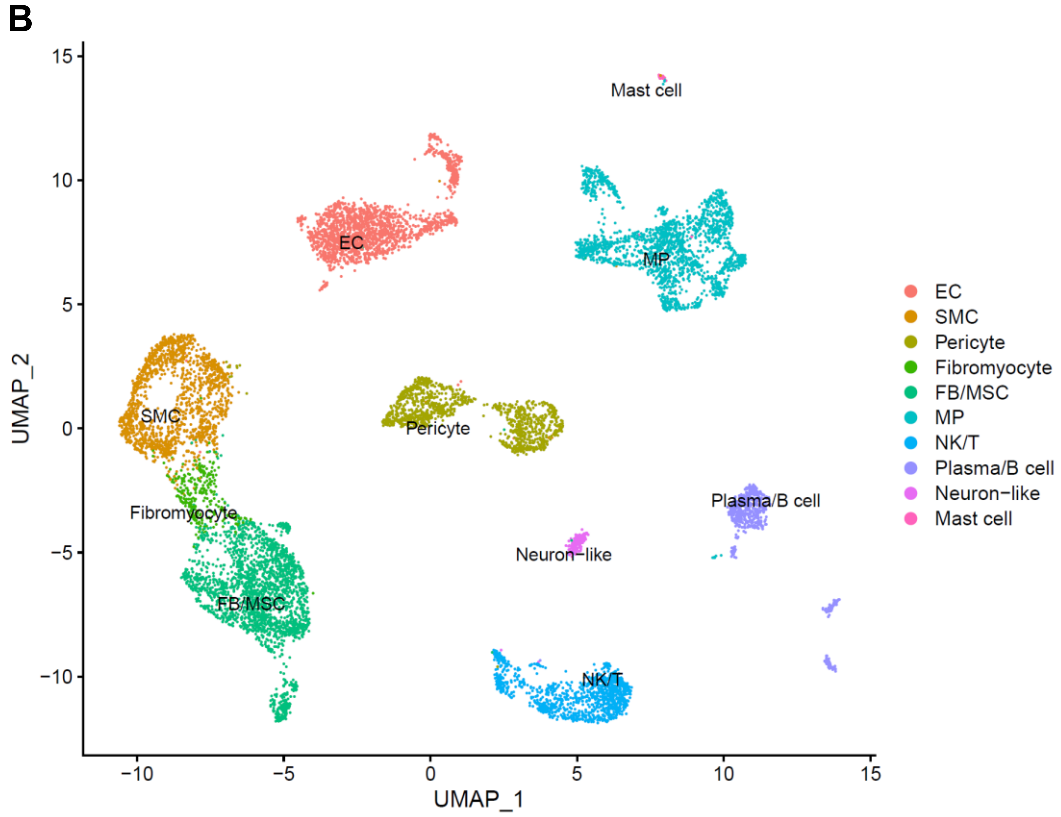

Figure S9. Reprocessing of public scRNA-Seq datasets for cell type identification (A) Reprocessing of mouse scRNA-Seq dataset from Pan *et al*[2] using *Ldlr*$^{-/-}$ and *Apoe*$^{-/-}$ mouse models. **(B)** Reprocessing of scRNA-Seq dataset from four human atherosclerotic coronary arteries[3].

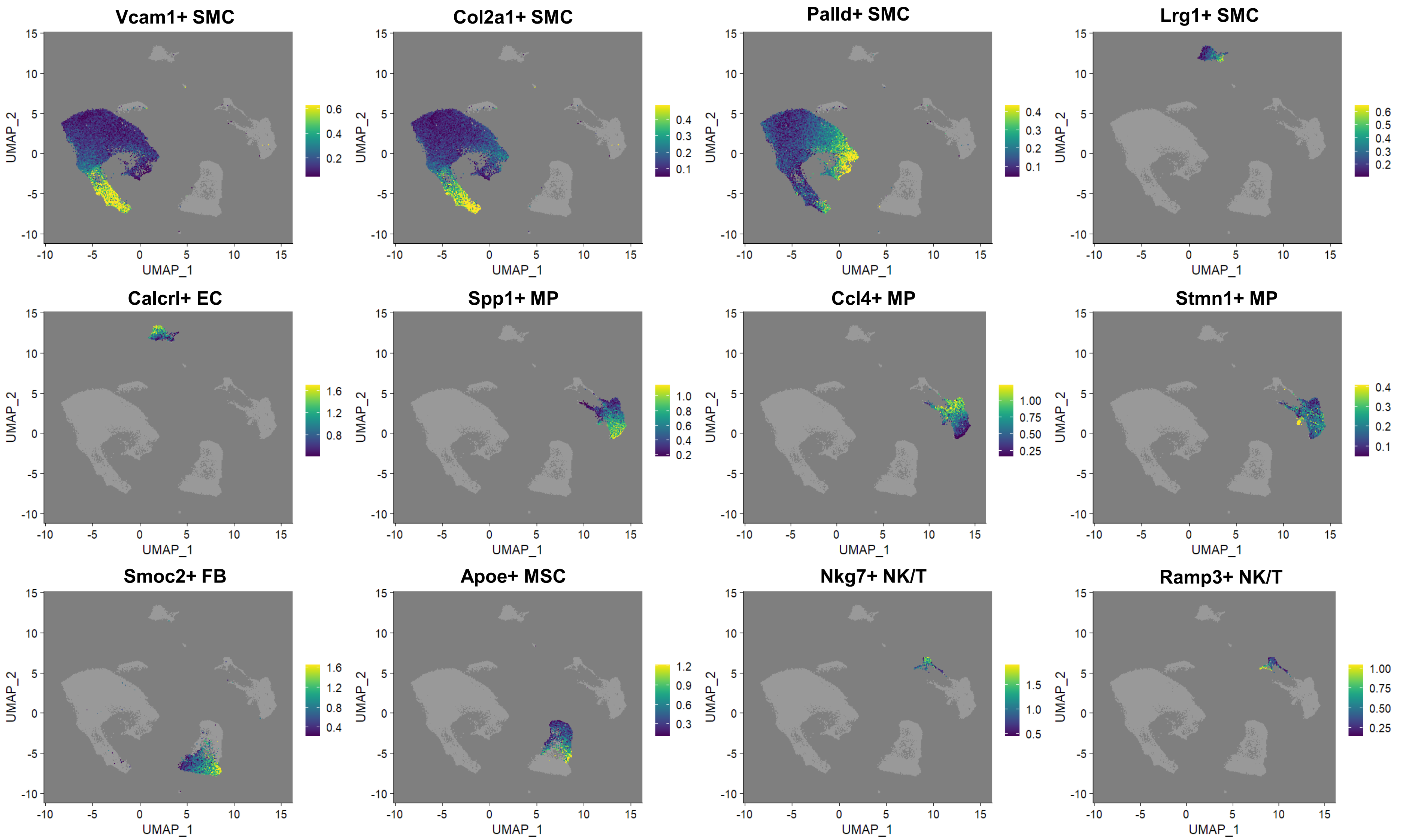

Figure S10. Atherosclerotic cell state marker gene set enrichment scoring. Module scores shown in the target cell type highlights subpopulations of cells with high expression of disease associated markers.

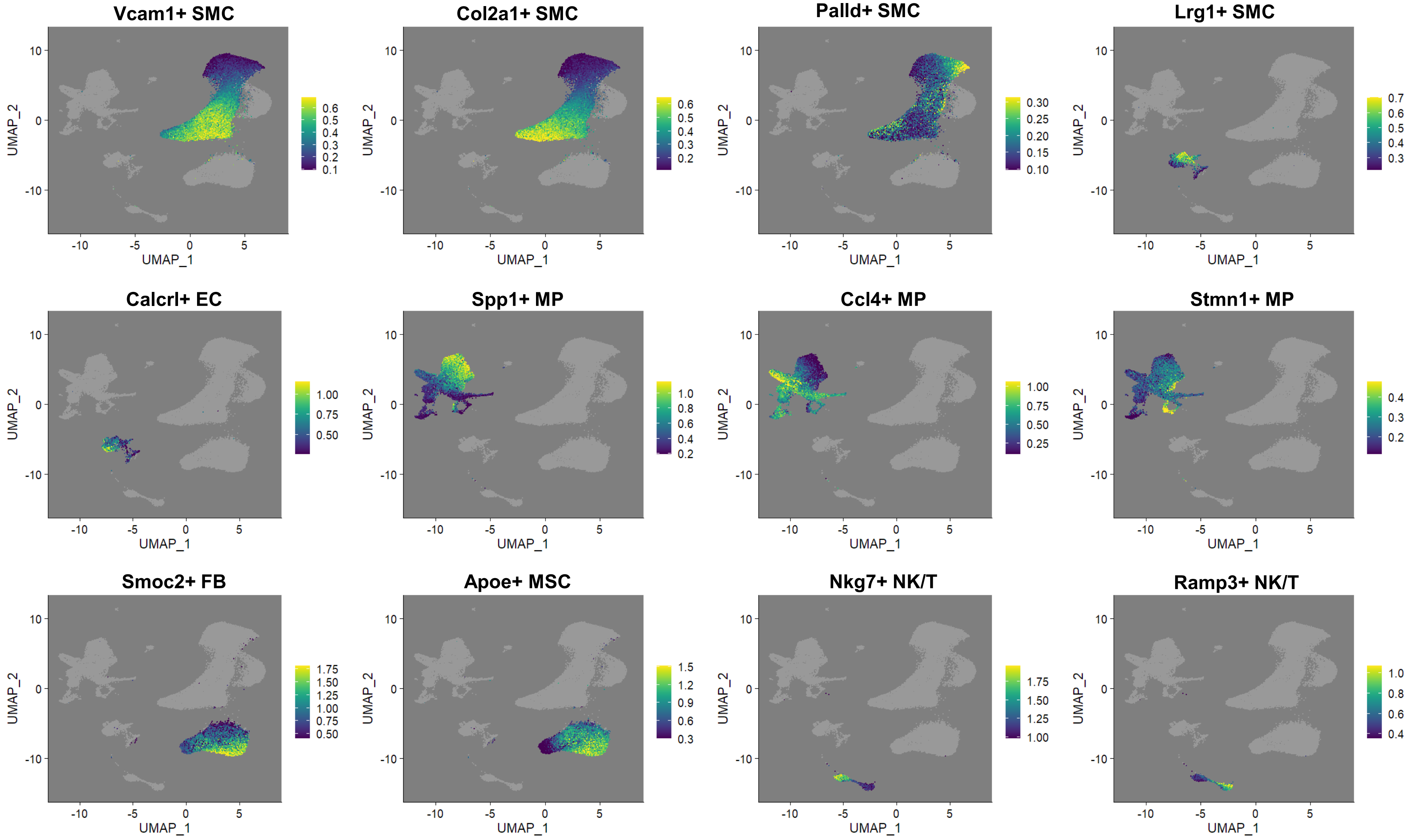

Figure S11. Atherosclerotic cell state marker gene set enrichment scoring in the Pan *et al*[2] *Ldlr*$^{-/-}$ and *Apoe*$^{-/-}$ mouse models scRNA-Seq dataset. Module summary scores (Seurat AddModuleScore; expression bin-based average log fold change) are visualized on a UMAP of the dataset in the cell type of interest (for cluster identifies, see Figure S9A).

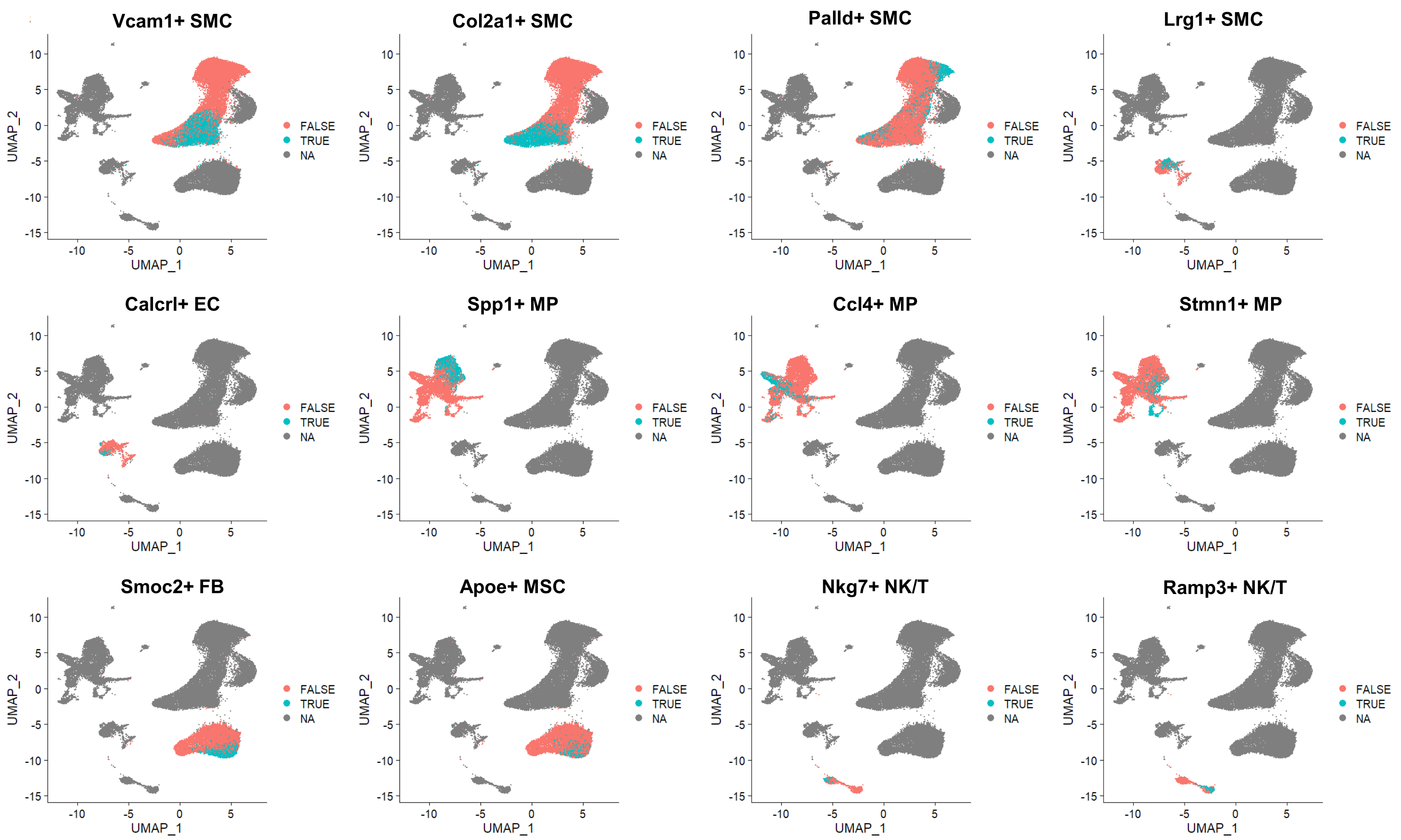

Figure S12. Atherosclerotic cell state marker gene set enrichment scoring in the Pan *et al*[2] dataset. Cells were classified as either positive or negative for the gene program using a cutoff of >1 standard deviation. This binary classification of the cells used to generate Figure S13.

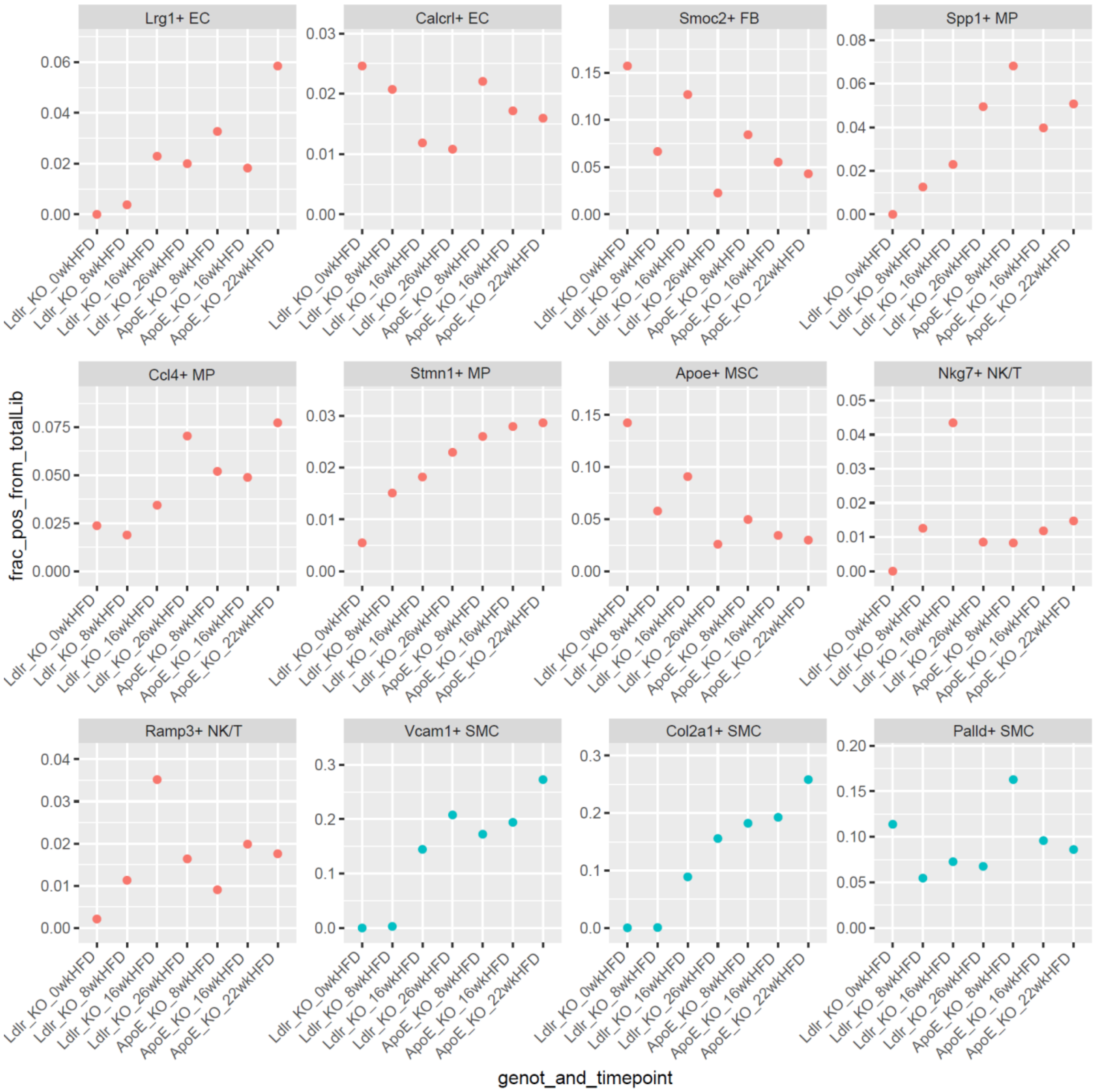

Figure S13. Relative changes in the cell state proportions in different models and timepoints of atherosclerosis in the Pan *et al*[2] dataset. Fraction of cell state-positive cells (as defined in Figure S12) from the total library are presented. SMC-related cell states are shown from *Myh11* (*ZsGreen*) lineage-positive libraries (blue dots), while all others are shown from *Myh11* (*ZsGreen*) lineage-negative libraries (red dots).

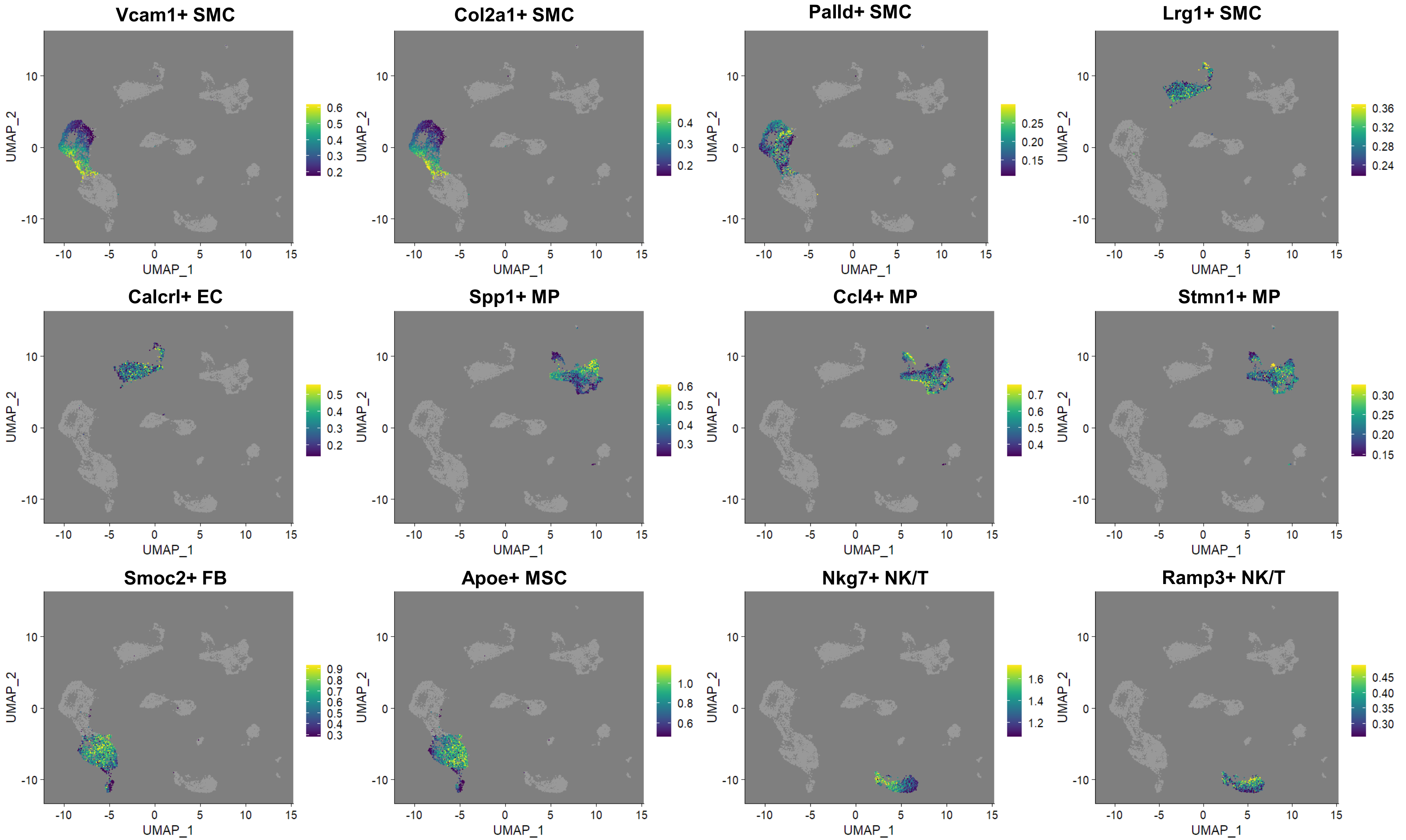

Figure S14. Atherosclerotic cell state marker gene set enrichment scoring in the Wirka *et al*[3] human coronary artery scRNA-Seq dataset. Module summary scores (Seurat AddModuleScore; expression bin-based average log fold change) are visualized on a UMAP in the target cell cluster (for cluster identities, see Figure S9B).

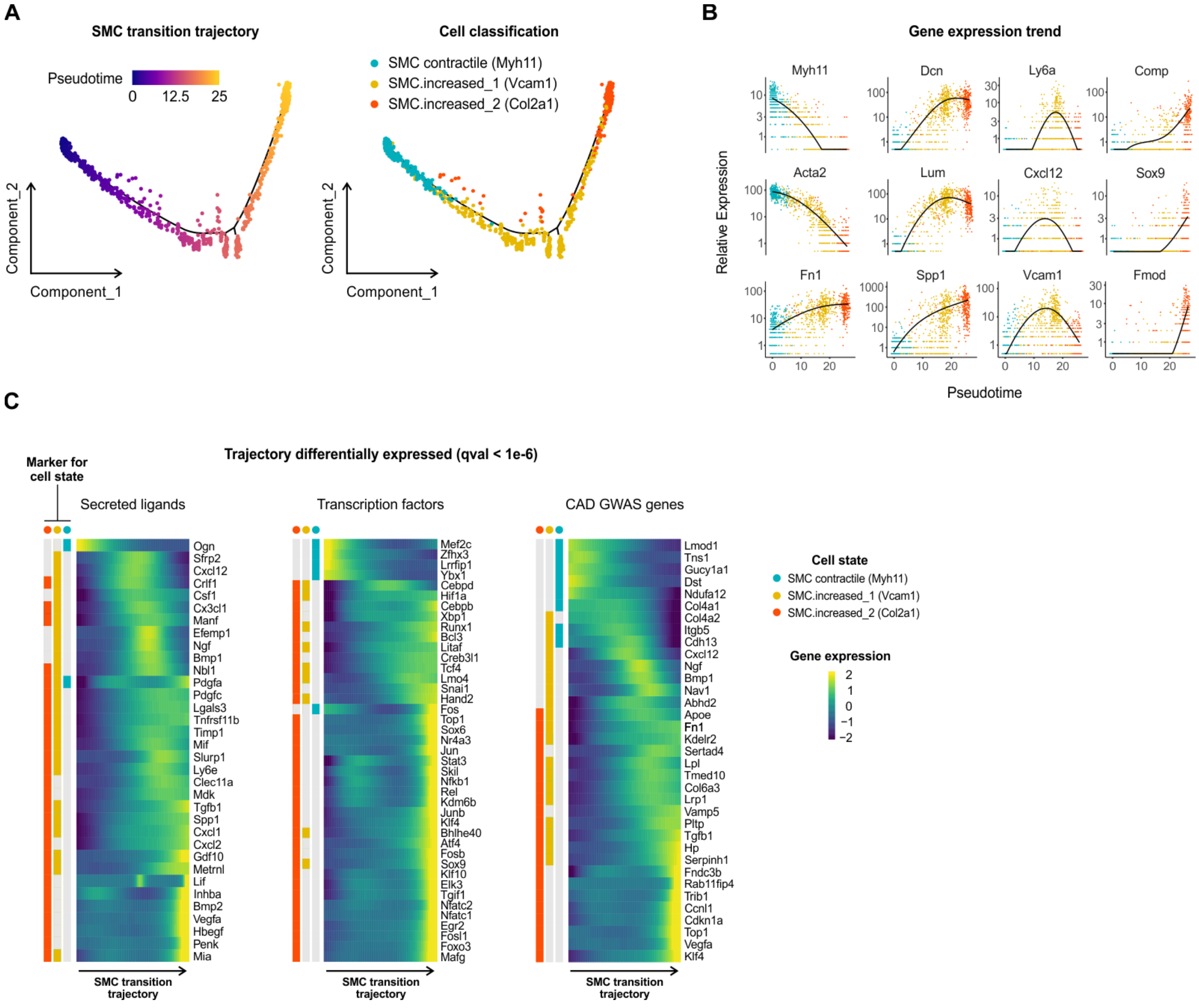

Figure S15. Pseudotime analysis of SMC cell states from atherosclerotic aorta. (**A**) scRNA-Seq trajectory analysis of SMC states colored by pseudotime and cell state assignment. (**B**) Gene expression changes of selected marker genes along the pseudotime trajectory. Cells are colored by cell state as in panel A. (**C**) Expression changes of secreted ligands, transcription factors and CAD GWAS genes that display differential expression along the pseudotime trajectory.

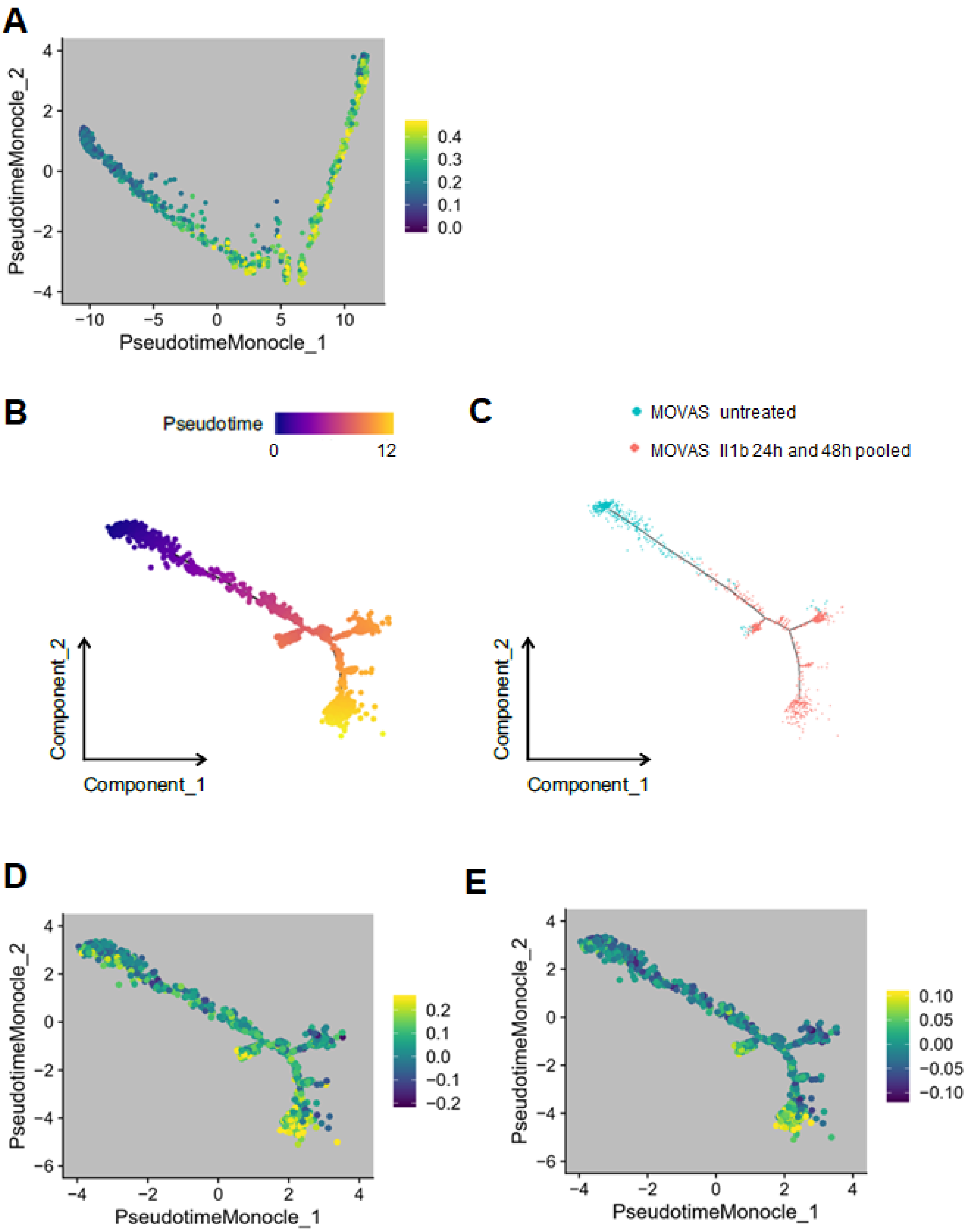

Figure S16. Projection of IL-1β responsive genes on *in vivo* SMC disease response pseudotime trajectory. (A) Differentially expressed genes identified using scRNA-seq of *in vitro* SMCs (MOVAS cells) upon 24 and 48 h IL-1β treatment plotted on the *in vivo* SMC trajectory (see Figure S15A) as a gene set activity score. scRNA-Seq trajectory analysis of *in vitro* SMCs under basal and IL-1β treatment conditions colored by (**B**) pseudotime and (**C**) treatment. (**D**) Vcam1+ SMC and (**E**) Col2a1+ SMC marker gene sets plotted as activity scores on the *in vitro* SMC IL-1β response trajectory (described in panels B-C).

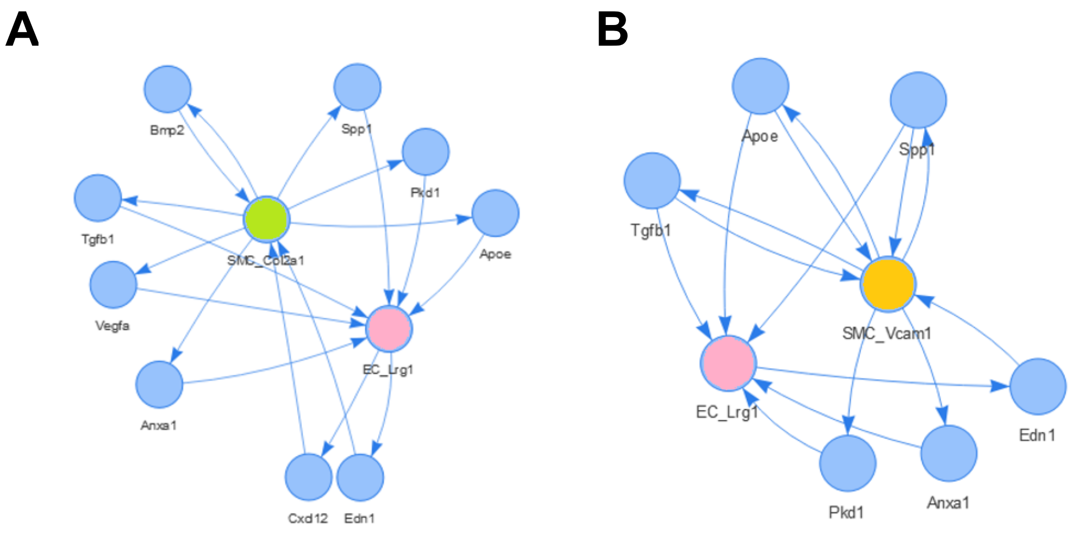

Figure S17. Prediction of cell-cell signaling networks. Ligands predicted to mediate paracrine and autocrine signaling between Lrg1+ ECs and either (**A**) Col2a1+ SMCs or (**B**) Vcam1+ SMCs.

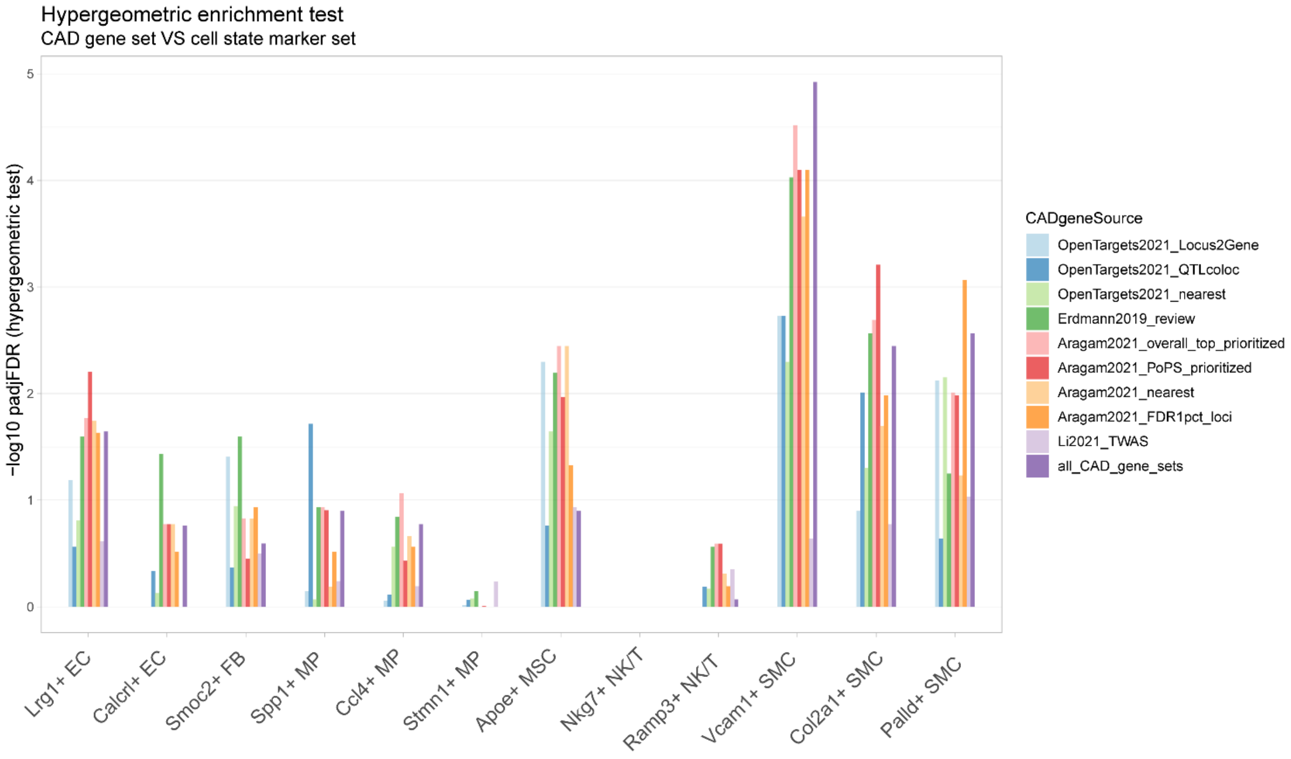

Figure S18. Enrichment of CAD GWAS genes within the disease associated cell states gene signatures. Hypergeometric enrichment test results are shown for CAD GWAS candidate causal gene lists from 9 different sources separately or all combined (different colors). The set of background genes for enrichment testing was all genes expressed at >1 TPM in at least one cell type or cell state in aorta scRNA-Seq (total 14902 genes).

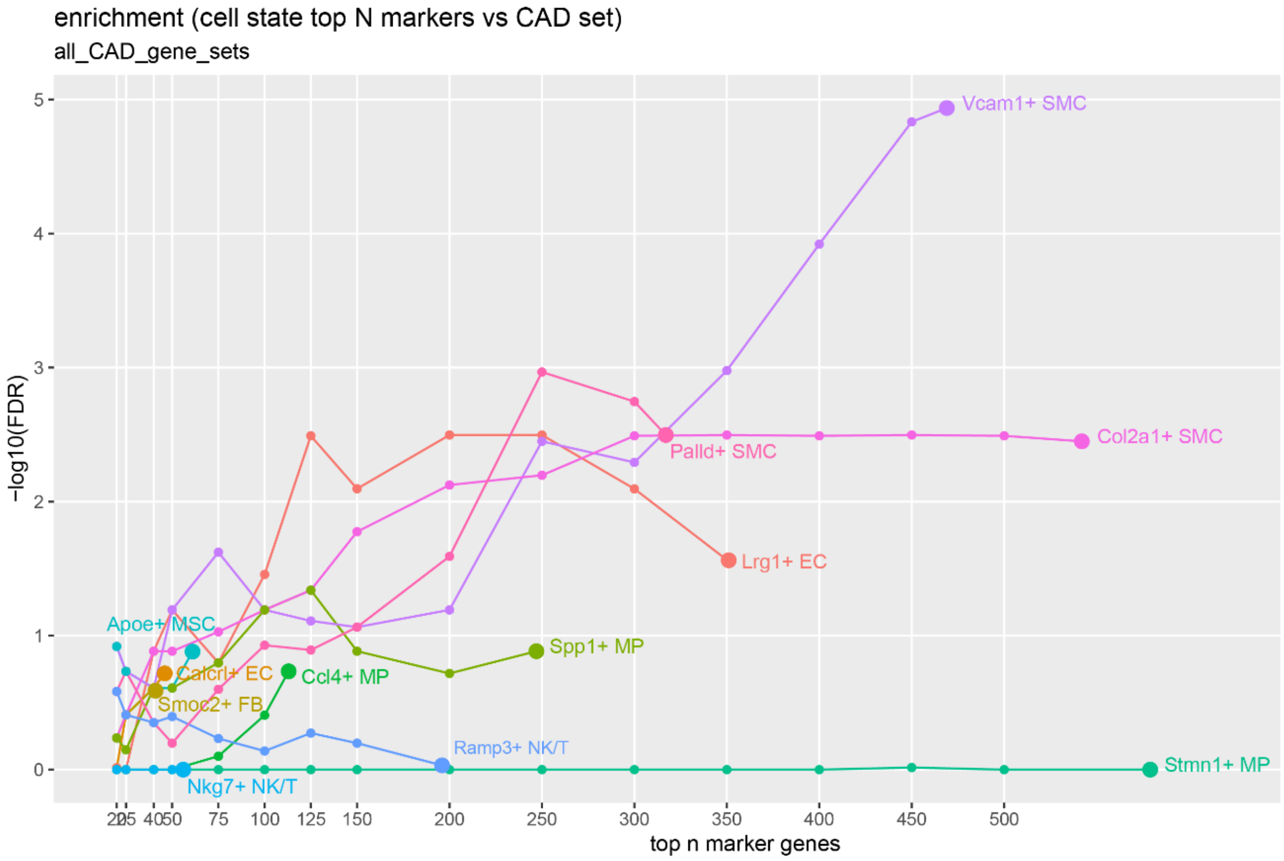

Figure S19. Enrichment of CAD GWAS prioritized genes in cell state marker sets truncated to a specific gene count. The indicated number of top markers were selected for each cell state (up to the total number of marker genes available). The set of CAD GWAS genes was prioritized genes from all 9 sources combined (Methods). The set of background genes for enrichment testing was all genes expressed at >1 TPM in at least one cell type or cell state in aorta scRNA-Seq (total 14902 genes).

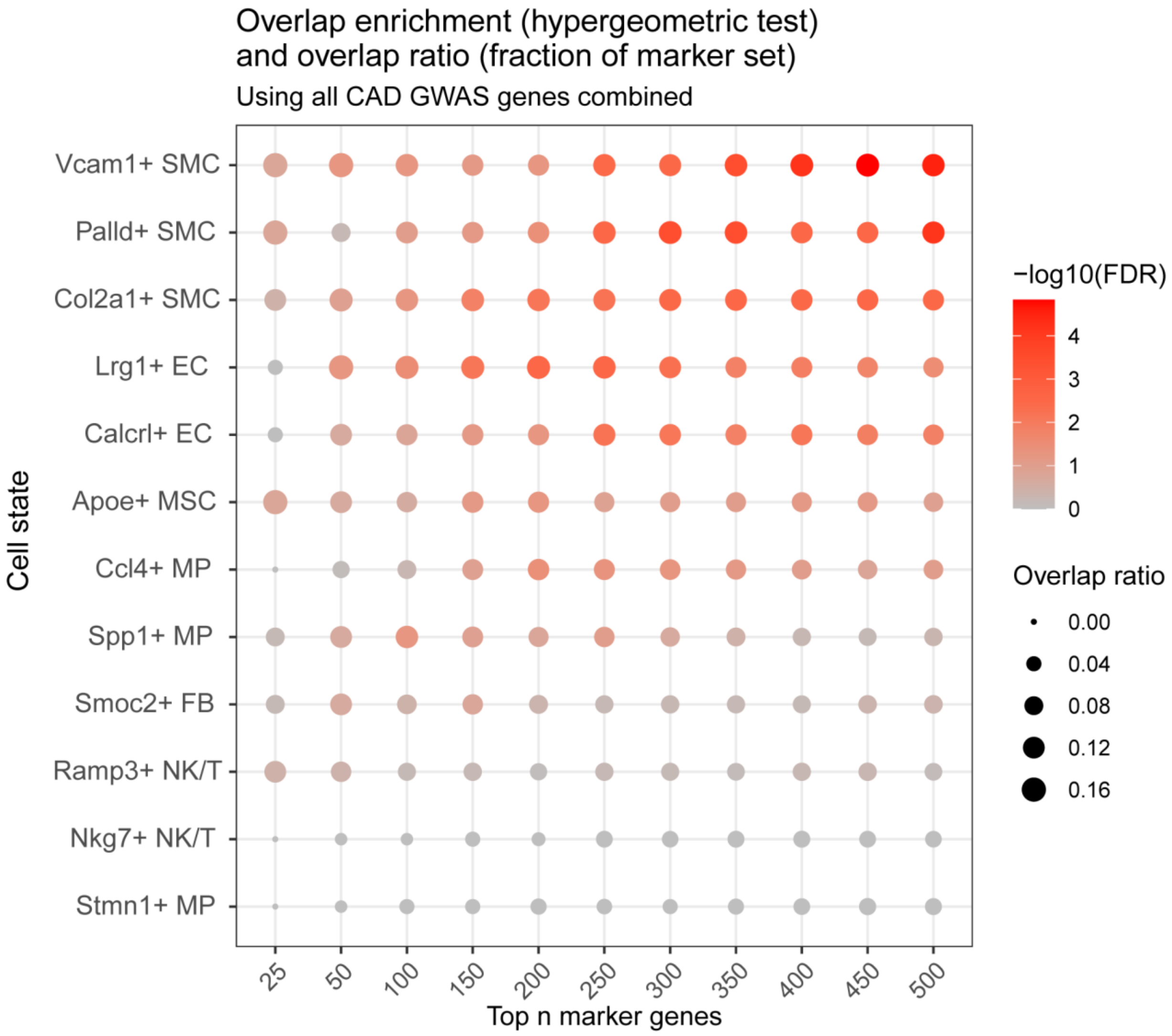

Figure S20. Enrichment of CAD GWAS prioritized genes in cell state marker lists equalized for gene count by including sub-threshold marker genes. The indicated number of top markers were selected for each cell state using log fold change ranking. The required number of top genes were selected irrespective of whether the marker gene criteria (log fold change > 0.25 and FDR < 0.05; Methods) were fulfilled. Overlap enrichment was tested by hypergeometric test. The set of CAD GWAS genes was prioritized genes from all 9 sources combined (see Methods). The set of background genes for enrichment testing was all genes expressed at >1 TPM in at least one cell type or cell state in aorta scRNA-Seq (total 14902 genes). Column and row order is by average -log10(FDR). The dot size indicates the ratio of CAD GWAS genes within the marker gene set.

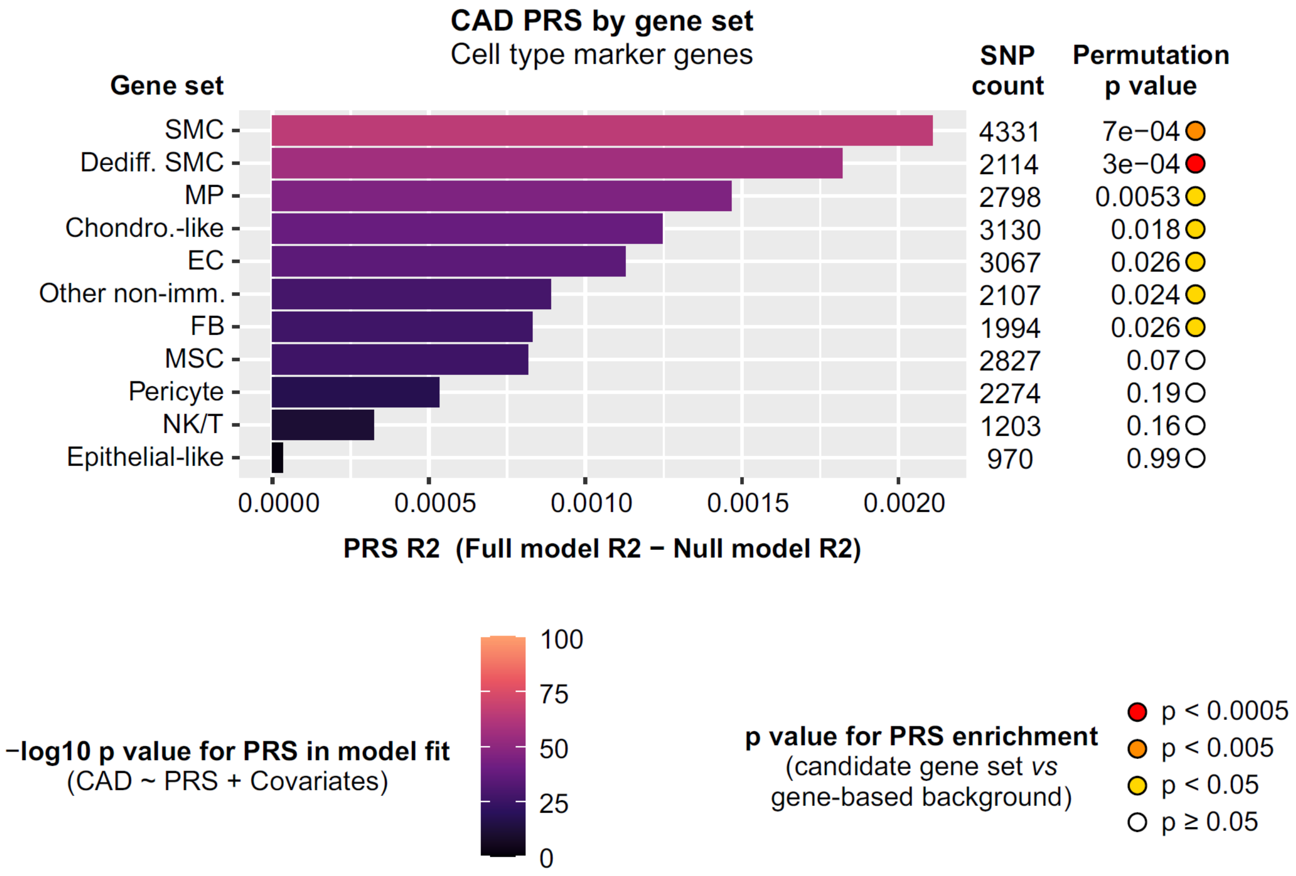

Figure S21. Proportion of variance of CAD explained by the cell type specific marker genes. PRS was constructed using the cell type specific marker gene coordinates (-35 kb upstream to 10 kb downstream; gene list in Table S1) using PRSet[4].

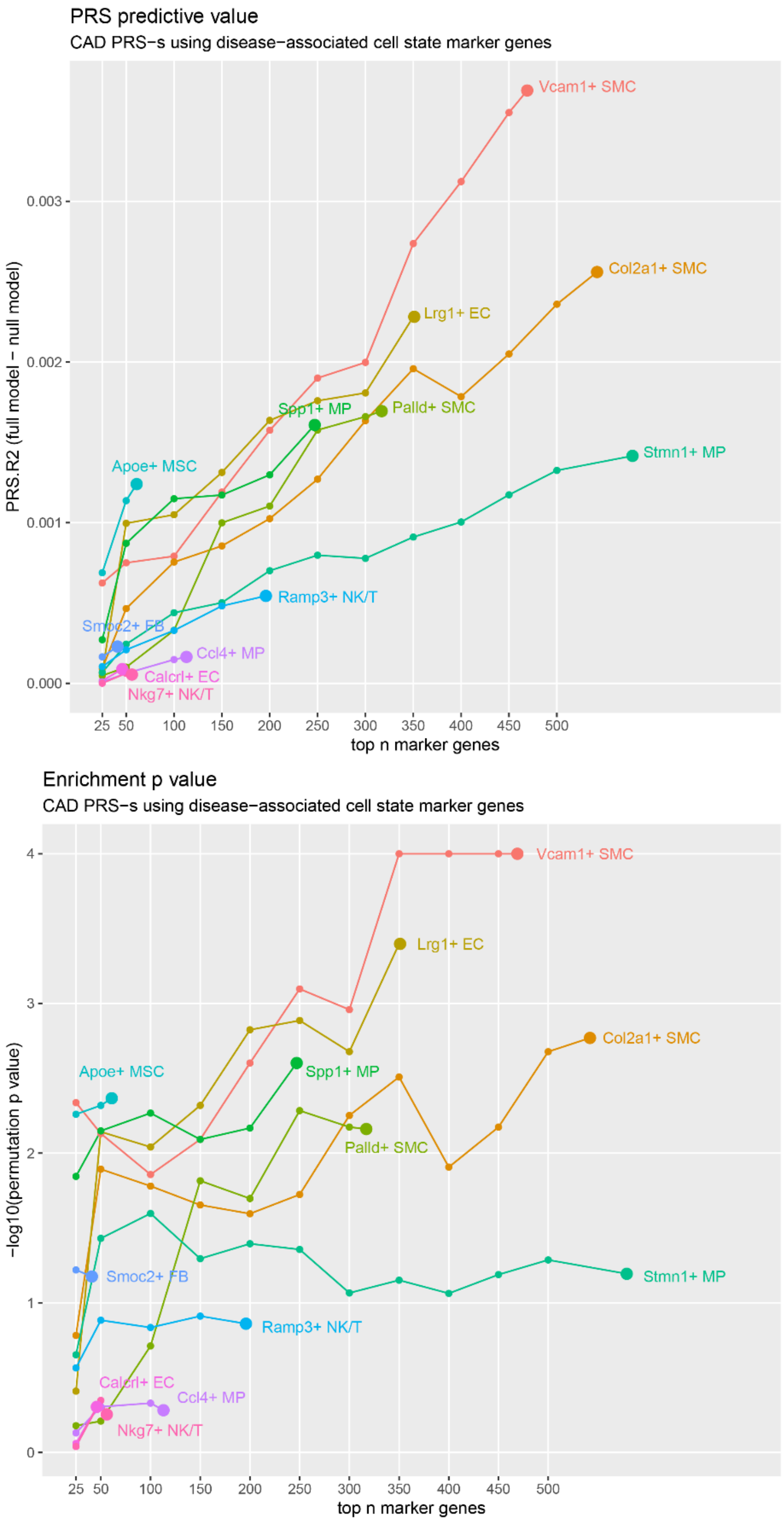

Figure S22. Evaluation of gene set-based PRS for CAD as a function of the number of cell state marker genes selected. The indicated number of top marker genes were retained (up to the maximum available). Upper panel: PRS predictive power (PRS.R2 = full model R2 – null model R2; the difference in R2 for a model including PRS and covariates, compared to a model with only covariates). Bottom panel: permutation-based significance test comparing the performance to identically-clumped SNP sets from background regions (genes + flanks), as implemented by PRSet[4].

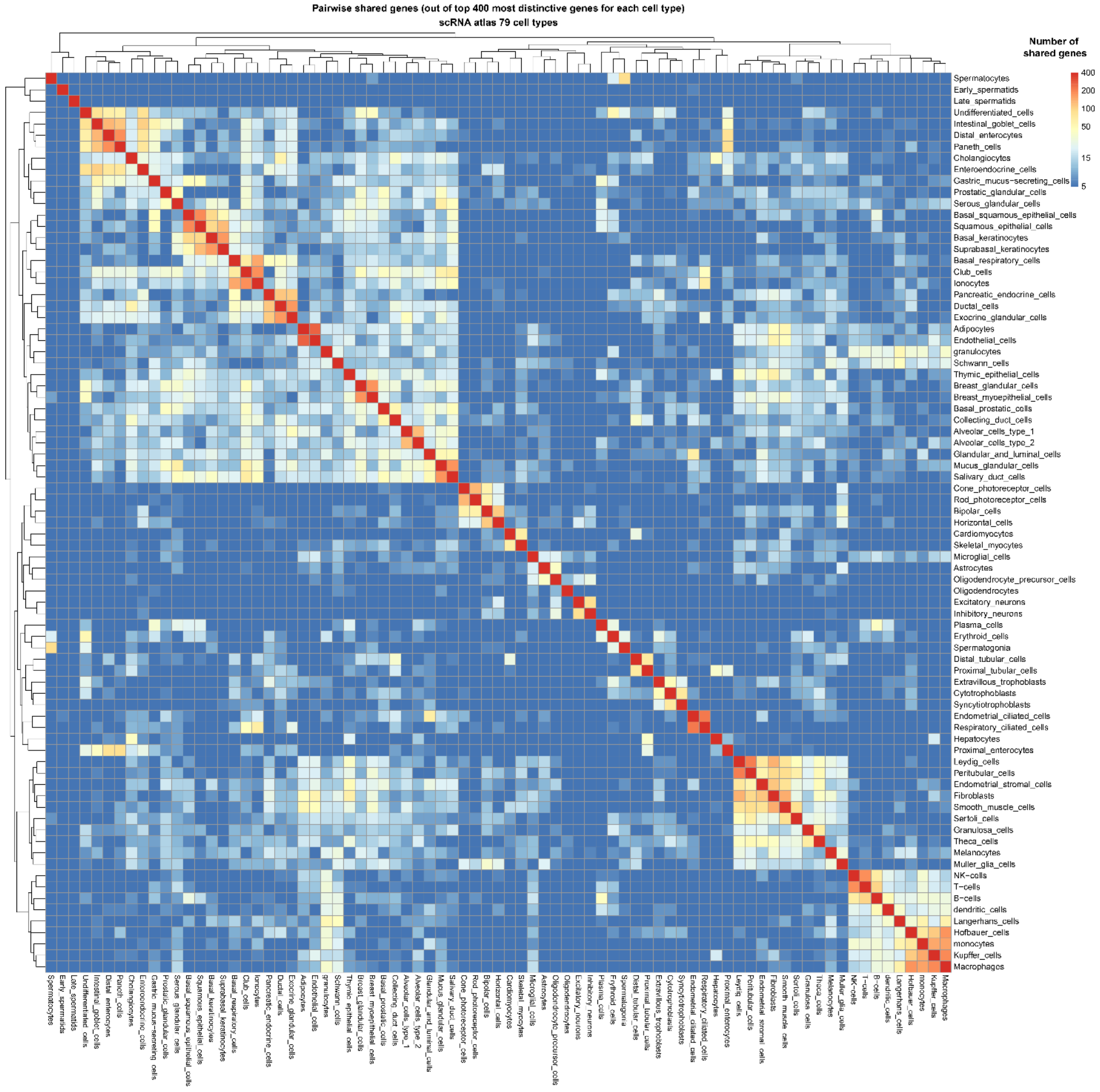

Figure S23. Number of pairwise shared marker genes for 79 human cell types. Marker genes were selected as described in Methods using gene expression profiles compiled by the Protein Atlas from 30 scRNA-Seq datasets. For each cell type, the top 400 marker genes were used. Color scale shows the number of shared genes.

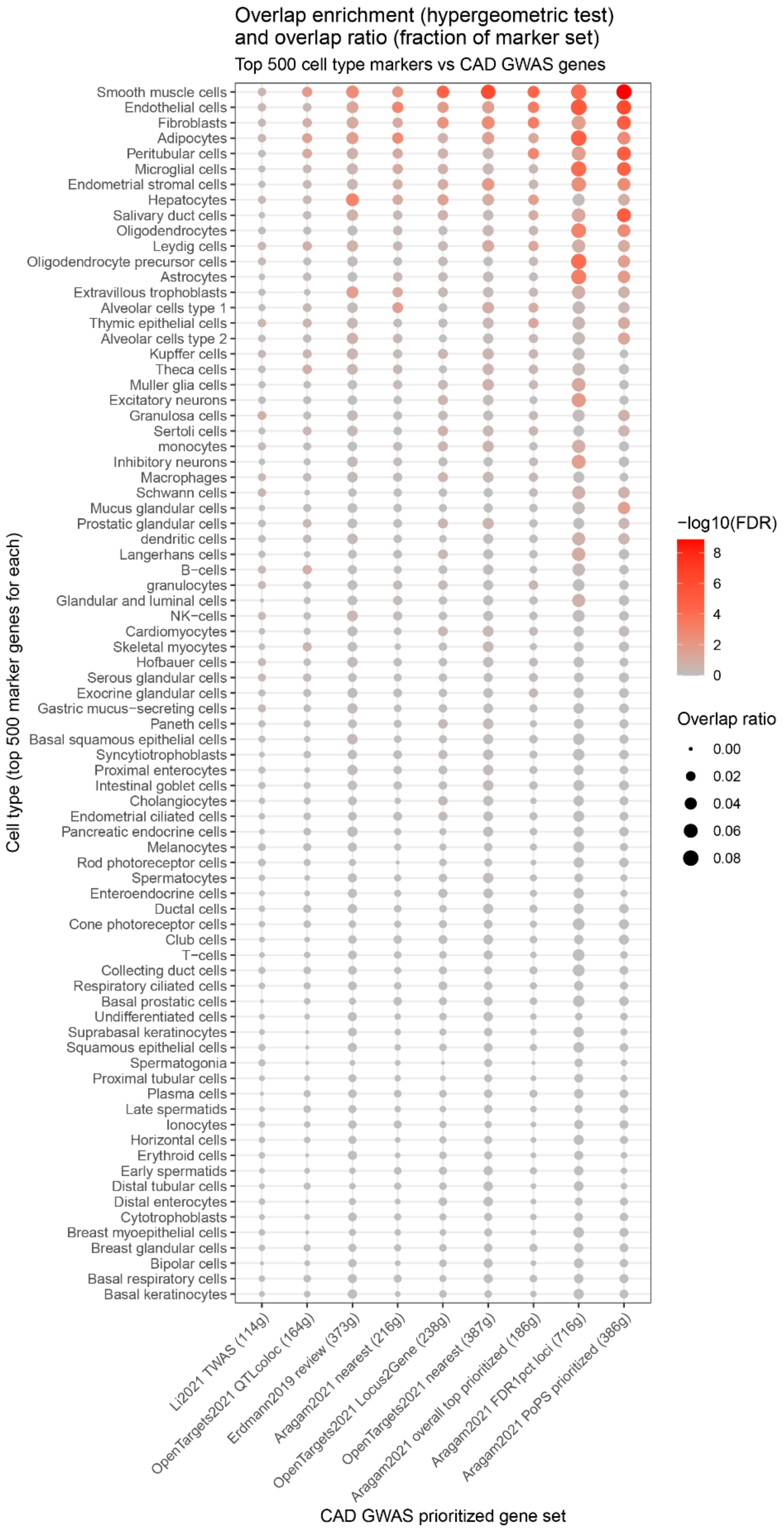

Figure S24. Enrichment of CAD GWAS prioritized genes among the top 500 cell type markers of 79 human cell types. Markers were selected as described in Methods. Overlap enrichment was tested by hypergeometric test using 18043 genes as the background. Column and row order is by average -log10(FDR). The dot size indicates the ratio of CAD GWAS genes within the marker gene set.

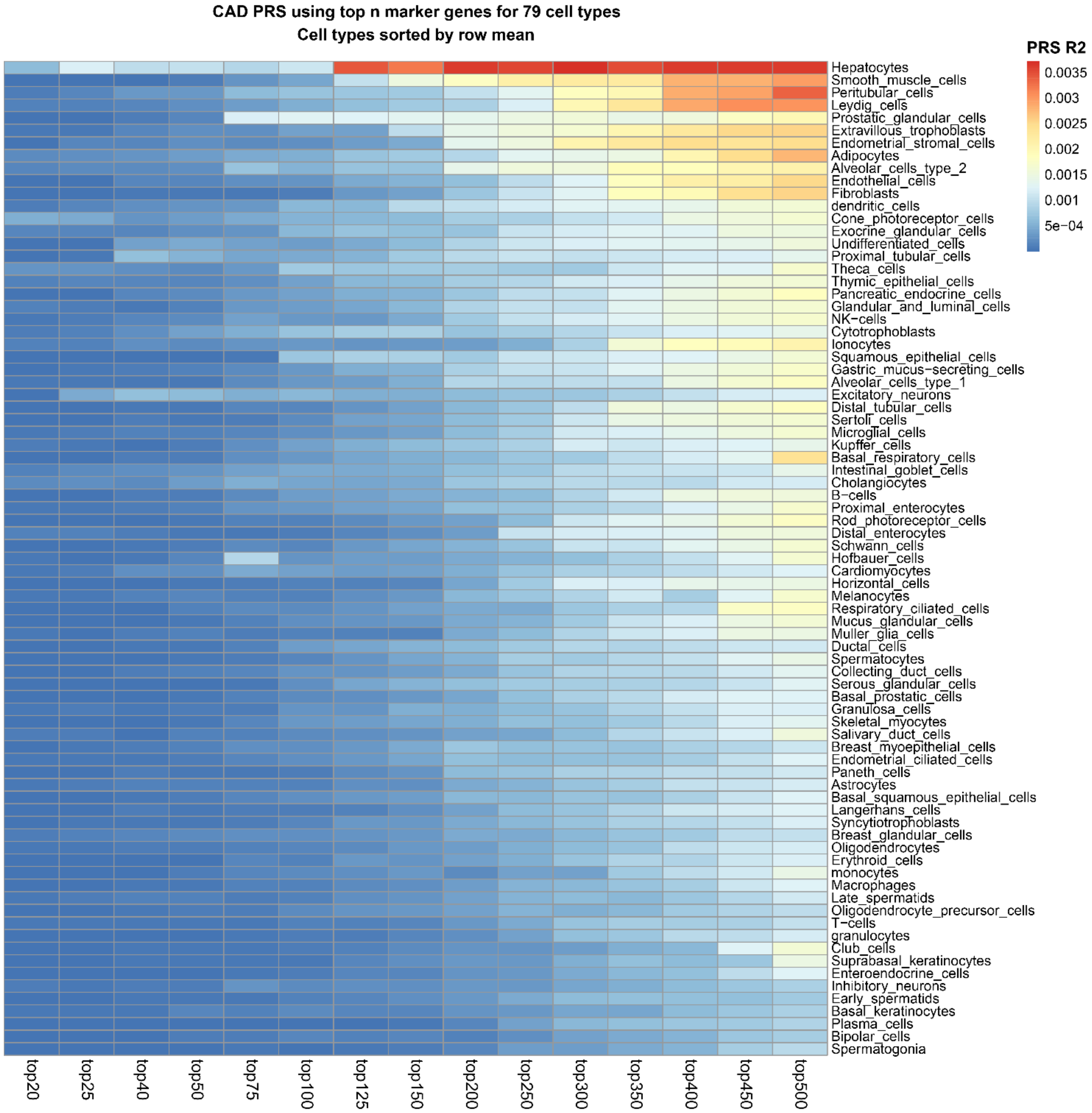

Figure S25. Gene set-based PRS for CAD using marker gene sets for 79 human cell types. The indicated number of top marker genes was selected for each cell type and used to define a PRS using the gene bodies + flanks. Color scale shows the PRS.R2 (full model R2 – null model R2, i.e., the difference in R2 for a model with PRS and covariates, compared to a model with only covariates). Cell types are sorted according to row mean.

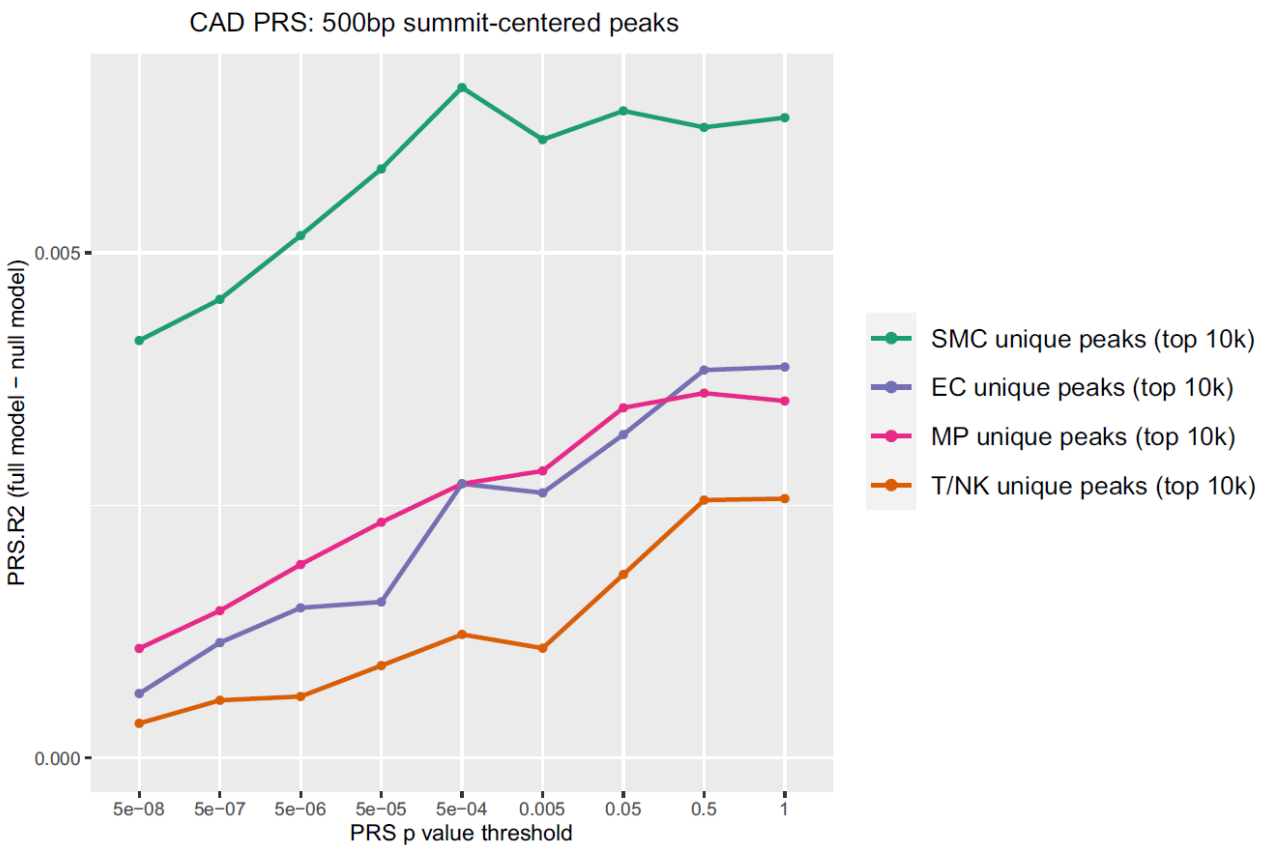

Figure S26. Proportion of variance of CAD explained by polygenic risk score (PRS) calculated using to 10,000 strongest cell type-specific peaks of each cell type. Peaks were ranked by ATAC signal score as calculated by the peak caller (MACS2)[5]. Peaks were each 500 bp in length. Thus, the fraction of genome included in the search space based for each cell type is equal-sized and non-overlapping between the cell types.

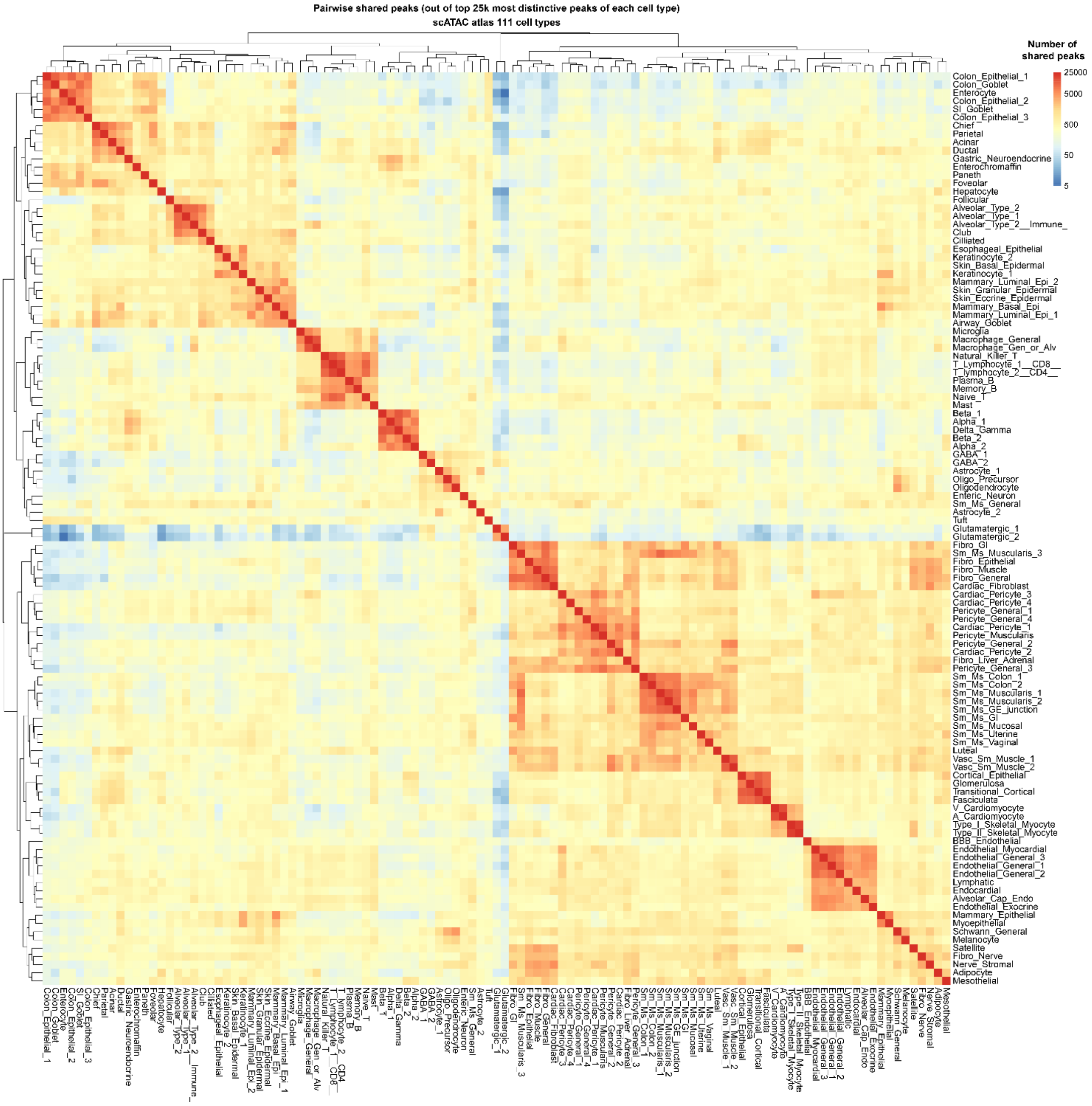

Figure S27. Number of pairwise shared scATAC-Seq peaks among top 25,000 most distinctive peaks of 111 human cell types. All adult cell types of the human scATAC-Seq atlas[6] were used. All peaks were of equal width. The cell type peak accessibility matrix was z-scored per peak, and the top 25,000 peaks were selected per cell type to obtain the most distinctive peaks. Color scale shows the number of shared peaks between pairs of cell types.

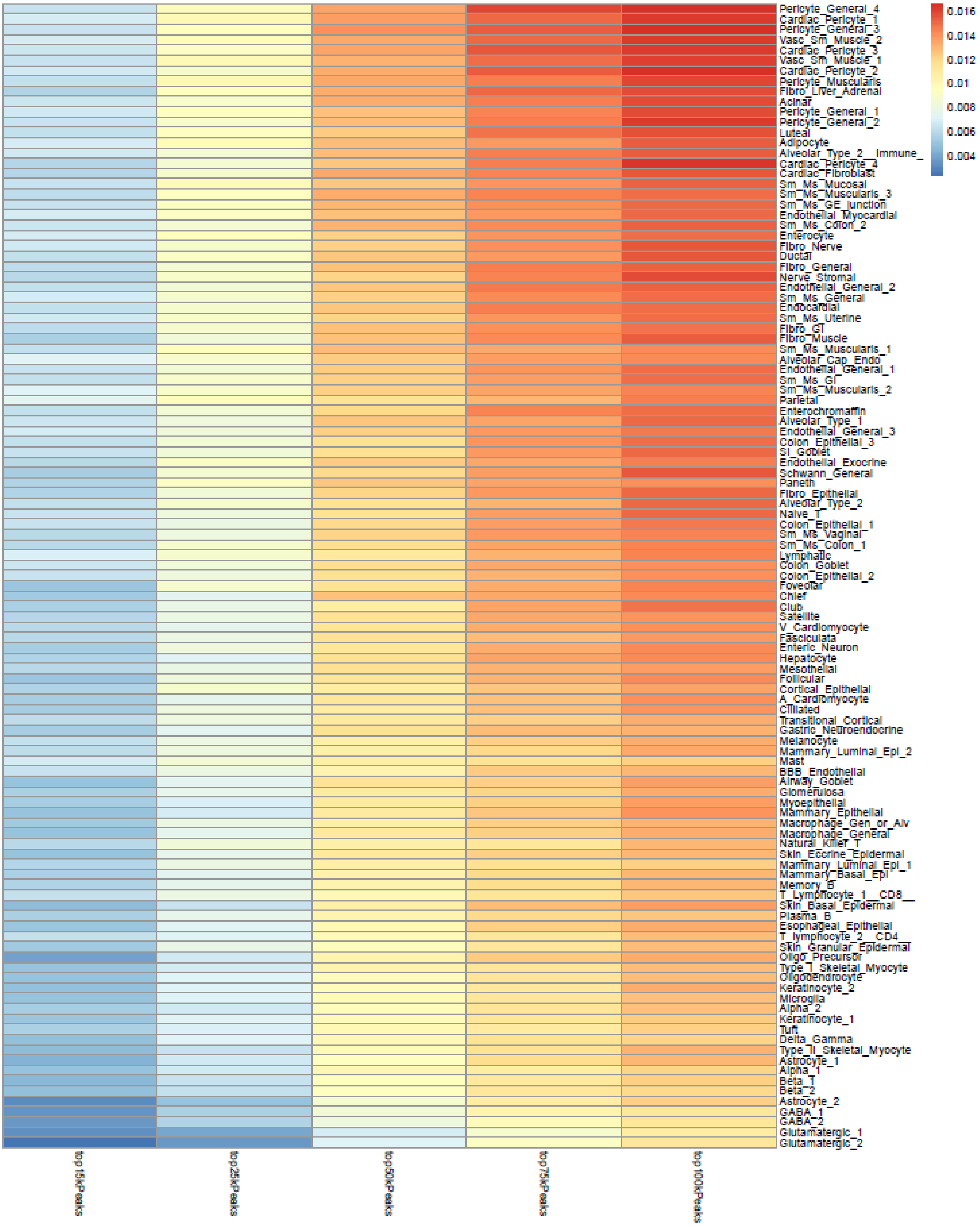

Figure S28. Evaluation of CAD PRS strength among the 111 human cell types of the adult single-cell atlas of chromatin accessibility[6]. PRS R2 (PRS strength) values are shown. For each cell type, the indicated number (either 100,000; 75,000; 50,000; 25,000 or 15,000) of strongest peaks were selected for PRS calculation. All peaks were of equal width.

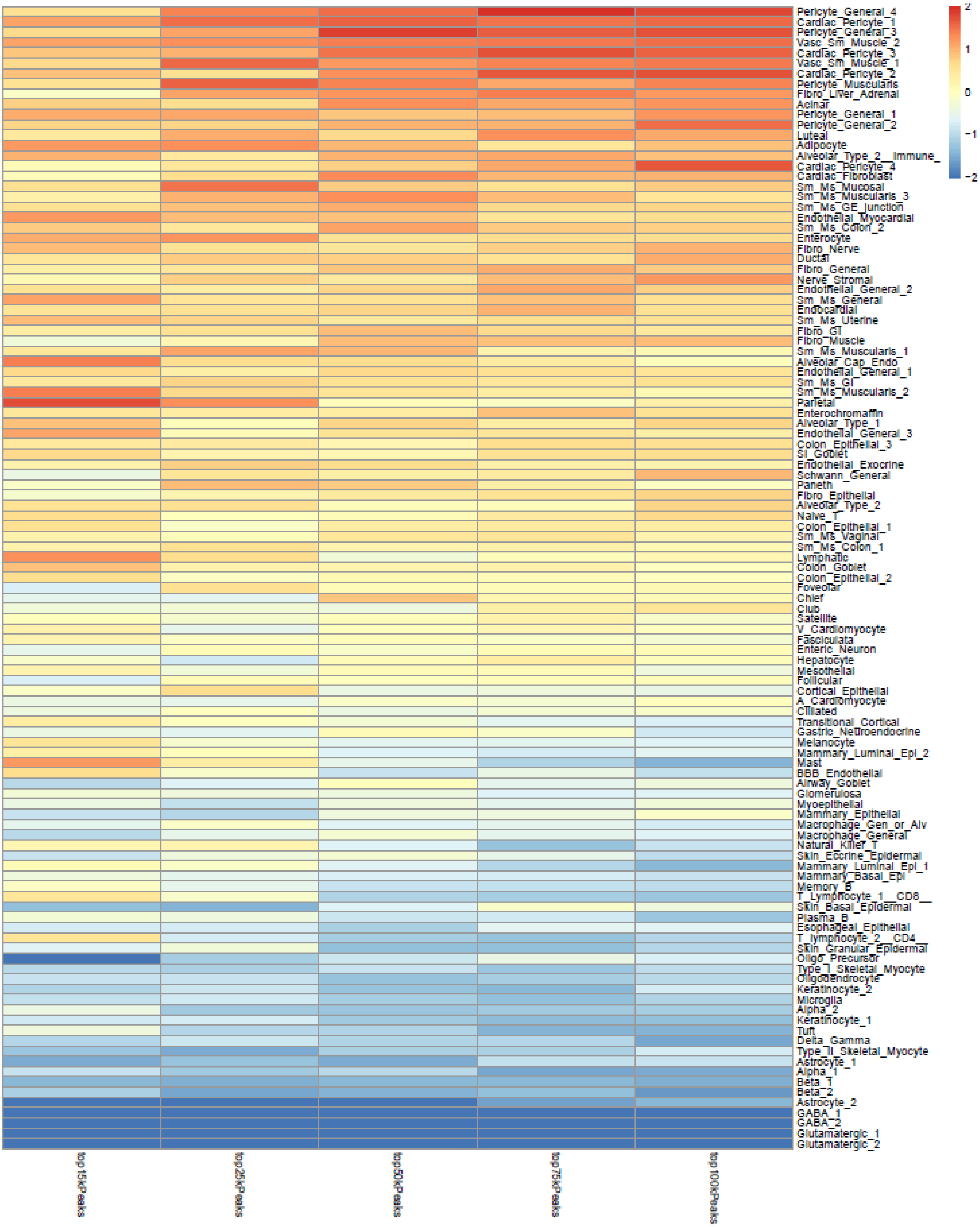

Figure S29. Evaluation of CAD PRS strength among the 111 human cell types of the adult single-cell atlas of chromatin accessibility[6]. The results are as in Figure S28, except the column z-score of the PRS R2 is shown.

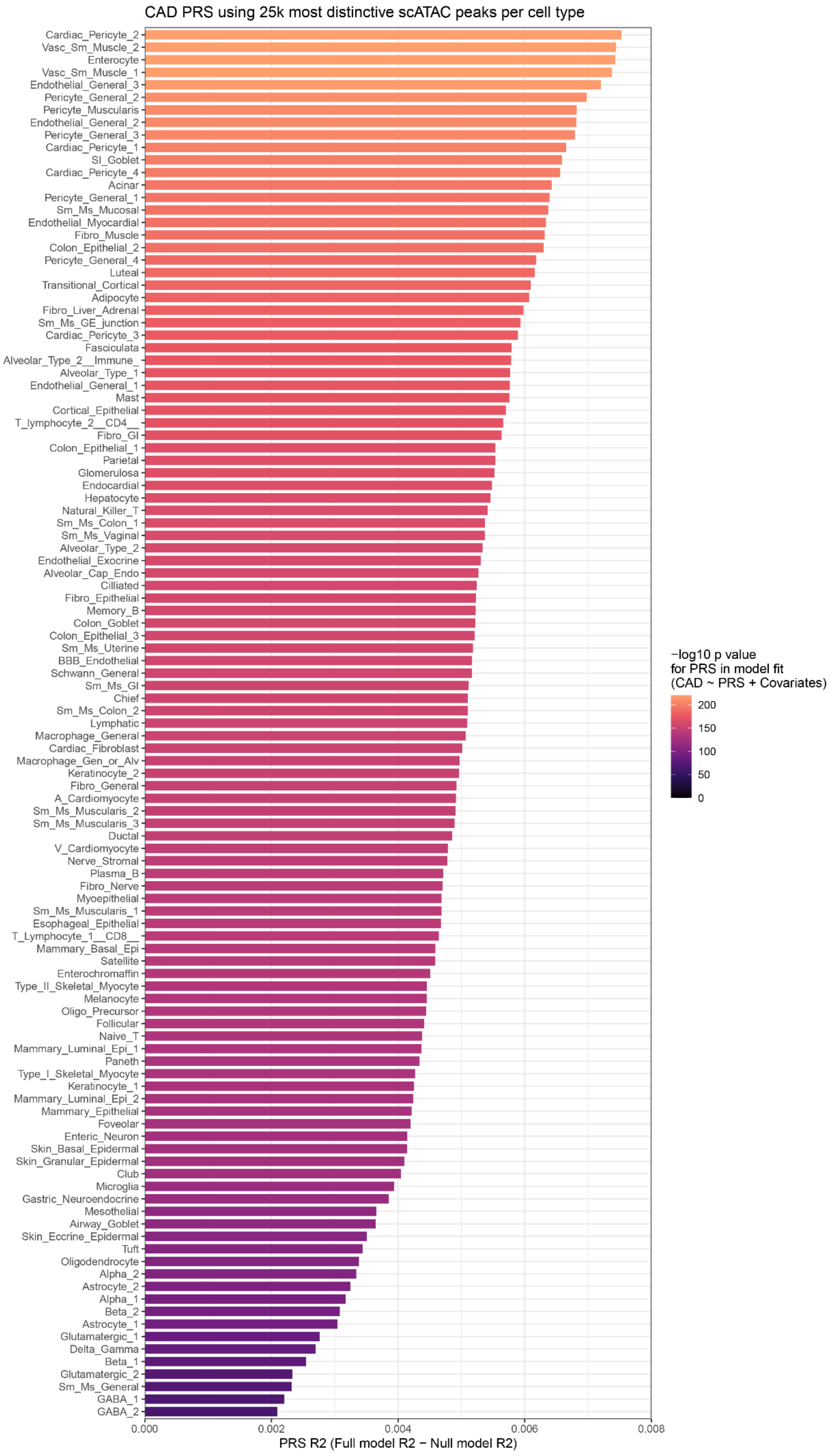
CAD PRS using 25k most distinctive scATAC peaks per cell type
Cardiac_Pericyte_2
Vasc_Sm_Muscle_2
Enterocyte
Vasc_Sm_Muscle_1
Endothelial_General_3
Pericyte_General_2
Pericyte_Muscularis
Endothelial_General_2
Pericyte_General_3
Cardiac_Pericyte_1
SI_Goblet
Cardiac_Pericyte_4
Acinar
Pericyte_General_1
Sm_Ms_Mucosal
Endothelial_Myocardial
Fibro_Muscle
Colon_Epithelial_2
Pericyte_General_4
Luteal
Transitional_Cortical
Adipocyte
Fibro_Liver_Adrenal
Sm_Ms_GE_junction
Cardiac_Pericyte_3
Fasciculata
Alveolar_Type_2__Immune_
Alveolar_Type_1
Endothelial_General_1
Mast
Cortical_Epithelial
T_lymphocyte_2__CD4__
Fibro_GI
Colon_Epithelial_1
Parietal
Glomerulosa
Endocardial
Hepatocyte
Natural_Killer_T
Sm_Ms_Colon_1
Sm_Ms_Vaginal
Alveolar_Type_2
Endothelial_Exocrine
Alveolar_Cap_Endo
Cilliated
Fibro_Epithelial
Memory_B
Colon_Goblet
Colon_Epithelial_3
Sm_Ms_Uterine
BBB_Endothelial
Schwann_General
Sm_Ms_GI
Chief
Sm_Ms_Colon_2
Lymphatic
Macrophage_General
Cardiac_Fibroblast
Macrophage_Gen_or_Alv
Keratinocyte_2
Fibro_General
A_Cardiomyocyte
Sm_Ms_Muscularis_2
Sm_Ms_Muscularis_3
Ductal
V_Cardiomyocyte
Nerve_Stromal
Plasma_B
Fibro_Nerve
Myoepithelial
Sm_Ms_Muscularis_1
Esophageal_Epithelial
T_Lymphocyte_1__CD8__
Mammary_Basal_Epi
Satellite
Enterochromaffin
Type_II_Skeletal_Myocyte
Melanocyte
Oligo_Precursor
Follicular
Naive_T
Mammary_Luminal_Epi_1
Paneth
Type_I_Skeletal_Myocyte
Keratinocyte_1
Mammary_Luminal_Epi_2
Mammary_Epithelial
Foveolar
Enteric_Neuron
Skin_Basal_Epidermal
Skin_Granular_Epidermal
Club
Microglia
Gastric_Neuroendocrine
Mesothelial
Airway_Goblet
Skin_Eccrine_Epidermal
Tuft
Oligodendrocyte
Alpha_2
Astrocyte_2
Alpha_1
Beta_2
Astrocyte_1
Glutamatergic_1
Delta_Gamma
Beta_1
Glutamatergic_2
Sm_Ms_General
GABA_1
GABA_2
0.000
0.002
0.004
0.006
0.008
PRS R2 (Full model R2 − Null model R2)
−log10 p value
for PRS in model fit
(CAD ~ PRS + Covariates)
200
150
100
50
0

Figure S30. Performance of CAD PRS derived using 25,000 most cell type-specific peaks for each of 111 cell types in the adult scATAC atlas[6]. All peaks were of equal width. The cell type peak accessibility matrix was z-scored per peak, and the highest scoring peaks were selected per cell type to obtain the most distinctive peaks.

Supplemental Table Legends

Table S1. Markers of the major cell types identified by supervised clustering.

Table S2. Markers of the 12 atherosclerosis-associated cell states.

Table S3. Differentially expressed genes identified in bulk RNA-Seq experiment. Genes are sorted by effect direction followed by statistical significance.

Table S4. Complete gene ontology listing of the cell state marker genes used to generate Figure 3C.

Table S5. Cell type marker genes for 79 human cell types based on scRNA-Seq expression profiles (https://www.proteinatlas.org/about/download). For each cell type, top 500 genes are ranked in descending order of importance.

Supplemental Methods

Differentially abundant cellular neighborhood analysis for scRNA-Seq

Milo version 1.7.1[1] was used for testing differential neighborhood abundance with a *k*-nearest neighbor graph calculated using *k* = 20 and the first 30 dimensions from PCA from Seurat.

Mouse bulk RNA-Seq differential expression and cell type decomposition

For differential expression analysis between experiment groups, lowly expressed genes were first removed using the EdgeR (version 3.24.3)[7] function filterByExpr (minimum count per sample 15, minimum total count 50). The remaining 15559 genes were used in differential expression analysis with DESeq2 (version 1.22.2)[8]. FDR < 0.05 was considered significant.

Cell type proportions in mouse aorta bulk tissue RNA-Seq profiles were estimated using the CIBERSORTx web tool (access date 2019-10-17)[9]. Aorta scRNA-Seq cells from the current study were used as the reference transcriptome profiles for bulk RNA expression profile decomposition. For generating the cell type reference signatures, single cells were annotated to the cell type level, 7 most abundant cell types were retained, cells randomly subsampled to a maximum of 1,200 cells per cell type, and genes expressed in >5 cells were retained (total 14,632 genes).

Single cell trajectory analysis

Monocle (version 2.8.0)[10] was used for pseudotime trajectory modeling following the author’s recommendations. To model the transition from contractile (*Myh11*-expressing) SMC-s to disease-increased SMC cell states, cells from the 3-month HFD library were used to avoid the need for batch correction. Contractile SMC-s were randomly subsampled to 500

cells to approximately match the number of cells in the disease-increased populations. The gene expression count preprocessing and automated cell clustering was done according to the Monocle 2 tutorial, followed by cluster marker gene detection using the Monocle differentialGeneTest function (model: '~Cluster'). The 1000 most significant genes by *p* value were used for trajectory construction using default parameters. To model the *in vitro* SMC IL-1β response trajectory, G1-phase cells (identified using Seurat cell cycle scoring with default parameters) from control and IL-1β treatment were used. Monocle 2 default processing was used, as above. As the trajectory ordering genes, all differentially expressed genes comparing treatment and control sample cells were used (Wilcoxon test, FDR < 0.05). To evaluate concordance between the *in vivo* SMC dedifferentiation trajectory and the *in vitro* SMC IL-1β response trajectory, gene set activity scores (function AddModuleScore from Seurat) were calculated for each cell and shown on cells positioned in pseudotime space. The scores were based on the 50 most significant genes by *p* value of either the *in vivo* cell state gene signature or the *in vitro* treatment differential expression.

<u>Single-cell analysis of cultured smooth muscle cells</u>

Mouse immortalized aortic smooth muscle cells (MOVAS; ATCC cell line CRL-2797) were cultured in DMEM supplemented with 10% FBS, 100 U/ml penicillin, 100 μg/ml streptomycin and 200 μg/ml geneticin in a 37 °C incubator with 5% CO_2. Cells were cultured in 12-well plates to approximately 70% confluency. Prior to experimental treatments, cells were incubated for 24 h in serum-starvation medium (DMEM supplemented with 0.2% BSA). Subsequently, the medium was replaced with serum-starvation medium supplemented with recombinant interleukin-1β (IL-1β; Sino Biological #10139HNAE) at 25 or 50 ng/ml and incubated for a further 24 or 48 h. The different IL-1β treatments were timed to end concurrently and, after trypsinization, the cells were pooled in equal counts to obtain a mixture of cells at different phases of the IL-1β response. As a control treatment, 24 h serum-

starved cells were placed in fresh serum starvation medium for a further 48 h.

The Chromium Single Cell 3' Kit (v3 Chemistry; 10xGenomics) was used to prepare scRNA-Seq libraries for control and IL-1β treated cells in separate lanes. Paired-end high-throughput sequencing was carried out on an Illumina NextSeq 550 instrument (Read 1: 28 bp, Read 2: 91 bp). Sequencing reads were processed using the Cell Ranger pipeline (version 3.0.2; 10xGenomics) and the mm10 reference transcriptome package (version 3.0.0).

Cell state gene signature activity in human plaque scRNA-seq cells and scRNA-Seq of alternative models of mouse atherosclerosis

Human coronary atherosclerosis scRNA-Seq datasets published by Wirka et al[3] (GEO: GSE131778) and mouse atherosclerosis scRNA-Seq datasets of *Apoe*$^{-/-}$ and *Ldlr*$^{-/-}$ mouse models published by Pan et al[2] (GEO: GSE155513) were analyzed to identify cells that have activated the cell state gene programs using the gene signature activity calculation implemented in the Seurat function AddModuleScore. Briefly, genes are binned based on average log expression level across samples, and, in each sample, a bin background level (calculated from random control genes in the same bin) is subtracted from the levels of the test genes.

Chromatin accessibility and gene expression by cell type for human tissues throughout the body

The adult human scATAC-Seq atlas[6] data matrix of chromatin accessibility of 111 cell types in a common peak set of approximately 890000 equal-width peaks was downloaded from Mendeley Data (DOI: 10.17632/yv4fzv6cnm.4) and the peak coordinates were lifted over from hg38 to hg19. To obtain peak profiles representative of each cell type, peaks were ranked within a cell type by accessibility signal strength and the 15000, 25000, 50000, 75000

or 100000 strongest peaks were selected.

Gene expression (TPM) profiles for 79 human cell types across the body, compiled from 30 scRNA-Seq datasets, were obtained from the Protein Atlas[11] (https://www.proteinatlas.org/about/download; access date 2023-02-10). Out of the initial 20090 genes, genes expressed at >5 TPM in at least one cell type were retained, resulting in 18043 genes. To select cell type marker genes (most distinctive genes), gene expression was first transformed into z-score per gene (gene expression relative to the variation of that gene), and then top n genes were selected within each cell type.

Immunohistochemistry

Tissue sections were blocked with 10% normal goat serum, and incubated with following primary antibodies: recombinant anti-VCAM1 antibody (ab134047, Abcam, Cambridge, UK; dilution 1:100), palladin polyclonal antibody (10853-1-AP, Proteintech, Manchester, UK; dilution 1:100), LRG1 polyclonal antibody (PA5-76287, Thermo Fisher Scientific, Waltham, MA; dilution 1:200), and monoclonal mouse anti-actin, α-smooth muscle-Cy3 (C6198, Sigma-Aldrich, St. Louis, MO; dilution 1:50 or 1:100). Biotinylated goat anti-rabbit IgG (BA-1000, Vector Laboratories, Burlingame, CA) secondary antibody and fluorescein Avidin DCS (A-2011, Vector Laboratories) were used. Nuclei were stained with DAPI (H-1200, Vector Laboratories). Imaging was performed by Zeiss LSM800 Airyscan confocal microscope with 405/488/555 nm diode lasers together with the appropriate emission filters (Plan-Apochromat 20×/0.8 objective, 1024 × 1024 and 2048 × 2048 frame sizes).

Molecular Cartography

Mice were euthanized and a full-body perfusion was executed using PBS (Gibco). Aortas and hearts were extracted on ice. The tissues were embedded in (VWR Chemicals) and then snap frozen fresh in isopentane (Fisher Scientific). The isopentane was chilled on dry

ice for 30 minutes prior to tissue freezing. The embedded tissues were kept in -70°C until sectioning. The embedded tissue samples and the Resolve Biosciences Molecular CartographyTM slides were put in the cryostat (Leica Biosystems CM1950 Cryostat, temperature at -20°C) 30 minutes prior to cryosectioning. 10 μm sections were cut and placed on the capture areas of Resolve Biosciences Molecular CartographyTM slides. The slides were packed in dry ice and sent to Resolve Biosciences for further processing. Upon arrival, tissue sections were thawed for 30 min at 37°C to improve adhesion and were fixed with 4% v/v Formaldehyde (Sigma-Aldrich F8775) in 1x PBS for 30 min at 4 °C. After fixation, sections were washed three times in 1x PBS for one min, followed by one min washes in 70% Ethanol, isopropanol, 100% Ethanol and 70% Ethanol at room temperature. Fixed samples were used for Molecular Cartography (100-plex combinatorial single molecule fluorescence in-situ hybridization) according to the manufacturer's instructions, starting with the aspiration of ethanol and incubation in Trueblack for 5 min, followed by buffer DST1, tissue priming and hybridization. Briefly, tissues were primed for 30 min at 37°C followed by 24h hybridization of all probes specific for the target genes. After the hybridizations step, samples were washed to remove excess probes and fluorescently tagged in a two-step color development process. Regions of interest were imaged as described below and fluorescent signals removed during decolorization. Color development, imaging and decolorization were repeated for multiple cycles to build a unique combinatorial code for every target gene that was derived from raw images as described below.

The probes for selected genes were designed using Resolve's proprietary design algorithm. Briefly, the probe-design was performed at the gene-level. For every targeted gene all full-length protein coding transcript sequences from the ENSEMBL database were used as design targets if the isoform had the GENCODE annotation tag 'basic'[12; 13]. To speed up the process, the calculation of computationally expensive parts, especially the off-

target searches, the selection of probe sequences was not performed randomly, but limited to sequences with high success rates. To filter highly repetitive regions, the abundance of k-mers was obtained from the background transcriptome using Jellyfish[14]. Every target sequence was scanned once for all k-mers, and those regions with rare k-mers were preferred as seeds for full probe design. A probe candidate was generated by extending a seed sequence until a certain target stability was reached. A set of simple rules was applied to discard sequences that were found experimentally to cause problems. After these fast screens, every kept probe candidate was mapped to the background transcriptome using ThermonucleotideBLAST[15] and probes with stable off-target hits were discarded. Specific probes were then scored based on the number of on-target matches (isoforms), which were weighted by their associated APPRIS level[16], favoring principal isoforms over others. A bonus was added if the binding-site was inside the protein-coding region. From the pool of accepted probes, the final set was composed by greedily picking the highest scoring probes. The following table highlights the gene names and Catalogue numbers for the specific probes designed by Resolve BioSciences with gene list name KG719.

Samples were imaged on a Zeiss Celldiscoverer 7, using the 50x Plan Apochromat water immersion objective with an NA of 1.2 and the 0.5x magnification changer, resulting in a 25x final magnification. Standard CD7 LED excitation light source, filters, and dichroic mirrors were used together with customized emission filters optimized for detecting specific signals. Excitation time per image was 1000 ms for each channel (DAPI was 20 ms). A z-stack was taken at each region with a distance per z-slice according to the Nyquist-Shannon sampling theorem. The custom CD7 CMOS camera (Zeiss Axiocam Mono 712, 3.45 µm pixel size) was used. For each region, a z-stack per fluorescent color (two colors) was imaged per imaging round. A total of 8 imaging rounds were done for each position, resulting in 16 z-stacks per region. The completely automated imaging process per round (including

water immersion generation and precise relocation of regions to image in all three dimensions) was realized by a custom python script using the scripting API of the Zeiss ZEN software (Open application development).

The algorithms for spot segmentation were written in Java and are based on the ImageJ library functionalities. Only the iterative closest point algorithm is written in C++ based on the libpointmatcher library (https://github.com/ethz-asl/libpointmatcher). As a first step all images were corrected for background fluorescence. A target value for the allowed number of maxima was determined based upon the area of the slice in µm² multiplied by the factor 0.5. This factor was empirically optimized. The brightest maxima per plane were determined, based upon an empirically optimized threshold. The number and location of the respective maxima was stored. This procedure was done for every image slice independently. Maxima that did not have a neighboring maximum in an adjacent slice (called z-group) were excluded. The resulting maxima list was further filtered in an iterative loop by adjusting the allowed thresholds for (Babs-Bback) and (Bperi-Bback) to reach a feature target value (Babs: absolute brightness, Bback: local background, Bperi: background of periphery within 1 pixel). This feature target values were based upon the volume of the 3D-image. Only maxima still in a z-group of at least 2 after filtering were passing the filter step. Each z-group was counted as one hit. The members of the z-groups with the highest absolute brightness were used as features and written to a file. They resemble a 3D-point cloud. Final signal segmentation and decoding: To align the raw data images from different imaging rounds, images had to be corrected. To do so the extracted feature point clouds were used to find the transformation matrices. For this purpose, an iterative closest point cloud algorithm was used to minimize the error between two point-clouds. The point clouds of each round were aligned to the point cloud of round one (reference point cloud). The corresponding point clouds were stored for downstream processes. Based upon the

transformation matrices the corresponding images were processed by a rigid transformation using trilinear interpolation. The aligned images were used to create a profile for each pixel consisting of 16 values (16 images from two color channels in 8 imaging rounds). The pixel profiles were filtered for variance from zero normalized by total brightness of all pixels in the profile. Matched pixel profiles with the highest score were assigned as an ID to the pixel. Pixels with neighbors having the same ID were grouped. The pixel groups were filtered by group size, number of direct adjacent pixels in group, number of dimensions with size of two pixels. The local 3D-maxima of the groups were determined as potential final transcript locations. Maxima were filtered by number of maxima in the raw data images where a maximum was expected. Remaining maxima were further evaluated by the fit to the corresponding code. The remaining maxima were written to the results file and considered to resemble transcripts of the corresponding gene. The ratio of signals matching to codes used in the experiment and signals matching to codes not used in the experiment were used as estimation for specificity (false positives). Final image analysis was performed in ImageJ using the Polylux tool plugin from Resolve BioSciences to examine specific Molecular Cartography signals.

Supplemental References

1. Dann, E., Henderson, N.C., Teichmann, S.A., Morgan, M.D., and Marioni, J.C. (2022). Differential abundance testing on single-cell data using k-nearest neighbor graphs. Nat Biotechnol 40, 245-253.
2. Pan, H., Xue, C., Auerbach, B.J., Fan, J., Bashore, A.C., Cui, J., Yang, D.Y., Trignano, S.B., Liu, W., Shi, J., et al. (2020). Single-Cell Genomics Reveals a Novel Cell State During Smooth Muscle Cell Phenotypic Switching and Potential Therapeutic Targets for Atherosclerosis in Mouse and Human. Circulation 142, 2060-2075.
3. Wirka, R.C., Wagh, D., Paik, D.T., Pjanic, M., Nguyen, T., Miller, C.L., Kundu, R., Nagao, M., Coller, J., Koyano, T.K., et al. (2019). Atheroprotective roles of smooth muscle cell phenotypic modulation and the TCF21 disease gene as revealed by single-cell analysis. Nat Med 25, 1280-1289.
4. Choi, S.W., Garcia-Gonzalez, J., Ruan, Y., Wu, H.M., Porras, C., Johnson, J., Bipolar Disorder Working group of the Psychiatric Genomics, C., Hoggart, C.J., and O'Reilly, P.F. (2023). PRSet: Pathway-based polygenic risk score analyses and software. PLoS Genet 19, e1010624.
5. Zhang, Y., Liu, T., Meyer, C.A., Eeckhoute, J., Johnson, D.S., Bernstein, B.E., Nusbaum, C., Myers, R.M., Brown, M., Li, W., et al. (2008). Model-based analysis of ChIP-Seq (MACS). Genome Biol 9, R137.
6. Zhang, K., Hocker, J.D., Miller, M., Hou, X., Chiou, J., Poirion, O.B., Qiu, Y., Li, Y.E., Gaulton, K.J., Wang, A., et al. (2021). A single-cell atlas of chromatin accessibility in the human genome. Cell 184, 5985-6001 e5919.
7. Robinson, M.D., McCarthy, D.J., and Smyth, G.K. (2010). edgeR: a Bioconductor package for differential expression analysis of digital gene expression data. Bioinformatics 26, 139-140.
8. Love, M.I., Huber, W., and Anders, S. (2014). Moderated estimation of fold change and dispersion for RNA-seq data with DESeq2. Genome Biol 15, 550.
9. Newman, A.M., Steen, C.B., Liu, C.L., Gentles, A.J., Chaudhuri, A.A., Scherer, F., Khodadoust, M.S., Esfahani, M.S., Luca, B.A., Steiner, D., et al. (2019). Determining cell type abundance and expression from bulk tissues with digital cytometry. Nat Biotechnol 37, 773-782.
10. Qiu, X., Mao, Q., Tang, Y., Wang, L., Chawla, R., Pliner, H.A., and Trapnell, C. (2017). Reversed graph embedding resolves complex single-cell trajectories. Nat Methods 14, 979-982.
11. Karlsson, M., Zhang, C., Mear, L., Zhong, W., Digre, A., Katona, B., Sjostedt, E., Butler, L., Odeberg, J., Dusart, P., et al. (2021). A single-cell type transcriptomics map of human tissues. Sci Adv 7.
12. Frankish, A., Carbonell-Sala, S., Diekhans, M., Jungreis, I., Loveland, J.E., Mudge, J.M., Sisu, C., Wright, J.C., Arnan, C., Barnes, I., et al. (2022). GENCODE: reference annotation for the human and mouse genomes in 2023. Nucleic Acids Res.
13. Yates, A.D., Achuthan, P., Akanni, W., Allen, J., Allen, J., Alvarez-Jarreta, J., Amode, M.R., Armean, I.M., Azov, A.G., Bennett, R., et al. (2020). Ensembl 2020. Nucleic Acids Res 48, D682-D688.
14. Marcais, G., and Kingsford, C. (2011). A fast, lock-free approach for efficient parallel counting of occurrences of k-mers. Bioinformatics 27, 764-770.
15. Gans, J.D., and Wolinsky, M. (2008). Improved assay-dependent searching of nucleic acid sequence databases. Nucleic Acids Res 36, e74.
16. Rodriguez, J.M., Rodriguez-Rivas, J., Di Domenico, T., Vazquez, J., Valencia, A., and Tress, M.L. (2018). APPRIS 2017: principal isoforms for multiple gene sets. Nucleic Acids Res 46, D213-D217.
